# Checkerboard-type Zhang-Rice states in overdoped cuprate superconductors

**DOI:** 10.1038/s41467-026-77985-0

**Published:** 2026-09-19

**Authors:** Xiongfang Liu, Kun Han, Yan Peng, Yuanjie Ning, Jing Wu, Zhaoyang Luo, Difan Zhou, Zhigang Zeng, Qian He, Chuanbing Cai, Mark. B. H. Breese, Ariando Ariando, Chi Sin Tang, George A. Sawatzky, Mi Jiang, Xinmao Yin

**Affiliations:** 1 Shanghai Key Laboratory of High Temperature Superconductors, Center for Quantum Science and Technology, Department of Physics, Shanghai University, Shanghai, China; 2 Information Materials and Intelligent Sensing Laboratory of Anhui Province, Institutes of Physical Science and Information Technology, Anhui University, Hefei, China; 3 School of Physical Science and Technology, Soochow University, Suzhou, China; 4 School of Electronic Science & Engineering, Southeast University, Nanjing, China; 5 Department of Physics, Faculty of Science, National University of Singapore, Singapore, Republic of Singapore; 6 Department of Materials Science and Engineering, National University of Singapore, Singapore, Republic of Singapore; 7 Singapore Synchrotron Light Source (SSLS), National University of Singapore, 5 Research Link, Singapore, Republic of Singapore; 8 Department of Physics and Astronomy, University of British Columbia, Vancouver, BC Canada; 9 Institute for Quantum Science, School of Physical Science and Technology, Soochow University, Suzhou, China

**Keywords:** Electronic properties and materials, Superconducting properties and materials

## Abstract

Cuprate superconductors remain central to condensed matter physics due to their technological relevance and unconventional, incompletely understood electronic behavior. While the canonical phase diagram and low-energy models have been shaped largely by studies of underdoped and moderately doped cuprates, the overdoped regime has received comparatively limited attention. Here, we track the evolution of the electronic structure from optimal to heavy overdoping in La_2−x_Sr_x_CuO_4_ (LSCO) using broadband optical spectroscopy across *x* = 0.15–0.60. The measured spectral changes—including the redistribution of Zhang–Rice–related spectral weight—are in qualitative agreement with determinant quantum Monte Carlo (DQMC) simulations of the three-orbital Emery model, which together indicate a pronounced reconstruction of the electronic structure beyond hole concentrations *x* > 0.2. Guided by these observations, we propose a spontaneous checkerboard-type Zhang–Rice electronic configuration that accounts for the coexistence of itinerant and localized carriers characteristic of the heavily overdoped state. Our results refine the doping-dependent Zhang–Rice–based framework for cuprates, illuminate how correlations persist deep into the overdoped regime, and provide new constraints on microscopic mechanisms of high-temperature superconductivity, with broader implications for correlated transition-metal oxides.

## Abstract (web summary)

In cuprate superconductors, the overdoped regime has received comparatively limited attention. Here, broadband optical spectroscopy and DQMC calculations reveal an electronic reconstruction in LSCO beyond x > 0.2, consistent with a checkerboard Zhang–Rice configuration hosting coexisting localized and itinerant carriers.

## Introduction

The phase diagram of cuprate superconductors has been the focus of intense investigation and debate for nearly four decades since the discovery of high-temperature superconductivity (HTS)^1^. At low doping levels, the holes predominantly occupy the Zhang-Rice Singlet (ZRS) states, effectively lowering the Fermi level (*E*_F_) to intersect the ZRS-derived band, leading to the emergence of finite conductivity. The ZRS picture was proposed as an effective low-energy building block enabling the reduction of a multi-band model to a single-band description or even a simpler *t-J* model which discards all doubly occupied states^2–4^. Superconductivity sets in at moderate doping, characterized by an unconventional ${d}_{{{{{\rm{x}}}}}^{2}-{{{{\rm{y}}}}}^{2}}$-wave pairing symmetry on the Cu sites below the transition temperature (*T*_c_)^5,6^. As hole concentration increases further into the overdoped regime, the superconducting order weakens gradually and is suppressed eventually, with the system tending towards Fermi-liquid-like behavior^7,8^. The enhancement of *T*_c_ in the underdoped regime is generally attributed to the increased superfluid density^9^. In contrast, the heavily overdoped region lies well beyond the superconducting dome and remains relatively less explored, primarily due to synthetic challenges and the scarcity of high-quality single crystals^9,10^.

Early investigations of overdoped cuprate LSCO have primarily focused on the slightly overdoped regime (0.15 <*x* ≤ 0.3) with the aim of probing the electronic structure and the mechanisms responsible for HTS^10–13^. Spectroscopic measurements reveal a saturation behavior which points to a fundamental change in the electronic structure with further hole doping^5,14^. Moreover, the validity of the ZRS approximation remains under debate, as a high-energy optical conductivity study reports a strong mixture of singlet and triplet configurations in the lightly hole-doped Zn-LSCO single crystal^15^. Meanwhile, theoretical studies have predicted the inapplicability of both the single-band Hubbard model and the ZRS framework in the overdoped regime^16^. Despite extensive studies in the slightly overdoped regime, a clear understanding of the precise evolution of the electronic structure and the validity of the ZRS model, particularly in the heavily overdoped regime (*x* > 0.3), remains elusive.

The on-site Coulomb repulsion *U*_pp_ between two electrons or two holes occupying the same ligand *p* orbital is a key parameter in many strongly correlated systems. Although it has often been omitted in effective models (e.g., the *t*–*J* model and the single-band Hubbard model), *U*_pp_ can substantially modify ligand bands and influence both structural properties and magnetic interactions^17,18^. Experimentally, the emergence, shift, or suppression of oxygen-derived spectral features reveals the interplay between *U*_pp_ and the electronic structure^19^. In cuprates, *U*_pp_ was first estimated to be around 4 eV in earlier work^20^, suggesting that its explicit inclusion is essential for understanding the evolution of the electronic structure and the validity of the ZRS picture in heavily overdoped LSCO.

**Fig. 1:**
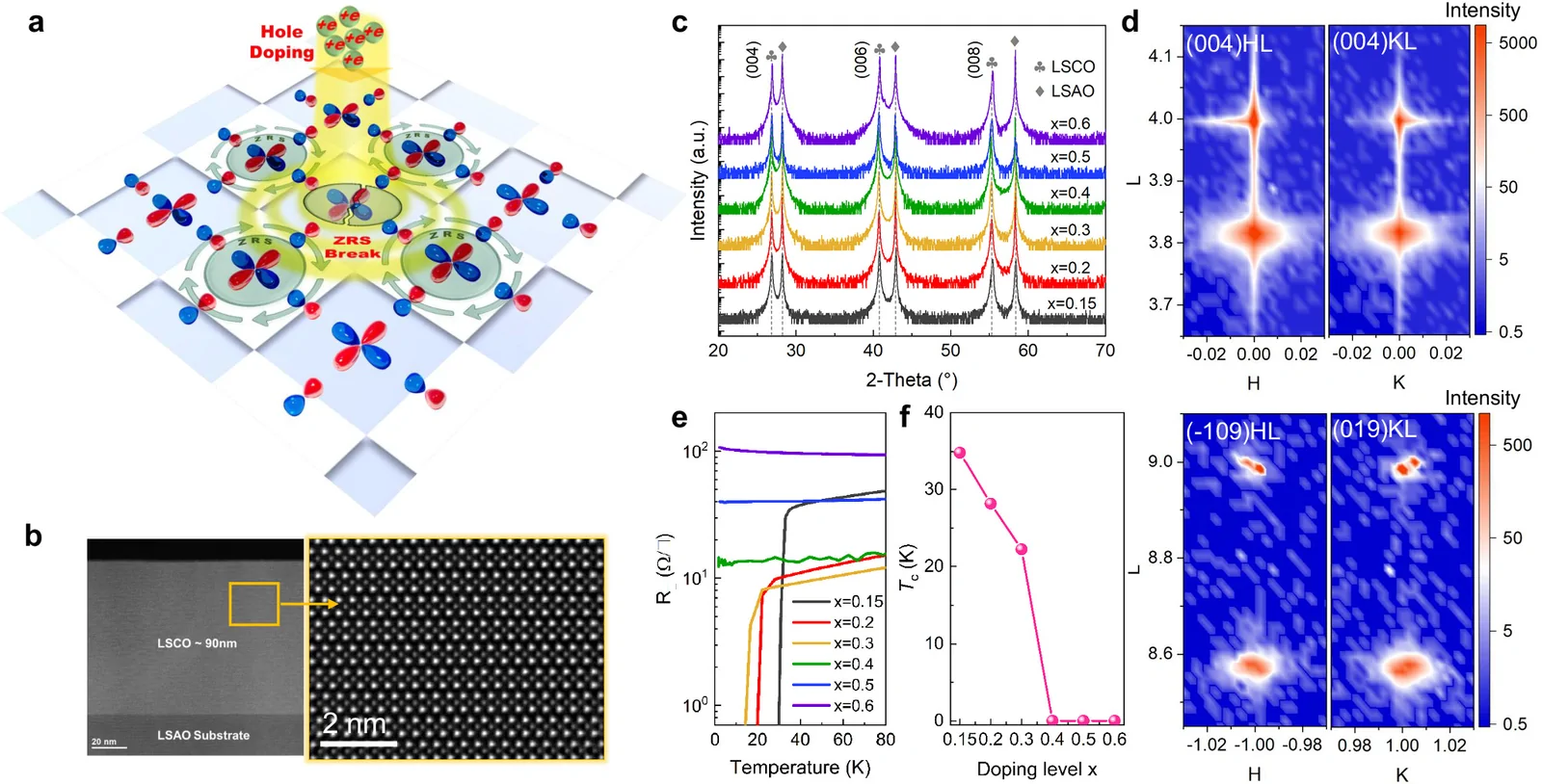
Structural characterization of LSCO thin films. **a** Schematic diagram of the checkerboard-type Zhang–Rice electronic configuration within the CuO_2_ plane in heavily overdoped LSCO.** b** Atomic-resolution HAADF-STEM images of the representative LSCO (x = 0.15)/LSAO interface along the (001) zone axis. **c** 2*θ*–*θ* scans for all LSCO (0.15 ≤ x ≤ 0.6)/LSAO via XRD. **d** RSM around (004)_*HL*_, (004)_*KL*_, (019)_*KL*_, and (−109)_*HL*_ for LSCO (x = 0.15)/LSAO as characterized via XRD. **e** Resistance versus temperature for all LSCO (0.15 ≤ x ≤ 0.6)/LSAO samples and **f** the *T*_c_ for LSCO/LSAO samples of different doping levels.

In this work, we present a comprehensive spectroscopic investigation of LSCO across an extended overdoping range (0.15 ≤ *x* ≤ 0.6). Using high-resolution X-ray absorption spectroscopy (XAS) and optical spectroscopic ellipsometry (OSE) measurements, we observe the emergence of a new spectral feature in both the O *K*-edge XAS and optical conductivity (*σ*_1_) spectra of heavily overdoped samples, which is absent in optimally and slightly overdoped LSCO. This newly identified feature points to a distinct modification of the LSCO electronic structure in which the quasiparticles can no longer be adequately described within ZRS or three-spin-polarons in the three-band model in the highly overdoped regime (Fig. 1a). In addition, our theoretical work within the three-orbital Emery model, using DQMC, indicates that the ZRS or three-spin-polarons description becomes inapplicable in the heavily overdoped regime and is consistent with experimental observation of ZRS breakdown. Furthermore, our results predict a spontaneous formation of checkerboard-type Zhang–Rice (ZR) electronic configuration in the overdoped regime, offering new insight into the evolution of electronic correlations and the suppression of superconductivity.

## Results

### Materials preparation and quality

Heavily overdoped LSCO (*x* > 0.3) bulk samples are challenging to synthesize due to the thermodynamic solubility limits and their structural instability^9^. Therefore, single-crystalline LSCO thin films with hole doping levels spanning 0.15 ≤ *x* ≤ 0.6 were synthesized on LaSrAlO_4_ (LSAO) substrates using pulsed laser deposition (PLD) (Supplementary Fig 1). All LSCO film samples have a thickness of ~90 nm.

Cross-sectional scanning transmission electron microscopy (STEM) high-angle annular dark-field (HAADF) images, shown in Fig. 1b, reveal the high crystalline quality of the films. Similarly, uniform epitaxy is observed across the entire film with no observable structural defects. Figure 1c, d and Supplementary Fig 1–6 display the 2*θ*-*θ* scans and Reciprocal Space Mappings (RSM) from X-ray diffraction (XRD) measurements for all samples. The 2*θ*–*θ* scans demonstrate that all LSCO films exhibit a (00 l)-preferred orientation and no additional peaks, indicating high phase purity and crystallinity. The RSM patterns for the (004)_*HL*_, (004)_*KL*_, (−109)_*HL*_, and (019)_*KL*_ reflections confirm the coherent lattice alignment between the LSAO substrate and the LSCO films, as well as the tetragonal symmetry of both materials (Supplementary Fig. 2)^21^. Furthermore, these observations suggest that both the in-plane and out-of-plane lattice parameters remain essentially unchanged over the wide doping range. Collectively, these characterizations establish the high crystalline quality of this LSCO series, providing a reliable platform for subsequent investigations of the optical response and electronic structure in strongly correlated cuprates.

### Transport measurement

The increase in hole concentration and the corresponding changes in *T*_c_ are well- established characteristics of LSCO^22,23^. To evaluate superconductivity across doping, the *T*_c_ for all samples was determined from electrical transport(resistivity) measurements (Fig.1e, f). The composition *x* = 0.15 corresponds to the optimally hole-doped LSCO with the highest *T*_c_ of ~35 K^24^. As Sr (hole) doping increases, *T*_c_ decreases, ending with a rapid drop above *x* = 0.3 to zero for *x* = 0.4, in a manner consistent with the canonical LSCO superconducting dome. Consequently, superconductivity becomes completely suppressed in the heavily overdoped regime and does not reemerge within the explored composition range.

### Optical analysis on electronic structure of LSCO

**Fig. 2:**
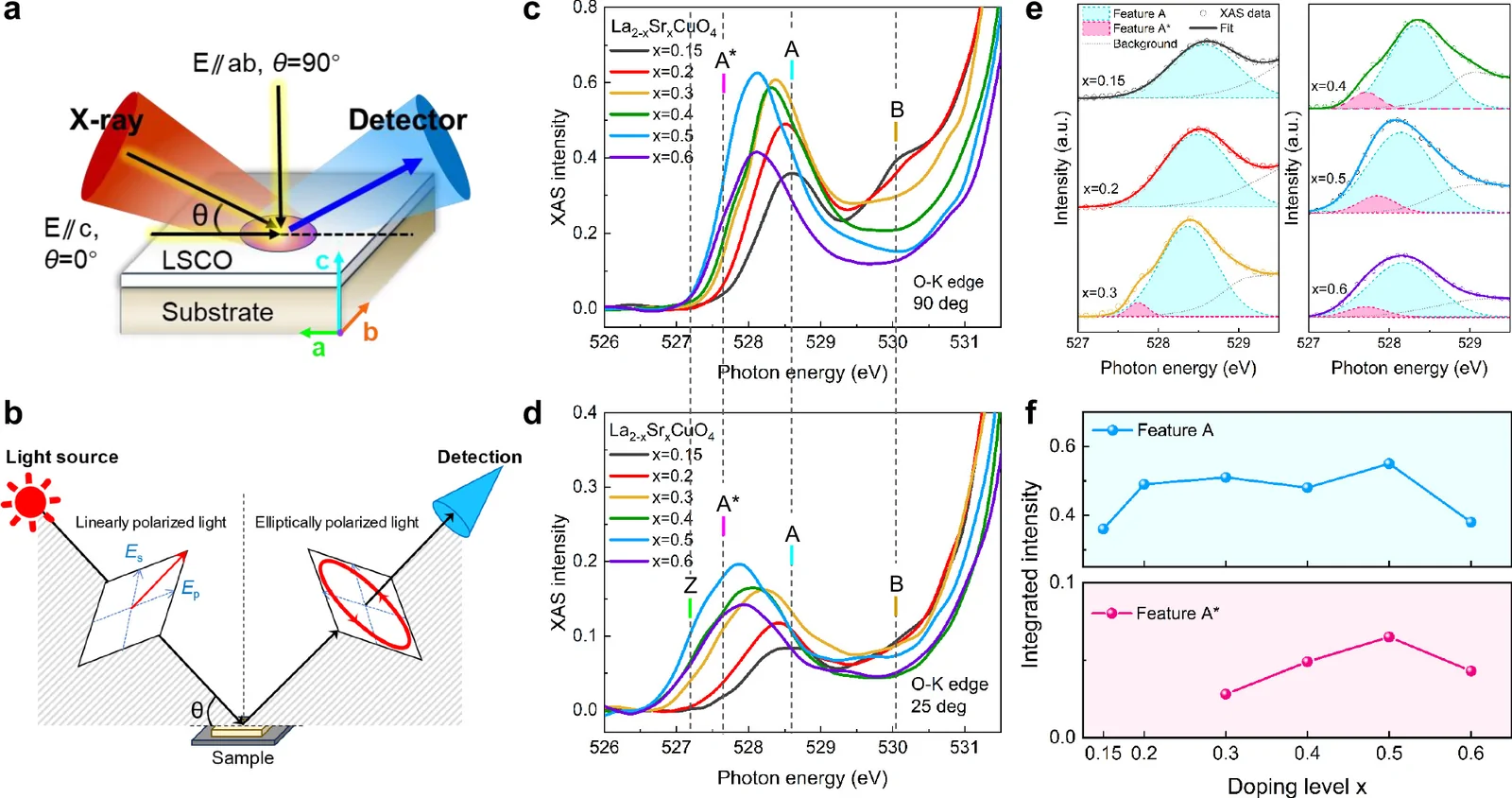
Spectroscopic investigation of electronic structure in Overdoped LSCO. **a** Schematic of XAS technique and its direction of photon polarization and incident angle. **b** Schematic of the operating principle and experimental configuration of spectroscopic ellipsometry. **c** 90° (**E**//**ab**) and (**d**) 25° (**E**//**c**) incident XAS at the O *K*-edge for LSCO/LSAO samples of different doping concentration. **e** Gaussian decomposition of the O *K*-edge XAS spectra in Fig. 2c. Features A^*^ and *A* are represented by the pink- and light-blue-filled components, respectively, while the solid curve represents the total fitted spectrum. **f** Integrated intensities of Features A^*^ and *A* extracted from the 90° O *K*-edge XAS spectra as a function of doping concentration.

The evolution of superconductivity in LSCO is driven by hole doping, which introduces additional carriers and shifts the chemical potential toward the top of the valence band, leading to increasingly metallic behavior. To further investigate the heavily overdoped regime, high-resolution XAS and OSE were employed to examine the unoccupied states and the optical response (dielectric function and optical conductivity) (Fig. 2a, b). By analyzing characteristic features in the optical spectra, we track the evolution of superconducting properties and electronic structure across hole concentrations.

### XAS characterization

Figure 2 and Supplementary Fig 7 display the O *K*-edge XAS spectra measured at 300 K for both in-plane polarization (*θ* = 90°) and out-of-plane polarization (*θ* = 25°), measured in the total-electron-yield mode. Figure 2c shows the in-plane O *K*-edge XAS spectra of LSCO for doping levels ranging from *x* = 0.15 to *x* = 0.6. These spectra exhibit doping dependence, intimately related to the O-2*p*–Cu-3*d* hybridization. Two prominent features are observed, labeled *A* (~528.6 eV) and *B* ( ~530.1 eV). Feature *A* is assigned to transitions from the O 2*p* core level to the ZRS state, while feature *B* is associated with transitions from the O 2*p* core level to the upper Hubbard band (UHB)^25,26^. With the increase in Sr doping concentration, there is a corresponding increase in the intensity of feature *A*, and along with a redshift in its energy position. Meanwhile, the intensity of feature *B* decreases, accompanied by a blueshift in photon energy. This behavior indicates spectral weight transfer from the UHB to the ZRS due to the incorporation of holes into the valence band, which is consistent with previous reports^25,26^. These changes in intensity and energy positions of features *A* and *B* begin to saturate when *x* > 0.2, while a prominent new shoulder feature labeled A^*^ (~527.8 eV) emerges for *x* > 0.2.

For comparison, Fig. 2d shows the O *K*-edge XAS spectra measured with out-of-plane polarization of LSCO for various doping levels. The out-of-plane polarization spectra inevitably contain a finite contribution from the in-plane polarization response. The spectral weight of the observed features is significantly reduced as the polarization becomes perpendicular to the CuO_2_ planes but exhibits a similar dependence on doping concentration^12^. The out-of-plane XAS spectra also display signals associated with the ZRS (feature *A*), UHB (feature *B*) and the newly identified feature A^*^. In addition, a distinct feature labeled *Z* is observed. This feature is associated with O 2*p*_z_ character, is unique to the out-of-plane geometry, and has been reported previously^27^. Interestingly, the out-of-plane XAS also registers feature A^*^, located at ~527.7 eV, at doping level *x* > 0.2, similar to the in-plane XAS measurements. This systematic evolution is further substantiated by the second-derivative and differential XAS analyses presented in Supplementary Figs. 9–11. Therefore, the observation of feature A^*^ represents a new and intriguing finding in the O *K*-edge XAS spectra.

To quantitatively characterize the doping evolution of the spectral features, the spectra were subsequently analyzed using the Gaussian decomposition shown in Fig. 2e. According to the fitting results, feature A^*^ emerges as a low-energy shoulder adjacent to feature *A* for doping level *x* > 0.2, whereas it is absent when *x* ≤ 0.2. As the doping level increases beyond 0.2, the spectral weight of feature A^*^ increases progressively. The appearance of feature A^*^ at *x* > 0.2 suggests that excessive hole doping in LSCO modifies the electronic structure, resulting in the formation of a new unoccupied state above *E*_F_. The doping dependence of the spectral weight of features A^*^ and *A* from fitting results is illustrated in Fig. 2f. Feature *A* exhibits an approximately linear doping dependence for *x* ≤ 0.2. However, in the heavily overdoped regime (*x* > 0.2), Feature *A* deviates from this linear trend, with the spectral weight decreasing in magnitude and gradually approaching saturation, indicating a weaker doping dependence. The saturation behavior of feature *A* for *x* > 0.2 is consistent with previous descriptions of XAS spectra^5,28^. The development of feature A^*^ after the saturation of feature *A* suggests a transformation in the nature of ZRS-related electronic states in the heavily overdoped regime. Similar spectral fitting results from out-of-plane O *K*-edge XAS spectra are plotted in Supplementary Fig. 12, the feature A^*^ emerges after *x* > 0.2. Meanwhile, the polarization-dependent XAS spectra at the Cu *L*_3_-edge across all doping levels of LSCO are shown in Supplementary Fig. 13. However, the most significant doping-induced changes are expected to occur in the pre-edge region of the O *K*-edge spectra.

### OSE characterization

**Fig. 3:**
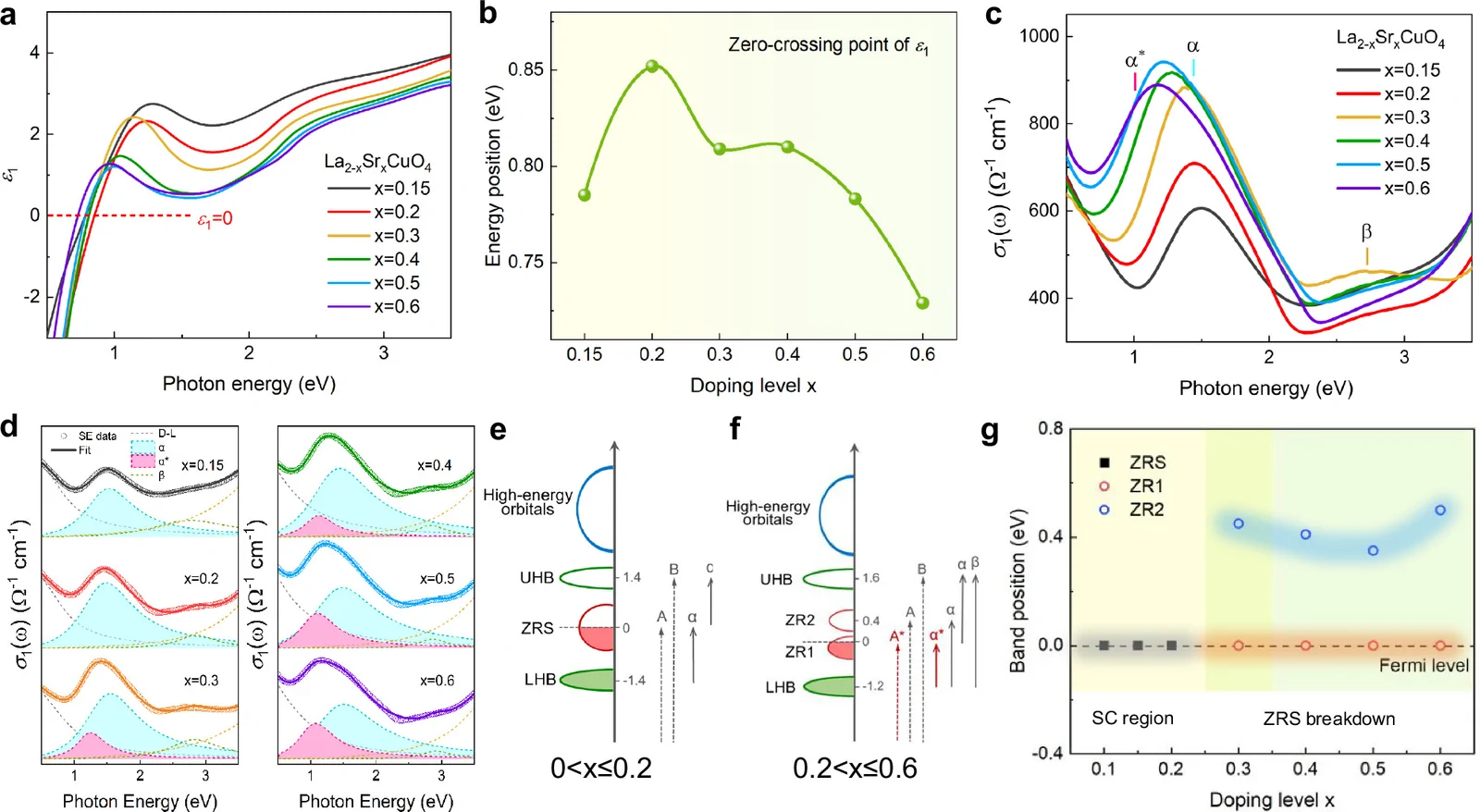
Electronic structure evolution in overdoped LSCO. **a** The real components of the dielectric function *ε*_1_, **b** *ε*_1_ zero-crossing position and, **c** optical conductivity *σ*_1_ spectra of LSCO/LSAO samples at varying doping concentration *x*. **d** Represents the Drude-Lorentz fit, which is decomposed into a Drude component and several Lorentz components, α^*^ and *α*, denoted by the pink filled and light bule filled regions, respectively. Estimated electronic structure for **e** LSCO (0<x ≤ 0.2) and **f** LSCO (0.2 <x ≤ 0.6). The arrows and the respective letters denote the interband transitions in different doped LSCO as registered by the XAS and OSE measurements previously displayed in Figs. 2c, 3c, respectively. **g** Schematic displaying the ZRS breakdown consists of clear electronic structure at *E*_F_ in heavily overdoped LSCO.

Detailed optical characterization was performed on all LSCO samples using optical spectroscopic ellipsometry (OSE) at 300 K over the photon energy range of 0.5-3.5 eV (Supplementary Fig. 4). The real and imaginary components of the dielectric function, *ε*(ω)= *ε*_1_(ω) + i*ε*_2_(ω), OSE enables the independent determination of without requiring a *Kramers*–*Kronig* transformation^29^. The real component of the dielectric function, *ε*_1_, reflects the polarizability of the material, and its spectrum is shown in Fig. 3a. The zero-crossing point in the *ε*_1_ spectrum is a characteristic signature of itinerant charge carriers and is associated with conventional metallic plasmon excitations^30^. The zero-crossing energy of *ε*_1_ increases for 0 <*x* ≤ 0.2, but decreases for *x* > 0.2 (Fig. 3b). The downward shift of *ε*_1_’s zero-crossing indicates an unexpected reduction in the effective carrier density in heavily overdoped LSCO beyond *x* = 0.2. The imaginary component, *ε*_2_, can be used to derive the optical conductivity spectrum, *σ*_1_(ω)=ω*ε*_0_*ε*_2_(ω), which provides insights into how the electrical conductivity of LSCO evolves with increasing hole doping.

The *σ*_1_ spectra in the energy range of 0.5–3.5 eV show relatively low spectral weight at *x* ≤ 0.2. Subsequently, the spectral weight increases rapidly for *x* > 0.2 (Fig. 3c). The Drude–Lorentz model was employed to fit the *σ*_1_ spectra of all LSCO samples, enabling quantitative determination of the characteristic oscillator energies and spectral weights across the doping series^31^. In addition to the dominant Drude response, a low-energy Lorentz oscillator was included to account phenomenologically for the residual finite-frequency contribution near the lower-energy limit of the measured spectral range, which is not fully reproduced by an ideal Drude term. The detailed fitting procedure and complete model parameters are provided in Supplementary Note 4.3 and Supplementary Table 3. The corresponding fitting curves are presented in Fig. 3d. For *x* ≤ 0.2, the *σ*_1_ spectrum comprises a low-energy Drude-Lorentz term plus three Lorentz features. The Drude response at *ω* = 0 corresponds to the zero-crossing point in the *ε*_1_ spectrum and reflects the metallic properties associated with delocalized carriers^32^. Feature *α* (~1.5 eV), *β* (~2.8 eV) and a higher-energy feature (>3.2 eV) appear consistently in all LSCO samples and originate from interband electronic transitions—from the ZRS to the UHB, from the lower Hubbard band (LHB) to the UHB, and from higher-energy excitations, respectively^33–35^.

Notably, an additional Lorentz oscillator associated with feature α^*^ (~1.1 eV) gradually emerges for *x* > 0.2 in the *σ*_1_ spectrum, highlighted in pink in Fig. 3d. Feature α^*^ is absent in optimally doped (*x* = 0.15) and slightly overdoped (*x* = 0.2) LSCO samples but appears in heavily overdoped LSCO (*x* > 0.2), with its intensity increasing monotonically as the doping level rises. Concurrently, the spectral weight of feature *α* transfers progressively to feature α^*^ with increasing doping, reflecting changes in the ZRS-related electronic structure.

### New electronic structure in heavily overdoped LSCO

Combined with a systematic analysis of XAS and *σ*_1_ spectra, the concurrent detection of the new feature A^*^ in the O *K*-edge XAS spectrum and the new feature α^*^ in the *σ*_1_ spectrum is an interesting finding. A point that deserves clarification is that the O *K*-edge XAS and optical conductivity measurements do not probe the same physical quantity, and therefore their spectral weights should not be expected to exhibit a strict one-to-one correspondence. The emergence of both features signals significant changes in the electronic structure of LSCO as the doping level varies. Information extracted from the features observed in the O *K*-edge XAS and *σ*_1_ spectra provides same insight into the electronic structure evolution, and the corresponding energy band diagrams for 0 <*x* ≤ 0.2 and *x* > 0.2 in LSCO are presented in Fig. 3e, f.

For LSCO samples with 0 <*x* ≤ 0.2, the ZRS state exists as a half-filled band at *E*_F_, while the UHB is unoccupied and the LHB is fully occupied (Fig. 3e). The energy separation between the UHB and LHB corresponds to the Hubbard Coulomb potential. Within this doping range, holes enter the ZRS, transforming the Mott insulating parent compound LCO into superconducting LSCO, with the highest *T*_c_ achieved at optimal doping around *x* = 0.15. Upon further hole doping beyond this level, *T*_c_ begins to decrease, while the ZRS remains relatively stable in the range 0 <*x* ≤ 0.2. This scenario is consistent with previous studies^12,25,35^.

However, as the hole doping concentration continues to increase, the ZRS spectral weight begins to saturate and overlap, leading to significant interactions and interference at high hole densities. This enhanced hole density on the oxygen sites amplifies the additional Coulomb repulsion *U*_pp_ on the oxygen sites when two holes occupy orbitals on the same oxygen atom^5^. The heavily overdoped system becomes less stable energetically as *U*_pp_ grows, destabilizing the ZRS manifold. Once the ZRS manifold is exhausted, new electronic states emerge. Consequently, the ZRS begins to break down, giving rise to two distinct electronic states, here labeled “Zhang-Rice feature 1 (ZR1)” and “Zhang-Rice feature 2 (ZR2)”. The ZR1 band remains near *E*_F_, while the ZR2 band shifts to higher energies above *E*_F_ (Fig. 3f). The transformation of the originally broad ZRS feature into a narrower ZR1 band near *E*_F_ implies increased charge localization, leading to a concomitant reduction in the effective carrier density in the heavily overdoped regime, as shown in Fig. 3b. In addition, the emergence of two distinct electronic states, ZR1 and ZR2, respectively, provides experimental support for an alternating ZR electronic motif in the electronic landscape. The new feature A^*^ observed in the XAS can be attributed to transitions into the partially unoccupied ZR1 band, whereas feature α^*^ detected in the *σ*_1_ spectrum originates from electronic transitions between the LHB and the half-filled ZR1 band. The evolution of the band positions near *E*_F_ as a function of doping is illustrated in Fig. 3g. This diagram clearly demonstrates the reconstruction and splitting of the ZRS-derived states in heavily overdoped LSCO.

### Simulating three-orbital Emery model with determinant quantum Monte Carlo (DQMC)

**Fig. 4:**
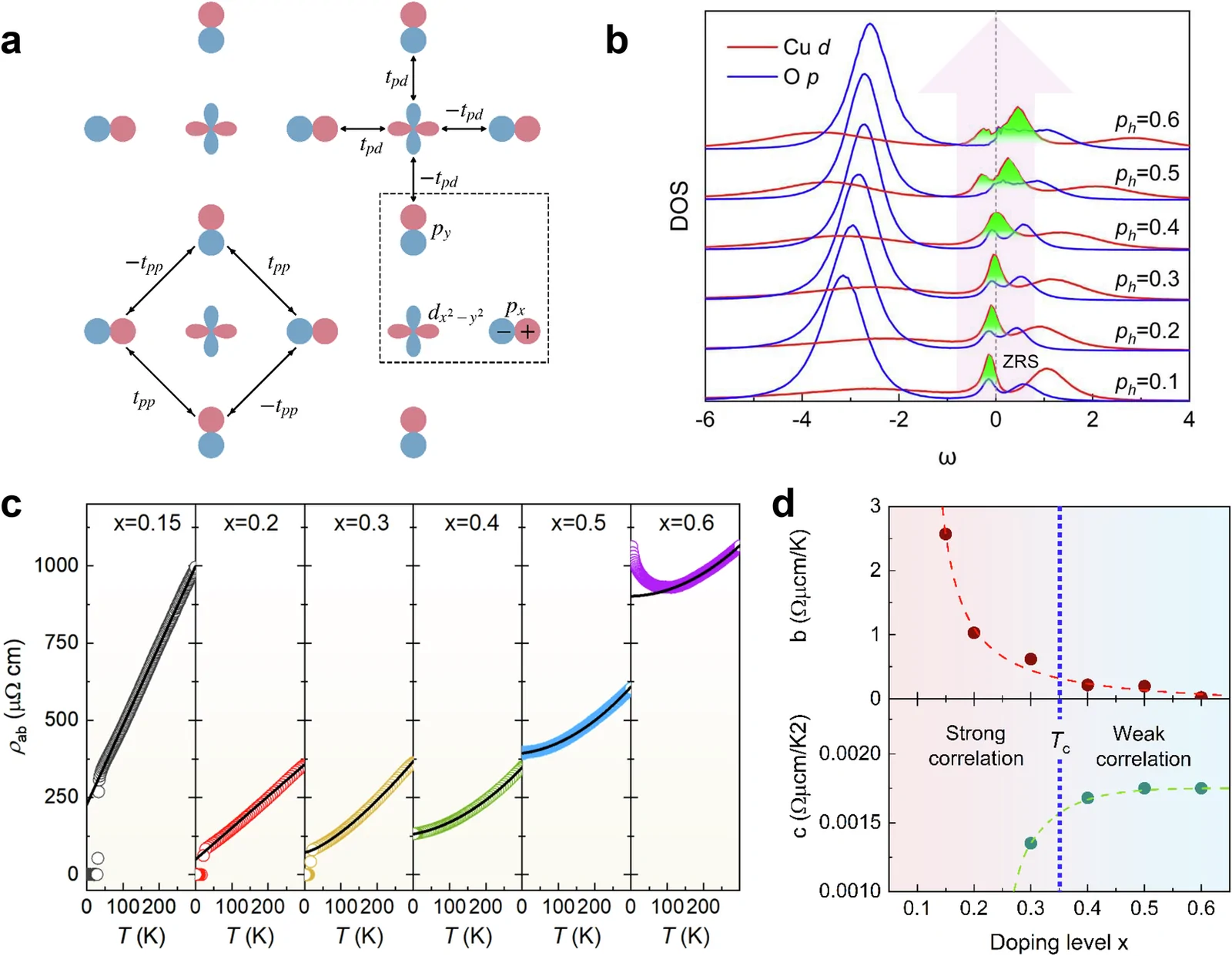
DQMC simulation and electronic state analysis of Overdoped LSCO. **a** Schematic illustration of the three-orbital model. The black dashed box denotes the unit cell of the model, with the orbital types and corresponding wave-function phases indicated. The phase conventions for the hopping parameters ${t}_{{pp}}$ and ${t}_{{pd}}$ in hole language are also explicitly specified. **b** Simulated DOS for LSCO with varying doping levels n. The Red (blue) solid line represents Cu (O) orbitals. **c** The Fitting results of the in-plane resistivity of LSCO with varying doping levels, whereas the colorful scatters are corresponding to *ρ*(*T*) values by transport measurements and the black solid lines represent fits to the data below 300 K using the expression $\rho (T)={{{\rm{a}}}}+{{{\rm{b}}}}T+{{{\rm{c}}}}{T}^{2}$. **d** Doping evolution of the coefficients b (T-linear resistivity component) and c (*T*^2^ resistivity component) of expression $\rho (T)=a+{bT}+c{T}^{2}$.

To further investigate the origin of the anomalous low-energy behavior, we perform numerically unbiased determinant quantum Monte Carlo (DQMC) simulations within the three-orbital Emery model (Supplementary Fig. 7)^36^. As illustrated in Fig. 4a, the three-orbital model includes only ${d}_{{x}^{2}-{y}^{2}}$ and ${p}_{x,y}$ orbitals, with the hole-language hopping phase conventions indicated. Without assuming the existence of ZRS in advance, the three-orbital model serves as a minimal model that captures the essential physics without imposing a priori ZRS^37^. We use a canonical parameter set with the *p*-*d* hopping *t*_pd_ taken as the energy unit. The Hamiltonian includes on-site Coulomb repulsion *U*_dd_ on the Cu *d*-orbitals and *U*_pp_ on the O *p*-orbitals respectively, and reads as follows (Supplementary Fig. 7):

1 $$\hat{H}=\hat{E}^{s}+\hat{K}+\hat{U}$$

2 $$\hat{E}^{s}=\left({\epsilon }_{d}-\mu \right){\sum }_{i\sigma }{\hat{n}}_{i\sigma }^{d}+\left({\epsilon }_{p}-\mu \right){\sum }_{j\sigma }{\hat{n}}_{j\sigma }^{p}$$

3 $$\hat{K}={\sum }_{\left\langle {ij}\right\rangle \sigma }{t}_{{pd}}^{{ij}}\left({\hat{d}}_{i\sigma }^{{{\dagger}} }{\hat{p}}_{j\sigma }+h.c.\right)+{\sum }_{\left\langle j{j}^{{\prime} }\right\rangle \sigma }{t}_{{pp}}^{j{j}^{{\prime} }}\left({\hat{p}}_{j\sigma }^{{{\dagger}} }{\hat{p}}_{{j}^{{\prime} }\sigma }+h.c.\right)$$

4 $$\hat{U}={U}_{{dd}}{\sum }_{i}{\hat{n}}_{i\uparrow }{\hat{n}}_{i\downarrow }+{U}_{{pp}}{\sum }_{j}{\hat{n}}_{j\uparrow }{\hat{n}}_{j\downarrow }$$

The descriptions of parameters and their detailed values are listed in Supplementary Table 4. Here, the parameters of primary interest are *U*_pp_ and the inverse temperature *β*. Figure 4b shows the local density of states (DOS), extracted via the maximum entropy analytic continuation (MaxEnt)^38,39^, over a substantial hole-doping range *p*_h_ from 0.1 to 0.6. As the doping level, *p*_h_, increases, the red Cu-derived peak above *E*_F_, commonly associated with the UHB, moves toward higher energy with spectral weight significantly reduced^40^. The broad feature centered near ~3 eV is the charge-transfer band located below *E*_F_. Due to a relatively high simulation temperature, the LHB is largely broadened. In the simulated hole doping range of *p*_h_ ≤ 0.4, the ZRS remains intact and shows strong *p*-*d* hybridization. Nonetheless, as the doping level reaches *p*_h_ = 0.5, the spectra show split features, supporting the optical observation of ZRS breakdown. Supplementary Fig. 21 corresponds to different values of *U*_pp_ ranging from 0 to 2, or inverse temperatures *β* equal to 8 or 10. A systematic decrease in *U*_pp_ leads to a noticeable reduction in the critical hole concentration at which the low-energy spectral peak begins to split, indicating that *U*_pp_ quantitatively influences the onset of the spectral reconstruction.

Since the ZRS is formed by the linear combination of one hole on the Cu 3 *d* orbital and the other on the four ligand O-2*p* orbitals, it is sensitive to the hole densities on Cu and O^3^. In the charge transfer regime, the doped holes mainly reside on O orbitals due to the higher repulsion on Cu orbitals. This may lead to a gradual deviation of Cu-O hole concentration and thereby strongly affect the stability of the ZRS^3^. As a result, the Cu-O coupling strength in the UHB and the ZRS is substantially weakened, especially when *p*_h_ exceeds 0.4. More detailed investigation of the ${{{\boldsymbol{k}}}}$-resolved spectral dispersion has been conducted in a recent study^41^. Although direct calculation of real-frequency spectral quantities within DQMC remains challenging, the local density of states obtained from our simulations reproduces the essential electronic-structure evolution observed experimentally, thereby supporting the conclusion of electronic reconstruction.

## Discussion

### Anomalous electrical resistivity of heavily overdoped LSCO

According to the established LSCO phase diagram, the system is commonly understood to evolve from strange-metal to Fermi-liquid behavior as the doping level increases from *x* = 0.15 to 0.6^8^. Therefore, the resistivity behavior of LSCO changes significantly across different doping levels. The expression $\rho (T)={{{\rm{a}}}}+{{{\rm{b}}}}T+{{{\rm{c}}}}{T}^{2}$ is an appropriate model for describing the resistivity of overdoped LSCO samples (Supplementary Fig. 6). The parallel-resistivity fits (solid black curves in Fig. 4c) are nearly indistinguishable from the experimental *ρ*(*T*) curves over the range 0.15 ≤ *x* ≤ 0.6. Resistivity decreases with increasing *x* up to 0.4, then rises at higher overdoping levels, consistent with previous studies on LSCO^42,43^. A distinct linear *ρ* − *T* relationship is observed down to *T*_c_ in LSCO (0.15 ≤ *x* ≤ 0.2) samples, following the simplified relation $\rho (T)={{{\rm{a}}}}+{{{\rm{b}}}}T$ with c = 0. The persistence of this strange-metal behavior up to 300 K likely reflects the presence of strong electronic correlations. Nevertheless, at higher doping levels (*x* > 0.2), the *ρ*(*T*) curves exhibit pronounced nonlinear behavior that is well described by the full expression $\rho (T)={{{\rm{a}}}}+{{{\rm{b}}}}T+{{{\rm{c}}}}{T}^{2}$.

The doping evolution of the coefficients b and c obtained from corresponding *ρ* − *T* fits is shown in Fig. 4d. The coefficients b, associated with strong electron-electron scattering, is observed to decrease rapidly with increasing *x*. Notably, b tends toward a low value as *T*_c_ disappears, suggesting that strong electronic correlations are intimately linked to superconductivity^7^. Conversely, as the coefficient *b* decreases and *c* grows in the fit $\rho (T)={{{\rm{a}}}}+{{{\rm{b}}}}T+{{{\rm{c}}}}{T}^{2}$, the system exhibits a gradual crossover toward Fermi‑liquid–like behavior that extends deep into the heavily overdoped regime. Nevertheless, the resistivity deviates from the canonical Fermi‑liquid form $\rho (T)\propto {T}^{2}$ due to the coexistence of linear and quadratic temperature components. At the highest dopings (*x* > 0.5), LSCO may even approach a metal–insulator transition. Previous studies of bosonic excitations in cuprates have also demonstrated similar *ρ*-*T* relationships and doping dependences^8,44^.

### ZRS breakdown and electronic correlations

Taken together with our new findings on the electronic structure of heavily overdoped LSCO (Fig. 3e, f), the simple picture of “one additional ZRS hole per Sr dopant” no longer provides an adequate description. Instead, the extra holes modify the electronic structure in a fundamentally different way, consistent with a breakdown of the conventional ZRS framework. The crossover to a nonlinear resistivity regime appears to occur concomitantly with the breakdown of the ZRS. The heavily overdoped regime therefore hosts distinct many-body phenomena. With increasing *x*, resistivity becomes increasingly nonlinear while the superconducting *T*_c_ decreases—suggesting a progressive weakening of the strong electronic correlations accompanying the breakdown of the conventional ZRS framework^34^.

The breakdown of the ZRS occurs concurrently with the reduction of strong correlations in overdoped cuprates. This may suggest that the carriers participating in superconductivity become less effective at engaging in spin fluctuations and Cooper pairing, effectively reducing both the superfluid density and the pairing strength^10,45–47^. Consequently, *T*_c_ diminishes and eventually vanishes. This weakened‑correlation scenario drives heavily overdoped LSCO toward a state characterized by nonlinear resistivity.

Our combined XAS and OSE measurements reveal significant electronic structure changes in heavily overdoped LSCO. This is evidenced by the appearance of distinct spectral features at doping levels *x* > 0.2, signaling the saturation and destabilization of the ZRS picture. This ZRS breakdown is accompanied by the formation of new, reduced-correlation bands, reflected in both spectroscopic signatures and coinciding with a doping-induced crossover in resistivity from linear to nonlinear behavior. These observations indicate that the heavily overdoped regime cannot be described solely by the traditional strong-correlation ZRS framework and instead evolves toward a moderate-correlation state.

Further analysis suggests that the originally broad ZRS feature progressively evolves into two narrower subbands, denoted here as ZR1 and ZR2. The ZR1 band, located near the *E*_F_, retains the original ZRS-derived character, whereas the ZR2 band is more localized and exhibits a diminished ZRS contribution. This ZRS breakdown reflects the increasing differentiation of the electronic states in the heavily overdoped regime. Consistent with this, the observation of a dual-component electrical resistivity indicates the coexistence of two distinct electronic subsystems within the same CuO_2_ plane. Such coexistence implies that heavily overdoped cuprates may favor an inhomogeneous correlated state and cannot be regarded as a conventional Fermi liquid^7^.

In the heavily overdoped regime of approximately 0.5 hole per O square, one can imagine a spontaneous configuration in which half of the Cu sites host a ZRS (3*d*^9+^ hole, S = 0) while the remaining half retain the Cu^2+^-like (3*d*^9^, S = 1/2) character. These two types of Cu sites may arrange alternatively to form a checkerboard-type electronic pattern, as illustrated in Fig. 1a. In such a configuration, one sublattice consists of O squares each hosting a hole that forms a ZRS with the central Cu of ${d}_{{{{{\rm{x}}}}}^{2}-{{{{\rm{y}}}}}^{2}}$ symmetry, while the other sublattice comprises Cu^2+^-like sites without additional holes. At precisely 0.5 hole doping, this alternating arrangement would likely yield an insulating system (probably related to the transport results of LSCO (*x* = 0.6) in Fig. 4c). With further hole doping, the additional holes are expected to occupy oxygen orbitals with s-like ${d}_{{{{{\rm{x}}}}}^{2}+{{{{\rm{y}}}}}^{2}}$ symmetry around the Cu^2+^-like sites and hybridize with the ${3d}_{{{{{\rm{z}}}}}^{2}-{{{{\rm{r}}}}}^{2}}$ orbital, leading to both in-plane and out-of-plane polarization features at the O absorption edge. However, XAS cannot distinguish between ${d}_{{{{{\rm{x}}}}}^{2}+{{{{\rm{y}}}}}^{2}}$ and ${d}_{{{{{\rm{x}}}}}^{2}-{{{{\rm{y}}}}}^{2}}$ orbital combinations. We note that the present DQMC simulations do not provide clear evidence for non-uniform real-space charge distribution. This may reflect the limitations imposed by the currently accessible temperature range and system size, under which subtle ordering tendencies can be significantly suppressed.

Therefore, hole-doped cuprates may exhibit two characteristic regimes of hole incorporation. At low doping, holes propagate predominantly as ZRS or three‑spin polaron states; near 0.5 holes per O square, the electronic structure appears to evolve toward a checkerboard‑type configuration with alternating ZRS and Cu^2+^‑like sites. In principle, intermediate phases with varying proportions of ZRS and Cu^2+^‑like character could exist between these limits. This hypothetical alternating configuration finds an intriguing parallel in the nickelates, where recent resonant X-ray scattering measurements on NdNiO_3_ have revealed alternating Ni sites with large spin moments and strong antiferromagnetic Ni–O coupling^48^. Such a bond-disproportionated state, characterized by alternating Ni–O ZRS–like and Ni^2+^-like sites, provides a compelling analog for understanding how heavily overdoped cuprates might undergo electronic phase separation or bond disproportionation at extreme doping levels.

The overdoped regime of the cuprate phase diagram, once regarded as a conventional Fermi liquid limit, is increasingly recognized as a key frontier for understanding correlated electron behavior. At high doping, the system undergoes a dramatic reconstruction of the electronic structure while retaining vestigial correlation effects. Meanwhile, overdoped cuprates exhibit a subtle interplay between itinerant and localized carriers, suggesting proximity to electronic phase separation states.

Importantly, the overdoped regime offers a unique opportunity to probe the intrinsic mechanism of superconductivity under conditions where competing pseudogap and antiferromagnetism phenomena are largely suppressed. By studying how the pairing strength, coherence, and carrier dynamics evolve across the overdoped regime, one may isolate the essential electronic interactions responsible for superconducting condensation. Looking forward, systematic investigations combining high-resolution spectroscopies (RIXS, ARPES, and ultrafast optical probes) with quantum many-body calculations will be crucial to elucidate how the checkerboard-type electronic pattern evolves beyond the optimal doping level. Ultimately, the heavily overdoped cuprates provide a clean, tunable platform for understanding the relationship between electronic structure reconstruction and superconductivity in cuprates.

## Methods

### Sample preparation

A series of heavily overdoped La_2-x_Sr_x_CuO_4_ (LSCO, 0.15 ≤ *x* ≤ 0.6) thin films were grown on (001)-oriented LaSrAlO_4_ (LSAO) single-crystal substrates using pulsed laser deposition (PLD). A KrF excimer laser operating at a repetition rate of 3 Hz and a pulse energy of 300 mJ was employed. The substrate temperature was maintained at 720 °C under an oxygen partial pressure of 20 Pa. After 6000 laser pulses, LSCO films with a thickness of approximately 90 nm were obtained. Post-deposition annealing was carried out in an oxygen atmosphere at 550 °C, with the oxygen pressure increased to 2000 Pa.

### Structural characterization and electrical transport measurements

The crystalline structure and epitaxial relationship of the LSCO films were characterized by high-resolution X-ray diffraction (XRD) at the X-ray demonstration and development (XDD) beamline at the Singapore Synchrotron Light Source (SSLS). Reciprocal space mappings (RSM) were collected in a coplanar diffraction geometry around the selected (004)_*HL*_, (004)_*KL*_, (−109)_*HL*_, and (019)_*KL*_ reflections to evaluate the epitaxial relationship and lattice structure.

Temperature-dependent electrical transport measurements were performed using a Keithley 2400 SourceMeter and a Keithley 2002 digital multimeter. The resistance was measured as a function of temperature to determine the superconducting transition and to characterize the doping-dependent normal-state transport behavior. The detailed analysis of the temperature-dependent resistivity is described in Supplementary Note 6.

### X-ray absorption spectroscopy

X-ray absorption spectroscopy (XAS) measurements were performed at the Soft X-ray-ultraviolet (SUV) beamline at the Singapore Synchrotron light source. The spectra were acquired in total-electron-yield (TEY) mode in an ultrahigh-vacuum chamber with a base pressure of approximately 1 × 10^−9^ mbar. All experiments were conducted at room temperature.

Polarization-dependent O *K*-edge spectra were collected using two incidence geometries corresponding to predominantly in-plane and out-of-plane responses. Spectra acquired at $\theta={90}^{\circ }$ and $\theta={25}^{\circ }$ were used to probe the anisotropic spectral features. Polarization-dependent Cu *L*_3_-edge spectra were acquired using the same experimental configuration. Details of the spectral normalization and analysis are provided in Supplementary Note 3.

### Spectroscopic ellipsometry measurement

Variable-temperature spectroscopic ellipsometry (SE) measurements were performed using a custom-built J.A. Woollam Co., Inc. Variable Angle Spectroscopic Ellipsometer (VASE) over a photon energy range of 0.5–3.5 eV, with an optimal incident angle of 70° relative to the sample normal. The measurements were carried out in a high-vacuum chamber (~10⁻⁹ mTorr) across a temperature range of 5 K to 300 K, encompassing both the superconducting and normal states. Details of the optical model, fitting procedure, and complete fitting parameters are provided in Supplementary Note 4.

### DQMC and maximum entropy method

DQMC is a numerically exact finite-temperature many-body method, up to statistical and finite-size effects, in which the interacting fermionic problem is mapped onto a statistical ensemble of auxiliary-field configurations through a Hubbard-Stratonovich transformation. For each auxiliary-field configuration, the fermionic degrees of freedom can be integrated out, yielding a determinant weight that can be sampled stochastically. This formulation allows electron-electron interactions to be treated non-perturbatively. The direct output of DQMC is the single-particle Green’s function in imaginary time, whereas spectroscopic experiments probe the corresponding spectral properties in the real-frequency domain. The orbital-resolved spectral function ${A}_{{{{\rm{\alpha }}}}}\left({{{\rm{\omega }}}}\right)$ was therefore obtained by analytically continuing the imaginary-time Green’s function using the maximum entropy method (MEM) via

5 $${G}_{\alpha }(\tau )={\int }_{-\infty }^{\infty }\,d\omega \frac{{e}^{-\tau \omega }}{1+{e}^{-\beta \omega }}{A}_{\alpha }(\omega ),$$

where ${A}_{\alpha }\left(\omega \right)$ denotes the spectral function associated with orbital α. Since the inversion of this integral equation is an ill-posed problem, MEM regularizes the analytic continuation by incorporating prior information and selecting the spectral function that provides the most probable reconstruction consistent with the QMC data. This procedure suppresses spurious structures arising from statistical noise while retaining the dominant spectral features. Momentum-resolved spectral functions ${A}_{\alpha }\left({{{\boldsymbol{k}}}},\omega \right)$ were obtained analogously from the corresponding momentum-space Green’s functions. Statistical error bars are not shown for the MEM spectra because the nonlinear analytic continuation makes a straightforward propagation of the QMC statistical uncertainties unreliable.

## Supplementary information

Supplementary Information

Transparent Peer Review file

## Source data

Source Data

## Data Availability

The data that support the findings of this study are available from the corresponding authors upon request. Source data are provided with this paper.
