# Supplementary material for: Checkerboard-type Zhang-Rice states in overdoped cuprate superconductors
Source: Nat Commun. 2026 Sep 19;17:10975. doi: 10.1038/s41467-026-77985-0 (PMC13683379; doi:10.1038/s41467-026-77985-0)
Supplement: Supplementary file 1 [file 41467_2026_77985_MOESM1_ESM.pdf]

Supplementary Information

Checkerboard-type Zhang-Rice States in Overdoped Cuprate Superconductors

Xiongfang Liu[1,#], Kun Han[2,#], Yan Peng[3,#], Yuanjie Ning[1,#], Jing Wu[4], Zhaoyang Luo[5], Difan Zhou[1], Zhigang Zeng[1], Qian He[6], Chuanbing Cai[1], Mark. B. H. Breese[5,7], Ariando Ariando[5], Chi Sin Tang[7,*], George A. Sawatzky[8,*], Mi Jiang[3,9,*], Xinmao Yin[1,*]

[1] Shanghai Key Laboratory of High Temperature Superconductors, Center for Quantum Science and Technology, Department of Physics, Shanghai University, Shanghai 200444, China

[2] Information Materials and Intelligent Sensing Laboratory of Anhui Province, Institutes of Physical Science and Information Technology, Anhui University, Hefei 230601, China

[3] School of Physical Science and Technology, Soochow University, Suzhou 215006, China

[4] School of Electronic Science & Engineering, Southeast University, Nanjing, 211189, China

[5] Department of Physics, Faculty of Science, National University of Singapore, Singapore 117551, Republic of Singapore

[6] Department of Materials Science and Engineering, National University of Singapore, Singapore 117575, Republic of Singapore

[7] Singapore Synchrotron Light Source (SSLS), National University of Singapore, 5 Research Link, Singapore 117603, Republic of Singapore

[8] Department of Physics and Astronomy, University of British Columbia, Vancouver BC V6T 1Z1, Canada

[9] Institute for Quantum Science, School of Physical Science and Technology, Soochow University, Suzhou 215006, China

[#]These authors contributed equally to this work.

[*]Corresponding author:

slscst@nus.edu.sg (C.S.T.); sawatzky@physics.ubc.ca (G.A.S);

jiangmi@suda.edu.cn (M.J.); yinxinmao@shu.edu.cn (X.Y.)

Content

Supplementary Note 1. Sample Preparation ... 3

Supplementary Note 2. The 2θ-scan X-ray Diffraction Measurements and reciprocal space mappings ... 3

Supplementary Note 3. Various-angle XAS measurement ... 7

3.1. Experimental measurement ... 7

3.2. The observation of new feature A^* and fitting procedure in O K-edge XAS spectra ... 9

3.3. Analysis on Cu L-edge XAS spectra ... 13

Supplementary Note 4. Various-Temperature spectroscopy ellipsometry measurement ... 13

4.1. Experimental principle and methods ... 13

4.2. Various-Temperature spectroscopy ellipsometry data ... 16

4.3. The observation of new feature α^* and fitting procedure in σ_1 spectra ... 19

Supplementary Note 5. Oxygen vacancies in heavily overdoped LSCO ... 23

Supplementary Note 6. Electrical Resistivity Fitting model of heavily overdoped LSCO ... 24

Supplementary Note 7. Three-orbital Emery theoretic model ... 25

7.1. The determinant quantum Monte Carlo (DQMC) and maximum entropy analytic continuation (MaxEnt) method ... 25

7.2. Simulating three-orbital Emery model with determinant quantum Monte Carlo and maximum entropy analytic continuation method ... 27

7.3. Additional O feature peak above the Fermi level ... 28

7.4. Relationship with previous works and experimental results ... 30

Supplementary Reference ... 31

Supplementary Note 1. Sample Preparation

A series of heavily overdoped $La_{2-x}Sr_xCuO_4$ (LSCO, $0.15 \leq x \leq 0.6$) single crystals were grown on (001)-oriented $LaSrAlO_4$ (LSAO) single-crystal substrates using pulsed laser deposition (PLD). A KrF excimer laser operating at a repetition rate of 3 Hz and a pulse energy of 300 mJ was employed. The substrate temperature was maintained at 720 °C under an oxygen partial pressure of 20 Pa. After 6000 laser pulses, LSCO films with a thickness of approximately 90 nm were obtained. Post-deposition annealing was carried out in an oxygen atmosphere at 550 °C, with the oxygen pressure increased to 2000 Pa.

A uniform film thickness of ~90 nm was used for all LSCO samples in order to establish a consistent doping series and avoid introducing thickness as an additional variable. This thickness was chosen to ensure reliable spectroscopic and transport measurements while minimizing excessive substrate contributions. At the same time, it remains compatible with stable PLD growth and high epitaxial quality. Structural characterizations, including XRD, RSM, and STEM, confirm that films of this thickness are highly crystalline and coherently aligned with the substrate across the full doping range.

Supplementary Note 2. The 2*θ*-scan X-ray Diffraction Measurements and reciprocal space mappings

Synchrotron-based X-ray diffraction (XRD) measurements were performed on a series of hole doped $La_{2-x}Sr_xCuO_4$ (LSCO, $0.15 \leq x \leq 0.6$) single-crystalline films, as shown in Supplementary Figures 1–6. The XRD patterns reveal that all LSCO films exhibit a strong (00*l*) preferred orientation, consistent with the (00*l*) orientation of the LSAO substrates, indicating high epitaxial quality. Reciprocal space mappings (RSMs) around the $(004)_{HL}$ and $(004)_{KL}$ reflections for various overdoped LSCO films show that the LSCO peaks are directly aligned beneath those of the corresponding LSAO substrates, indicating fully coherent strain across the film–substrate interface [1]. Moreover, the RSMs of the $(-109)_{HL}$ and $(019)_{KL}$ reflections demonstrate the LSCO peaks exhibit equal absolute values in the *H* and *K* directions and identical positions along *L*, confirming the tetragonal symmetry in both LSCO films and LSAO substrates [1].

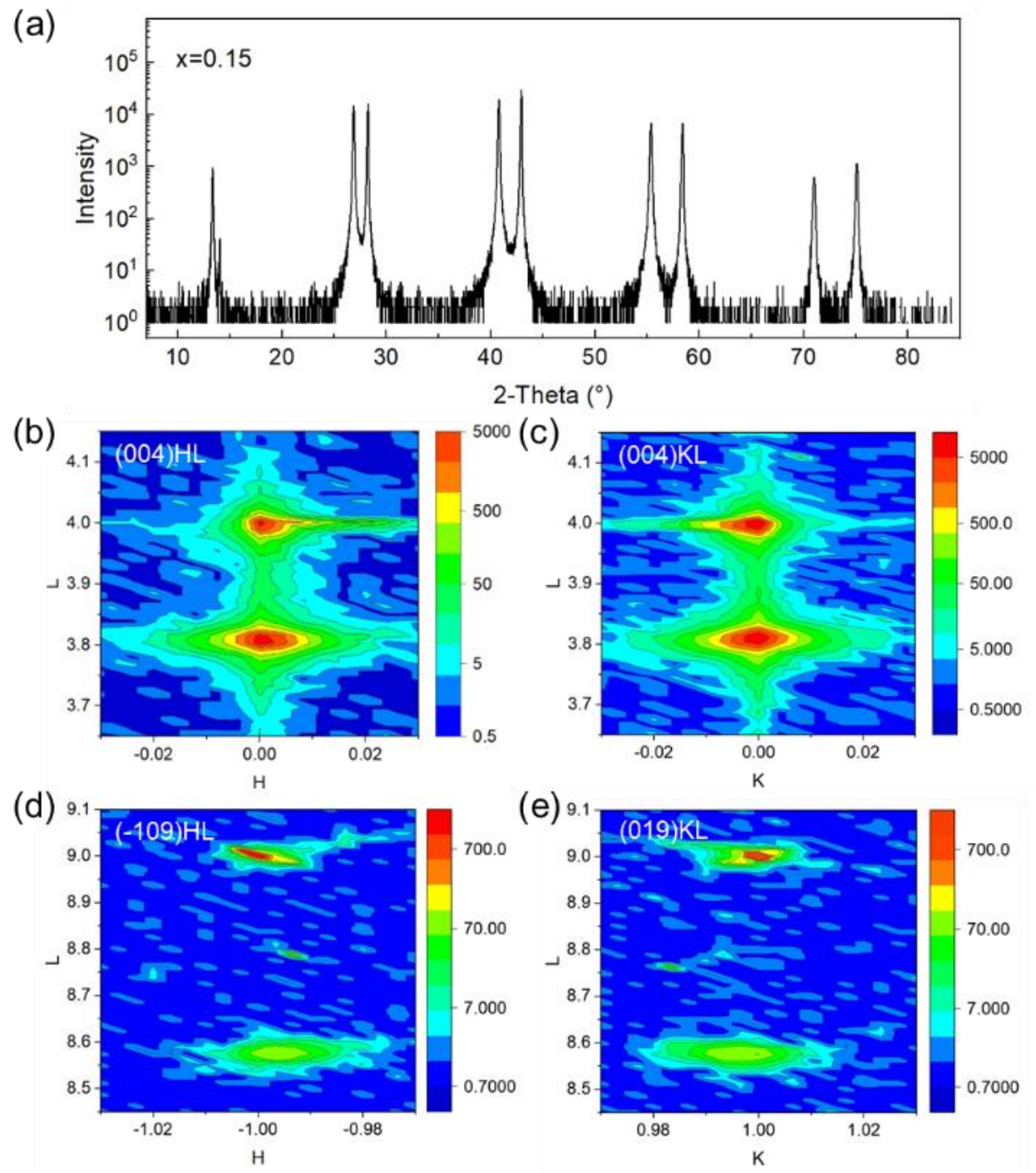

Supplementary Figure 1. The XRD results and RSMs for LSCO (x = 0.15) sample.

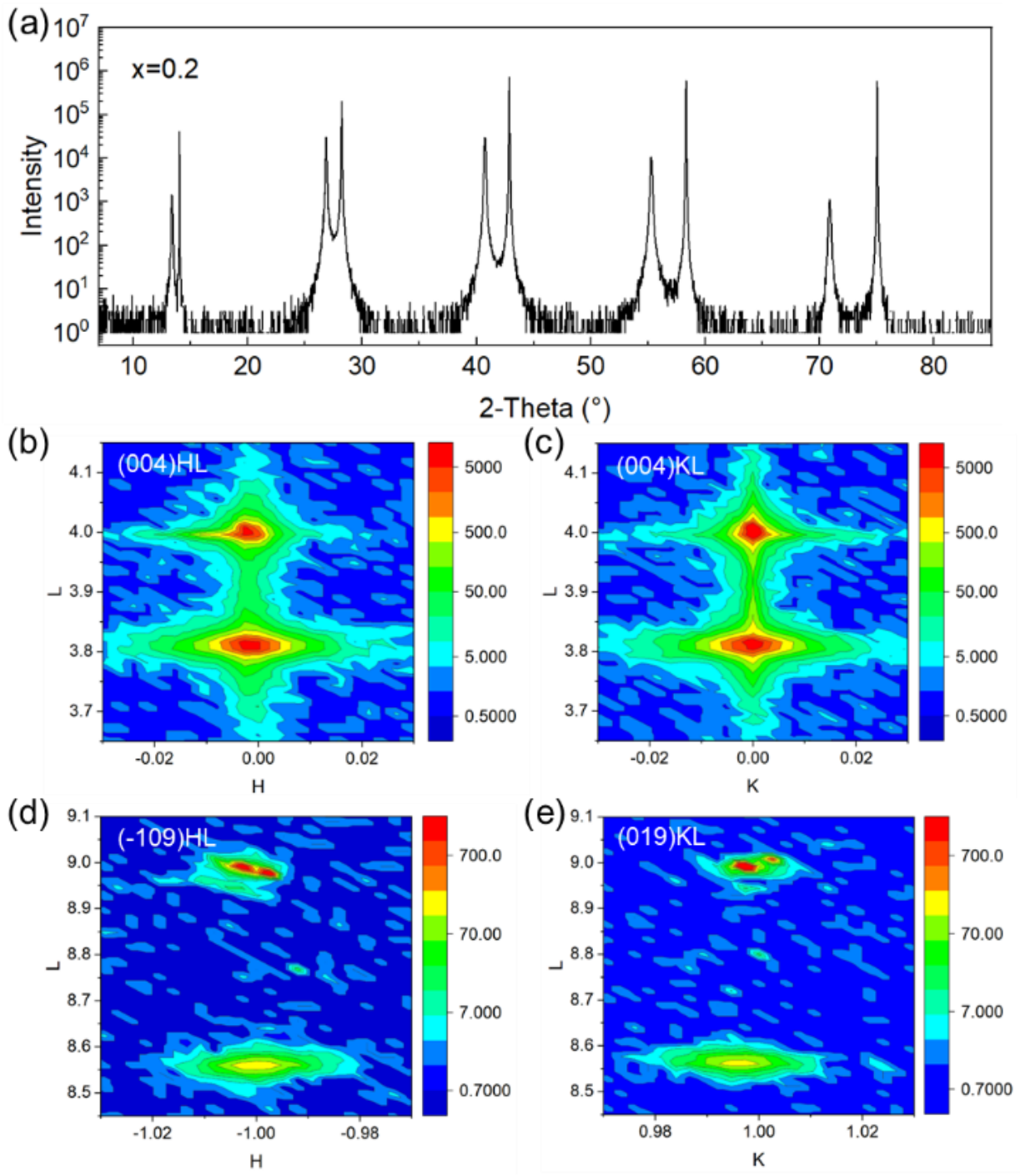

Supplementary Figure 2. The XRD results and RSMs for LSCO (x = 0.2) sample.

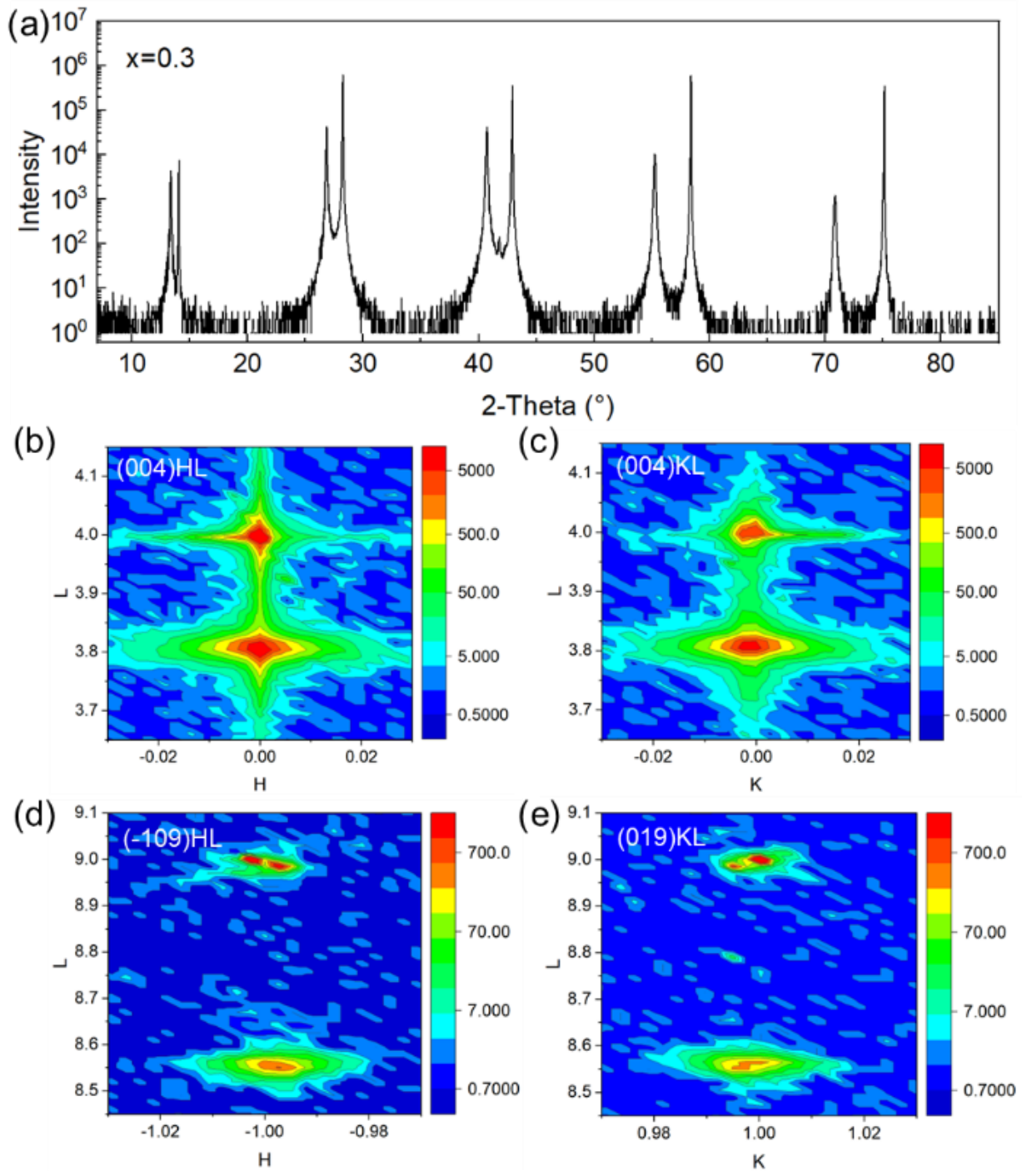

Supplementary Figure 3. The XRD results and RSMs for LSCO (x = 0.3) sample.

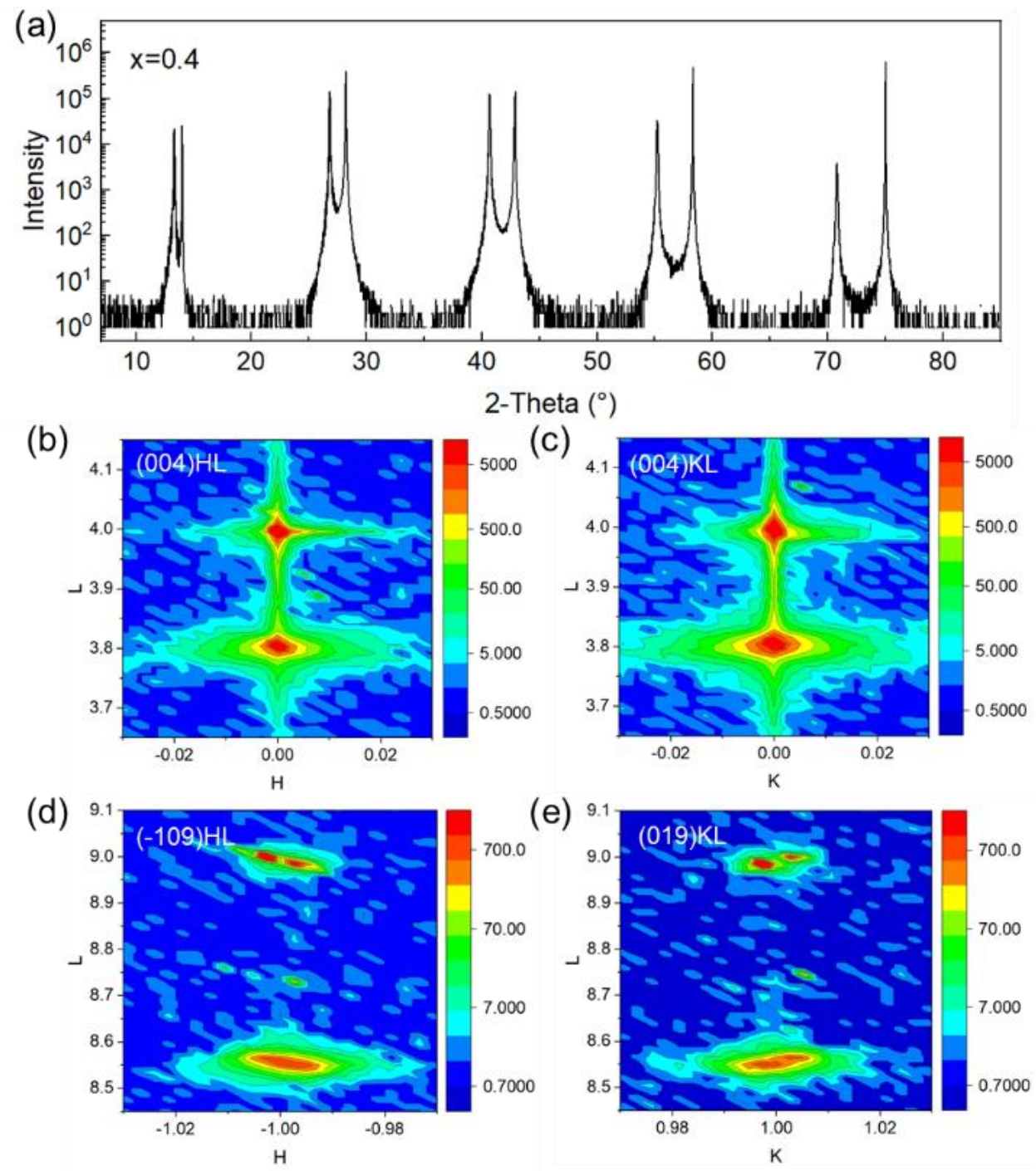

Supplementary Figure 4. The XRD results and RSMs for LSCO (x = 0.4) sample.

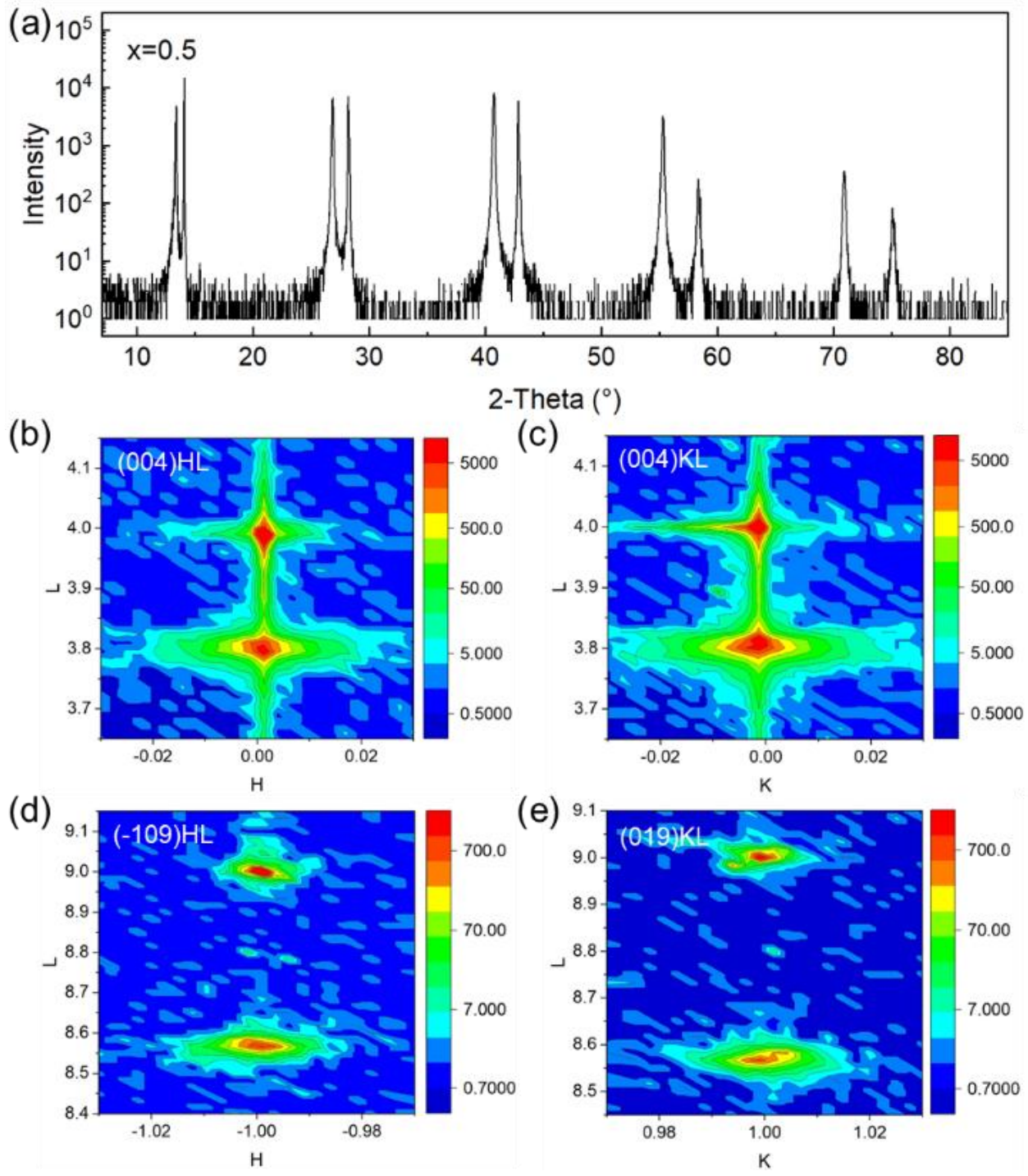

Supplementary Figure 5. The XRD results and RSMs for LSCO (x = 0.5) sample.

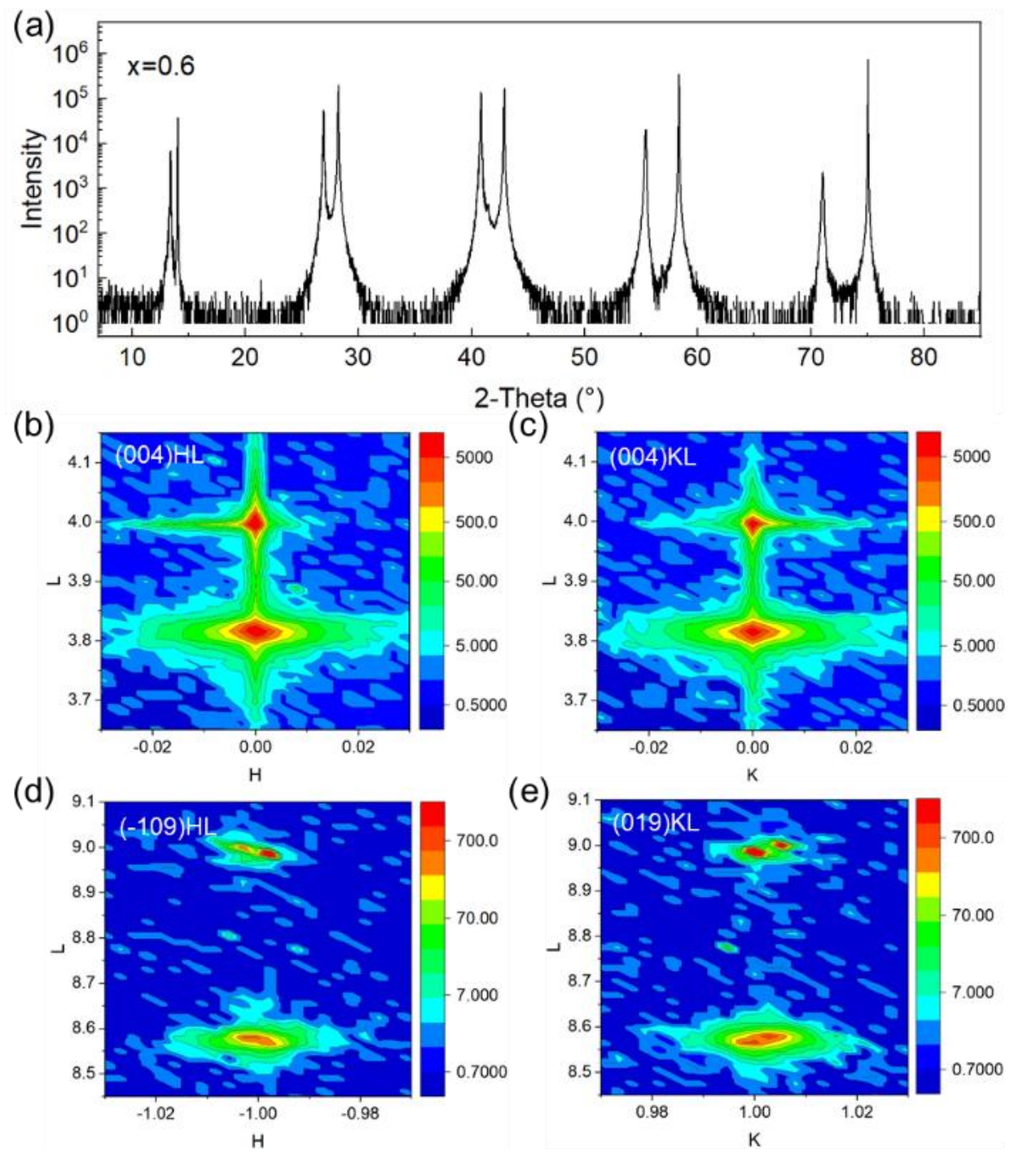

Supplementary Figure 6. The XRD results and RSMs for LSCO (x = 0.6) sample.

Supplementary Note 3. Various-angle XAS measurement

3.1. Experimental measurement

X-ray absorption spectroscopy (XAS) probes the local chemical environment around a photoabsorbing atom by monitoring unoccupied electronic states. It provides detailed insights into chemical bonding, oxidation and spin states, site symmetry, and coordination environments of specific atoms in systems such as biological materials, catalysts, nanoscale samples, electrochemical interfaces, geological specimens, and energy storage devices [2]. High-revolution XAS is particularly valuable for studying the change of electron structure within hole doped materials, thus, synchrotron-based various-angle X-ray absorption spectroscopy (XAS) was conducted on a series of overdoped LSCO ($0.15 \leq x \leq 0.6$).

The polarization-dependent O *K*-edge XAS spectra of LSCO ($0.15 \leq x \leq 0.6$), shown in Figure 2 and Supplementary Figure 7, were recorded at incident angles of 25°, 40°, and 90°, where the angle is defined between the incident X-ray beam and the sample surface (i.e., the horizontal plane). To gain insight into the electronic structure of LSCO, we compare the O *K*-edge XAS spectra acquired under different polarization conditions. It is worth mentioning that purely out-of-plane polarization (0°) spectra cannot be measured directly, as it would be impossible to obtain any absorption signal from the sample under strictly parallel conditions [3]. Therefore, it is necessary to orient the electric field vector E at an angle of 25° relative to the c-axis. As a result, our out-of-plane polarization spectra (25°) inevitably contain a finite contribution from the in-plane polarization response. Based on our estimations: For 90° polarization, the XAS spectrum only includes the in-plane component, while for 25° polarization, the xy-plane component should consist 82.1% out-of-plane component and 17.9% of in-plane component. The overall XAS intensity decreases as the polarization becomes more perpendicular to the CuO_2 planes. In the 25° and 40° spectra, four distinct features—labeled Z, A*, A, and B—are observed in the energy range of 527–531 eV. In contrast, only three features—A*, A, and B—are clearly discernible in the 90° spectrum.

Based on previously published studies, features A and B are consistently observed across all polarization conditions and are commonly attributed to transitions into the Zhang-Rice singlet (ZRS) and the Upper Hubbard Band (UHB), respectively [4, 5]. The additional feature Z, which appears exclusively in the 25° and 40° spectra but is absent in the 90° data, is interpreted as having *Z*-polarized character [6]. As the *Z*-component of the incident X-ray polarization increases (i.e., at lower incidence angles), the relative intensity of feature Z correspondingly increases, supporting its assignment to out-of-plane electronic states.

An intriguing and previously overlooked feature, labeled A*, emerges in the polarization-dependent O *K*-edge XAS spectra of LSCO ($0.15 \leq x \leq 0.6$). This inherent

spectral feature has not been reported in prior studies, likely due to resolution limitations. In contrast, our use of high-resolution synchrotron-based XAS enables the clear identification of this new feature A*.

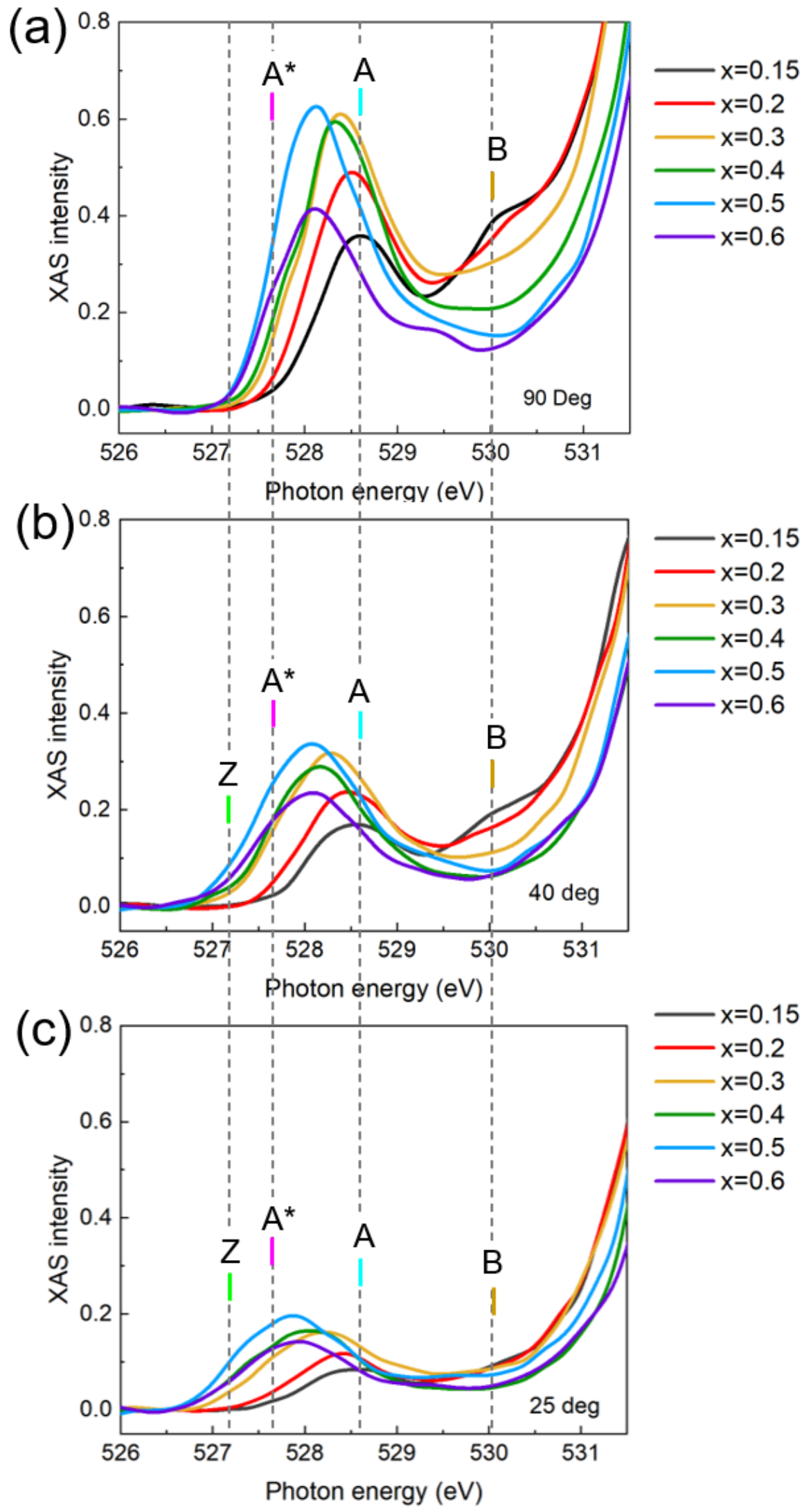

Supplementary Figure 7. The polarization-dependent O *K*-edge XAS spectra of LSCO ($0.15 \leq x \leq 0.6$) at incident angles of (a) 90°, (b) 40°, and (c) 25°.

The wide-range O *K*-edge and Cu *L*-edge XAS spectra measured at 90° incidence are presented in Supplementary Figure 8. An abrupt drop in XAS intensity is observed at the doping level of x = 0.6. One possible reason is the transfer of spectral weight to higher absorption energies [Supplementary Figure 8(a)]. In addition, the low-temperature resistivity curve of the x = 0.6 sample exhibits an insulating tendency [Figure 4(c)], suggesting that a local metal-to-insulator transition may also contribute to the pronounced suppression of XAS intensity at this doping level [Supplementary Figure 8(a), Figure 4(c)].

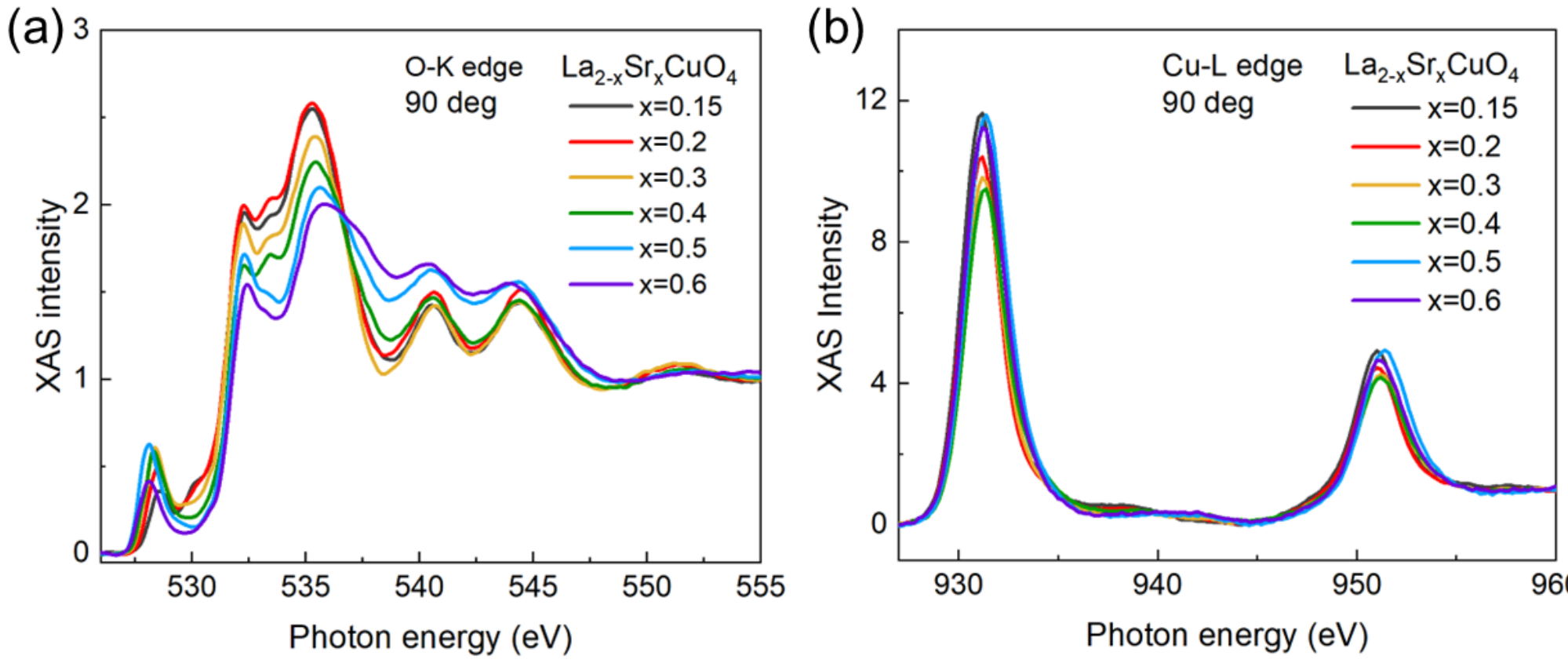

Supplementary Figure 8. The wide-range 90-deg O *K*-edge (526-555 eV) and Cu *L*-edge (927-960 eV) XAS spectra.

3.2. The observation of new feature *A** and fitting procedure in O *K*-edge XAS spectra

(1) The observation of new feature A*

A weak shoulder, denoted as feature A*, is observed in the XAS spectra at ~527.7 eV. The assignment of this feature A* is supported by several independent analyses. First, its energy position coincides with a curvature feature in the second-derivative XAS spectra (Supplementary Figures 9 and 10). This feature remains stable across heavily overdoped LSCO samples with $x \geq 0.3$, indicating that it is not an artifact of a particular dataset or a statistical fluctuation. Here, the second-derivative analysis is used only as a supplementary diagnostic to assist in identifying subtle spectral structure and is not relied upon as the sole basis for the peak assignment.

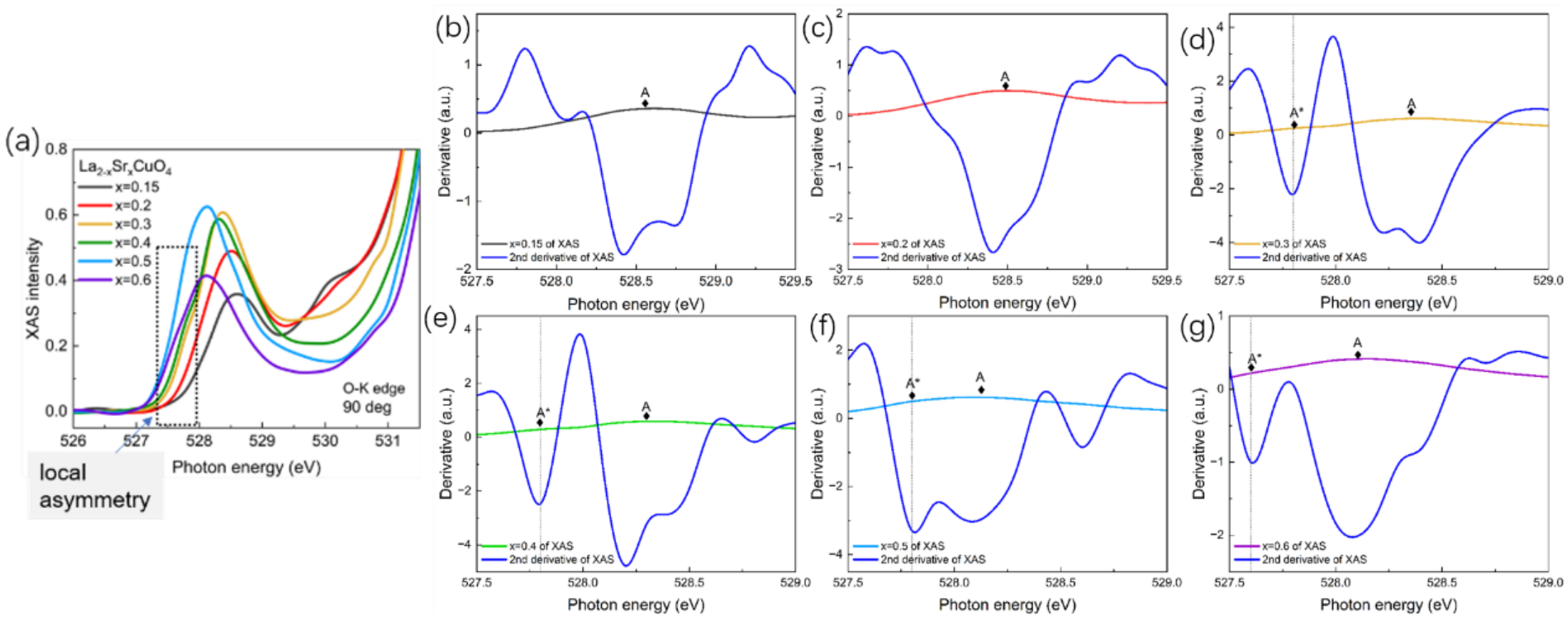

Supplementary Figure 9. The second derivative of the O *K*-edge XAS spectra measured at 90° incident angle.

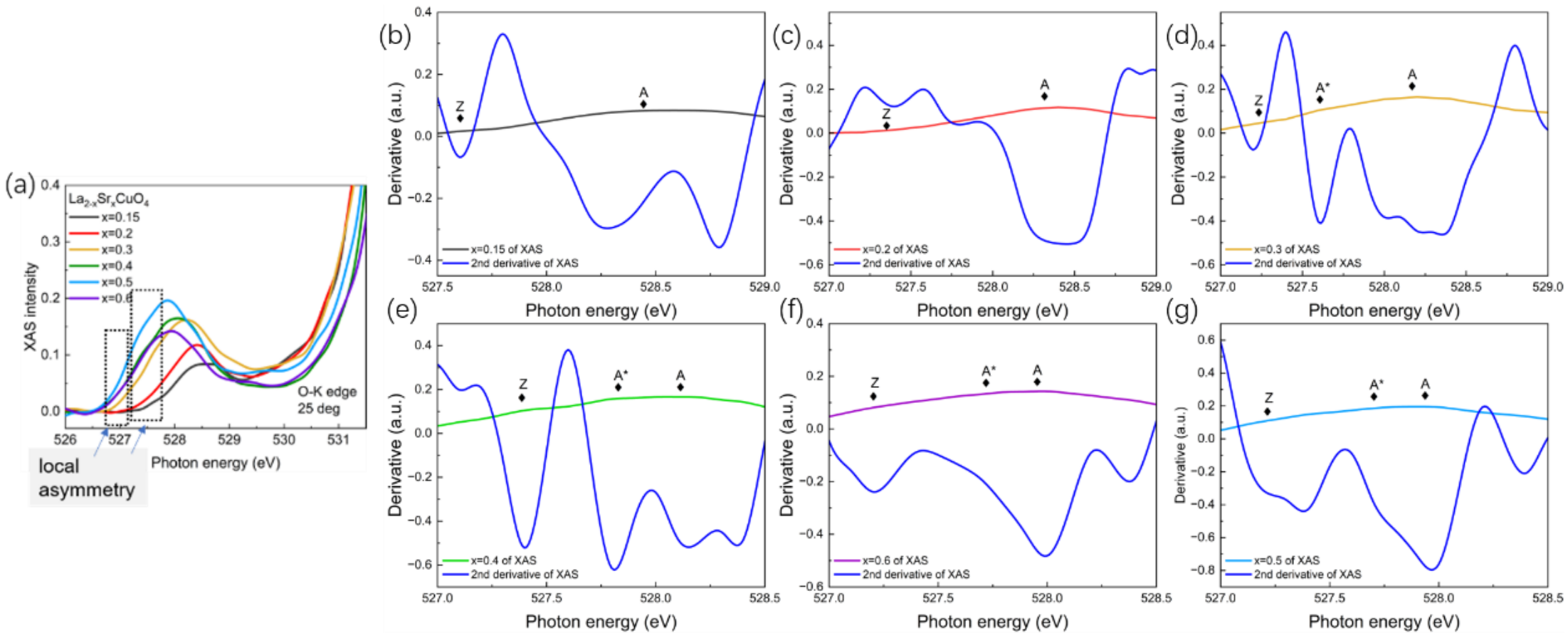

Supplementary Figure 10. The second derivative of the O *K*-edge XAS spectra measured at 25° incident angle.

In addition, differential XAS analysis was employed as a contrast-enhancement method to reveal weak spectral changes that are difficult to resolve directly in the raw XAS spectra (Supplementary Figure 11). The differential spectra show systematic line-shape variations associated with the emergence and evolution of features Z, A*, and A. We note, however, that the differential XAS response cannot by itself be interpreted as direct evidence for new spectral peaks, because it may also arise from shifts in peak position or changes in spectral intensity. Therefore, the differential XAS results were evaluated together with the local line shape of the original spectra and the outcomes of the fitting analysis. Taken together, the consistency of the second-derivative feature, the systematic behavior observed in the differential XAS spectra, the direct shoulder-like structure in the raw spectra, and the quantitative fitting results collectively support the interpretation that the shoulder near 527.7 eV (feature A*) represents a genuine spectral component rather than an artifact of the analysis procedure.

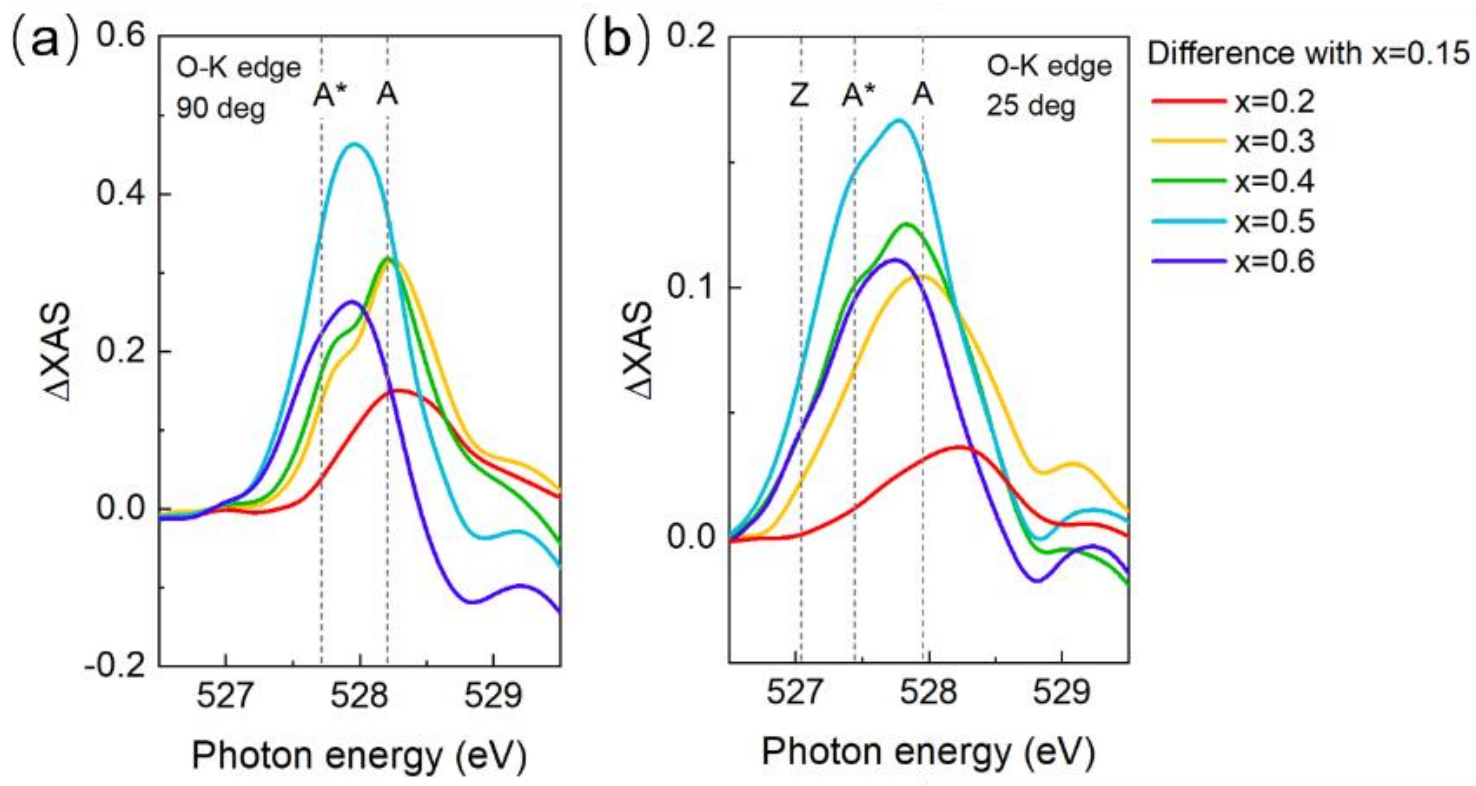

Supplementary Figure 11. The differential XAS spectra ΔXAS measured at 90° and 25° incident angles.

(2) Fitting procedure

To quantitatively characterize the doping evolution of Feature A*, the O K-edge pre-edge spectra were analyzed using a consistent fitting procedure across the entire doping series. The fitting results for the 90° geometry are presented in Figure 2(e) of the main text, while the corresponding analysis for the 25° geometry is shown in Supplementary Figure 12. Comparison of the 90° and 25° O *K*-edge XAS spectra shows that, although the absolute intensities of both A* and A decrease as the out-of-plane contribution increases, the relative intensity of A* with respect to A becomes enhanced at 25°, suggesting that A* retains predominantly in-plane character but acquires a comparatively stronger out-of-plane orbital admixture in the heavily doped regime, possibly due to increased occupation of, or hybridization with, out-of-plane orbitals by doped holes [6].

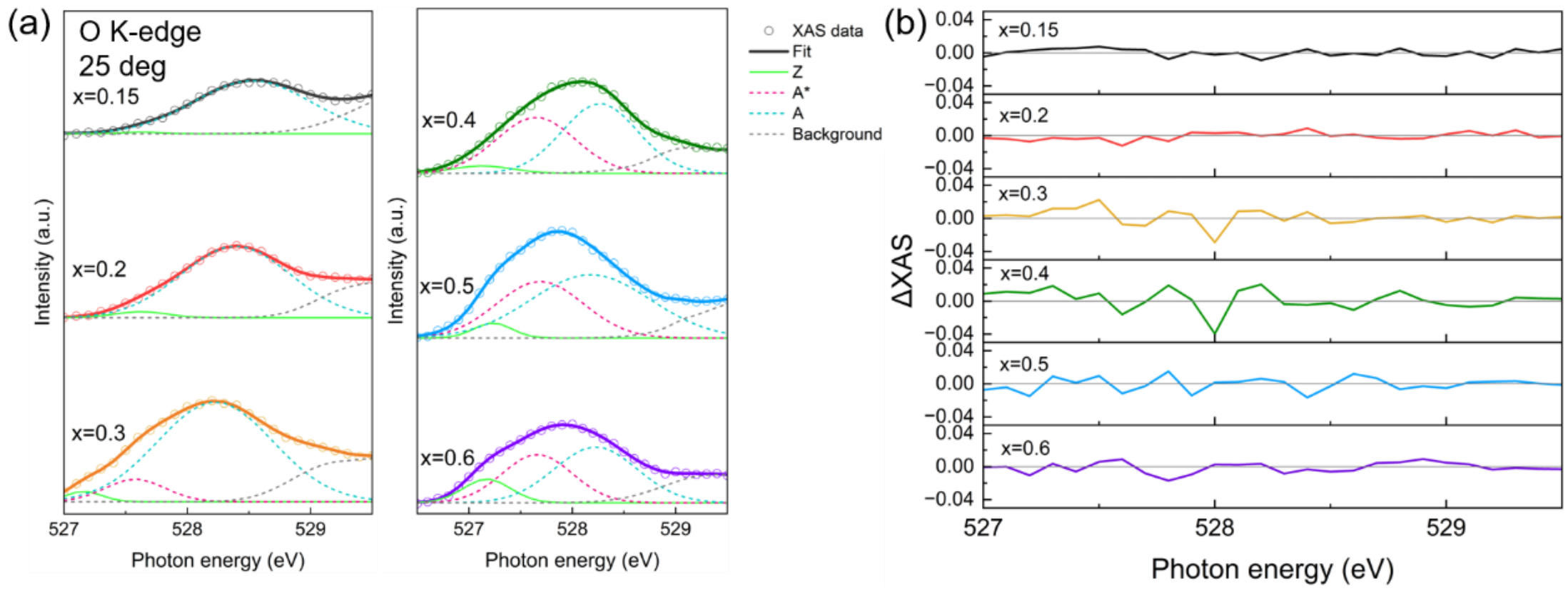

Supplementary Figure 12. (a) Gaussian fitting results of the O *K*-edge spectra measured at 25°. (b) Residual spectra, $\Delta XAS = I_{\text{exp}} - I_{\text{fit}}$, for the 90° O *K*-edge spectra.

The XAS spectra were fitted using the Gaussian model. The purpose of this Gaussian-based parameterization is not to identify a unique microscopic broadening mechanism for each spectral feature, but rather to provide a stable and internally consistent quantitative description of the doping-dependent pre-edge spectral evolution. Relatively narrow Gaussian components were used to parameterize the experimentally resolved Features Z, A*, and A. In addition, several broad Gaussian components were introduced to describe the slowly varying high-energy spectral contribution extending into the selected fitting window. These broad components are treated collectively as an effective spectral background and are not assigned to additional discrete electronic excitations. The same fitting protocol was applied consistently across the entire doping series. The resulting center energies, FWHMs, integrated spectral weights, and goodness-of-fit metrics are summarized in Supplementary Tables 1 and 2.

Supplementary Table 1. The fitting parameters of features A and A* in the O K-edge 90 deg XAS spectra obtained from fitting analysis.

LSCO sample O-K edge 90 deg	Feature A*			Feature A			R^2	RMSE
	Center energy (eV)	FWHM (eV)	Integrated intensity (eV)	Center energy (eV)	FWHM (eV)	Integrated intensity (eV)		
X=0.15	/	/	/	528.57	0.99	0.36	0.9993	0.004347
X=0.2	/	/	/	528.48	0.98	0.49	0.9990	0.004555
X=0.3	527.75	0.29	0.028	528.37	0.83	0.51	0.9961	0.009238
X=0.4	527.71	0.41	0.049	528.33	0.81	0.48	0.9956	0.01231
X=0.5	527.85	0.53	0.065	528.14	0.95	0.55	0.9980	0.007994
X=0.6	527.72	0.58	0.043	528.17	0.97	0.38	0.9975	0.006434

Supplementary Table 2 The fitting parameters of features A, A* and Z in the O K-edge 25 deg XAS spectra obtained from fitting analysis.

LSCO sample O-K edge 25 deg	Feature Z			Feature A*			Feature A			R^2	RMSE
	Center energy (eV)	FWHM (eV)	Integrated intensity (eV)	Center energy (eV)	FWHM (eV)	Integrated intensity (eV)	Center energy (eV)	FWHM (eV)	Integrated intensity (eV)		
X=0.15	527.63	0.33	0.00095	/	/	/	528.53	1.11	0.10	0.9992	0.001735
X=0.2	527.62	0.50	0.0052	/	/	/	528.39	1.04	0.13	0.9995	0.000960
X=0.3	527.16	0.34	0.0061	527.58	0.57	0.022	528.23	1.10	0.19	0.9993	0.001347
X=0.4	527.13	0.69	0.0099	527.65	0.90	0.098	528.27	0.86	0.12	0.9967	0.002365
X=0.5	527.23	0.43	0.013	527.69	0.99	0.108	528.18	1.36	0.17	0.9985	0.001869

X=0.6	527.17	0.54	0.025	527.66	0.80	0.075	528.23	0.94	0.10	0.9991	0.001364

3.3. Analysis on Cu *L*-edge XAS spectra

The polarization-dependent XAS spectra at the Cu L_3 edge display features C (~931.2 eV) and D (~932.6 eV) across all doping levels of LSCO, as shown in Supplementary Figure 13. Feature C is attributed to the transition from the $2p^63d^9$ to $2p^53d^{10}$ state, while feature D arises from the transition from $2p^63d^9L$ to $2p^5d^{10}L$ [7, 8]. In addition, the shoulder feature D represents a signature of the ZRS state whose spectral weight is proportional to the number of holes introduced to the CuO_2 planes [9, 10]. Supplementary Figure 13(d) plots the intensity evolution of feature D at the Cu L_3 edge, which exhibits a systematic increase in XAS intensity with increasing doping.

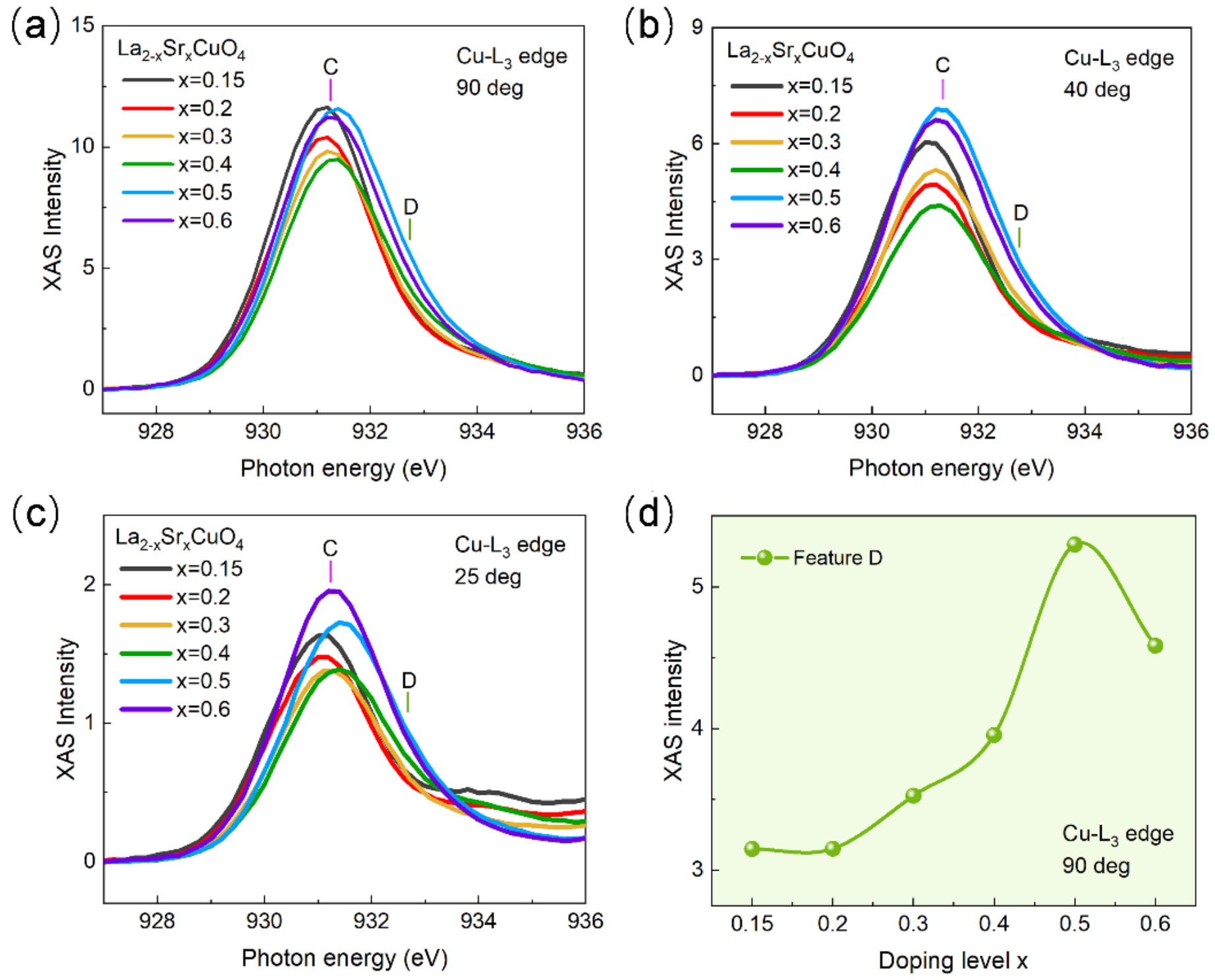

Supplementary Figure 13 (a) 90 deg, (b) 40 deg, (c) 25 deg XAS spectra at the Cu L_3-edge across all doping levels of LSCO. (d) The intensity evolution of feature D.

Supplementary Note 4. Various-Temperature spectroscopy ellipsometry measurement

4.1. Experimental principle and methods

High-resolution spectroscopic ellipsometry (SE) provides broad optical energy coverage and enables temperature- and angle-dependent measurements, making it a powerful tool for identifying and investigating quasiparticle excitations in a wide range

of materialsv [11]. Variable-temperature spectroscopic ellipsometry (SE) measurements were performed using a custom-built J.A. Woollam Co., Inc. Variable Angle Spectroscopic Ellipsometer (VASE) over a photon energy range of 0.5–3.5 eV, with an optimal incident angle of 70° relative to the sample normal. The measurements were carried out in a high-vacuum chamber (~10^{-9} mTorr) across a temperature range of 5 K to 300 K, encompassing both the superconducting and normal states. This wide temperature range allows for the investigation of changes in the electronic structure across the superconducting phase transition. The Schematic of spectroscopic ellipsometry is drawn in Supplementary Figure 14.

SE measurements can directly measure two ellipsometric parameters: the Ψ: amplitude ratio between p- and s- polarized reflected light, and the Δ: phase difference between of *p*- and *s*-polarized reflected light. Subsequently, using the ellipsometric parameters Ψ and Δ can obtain the dielectric coefficients (ε_1 and ε_2) and optical conductivity (σ_1) of the LSCO/LSAO films through the air/LSCO/LSAO multilayer model, where the LSCO film is treated as a homogeneous and uniform medium on average.

Ψ and Δ are signed as

$$\tan\Psi\ exp(i\Delta) \equiv \frac{r_p}{r_s} \tag{S1}$$

Where r_p and r_s denote the reflectivity coefficients for *p*- and *s*-polarized light, respectively. Based on the Fresnel equations, they can be expressed as

$$r_p^{ij} = \frac{n_j\cos\theta_i - n_i\cos\theta_j}{n_j\cos\theta_i + n_i\cos\theta_j} \tag{S2}$$

and

$$r_s^{ij} = \frac{n_j\cos\theta_i - n_i\cos\theta_j}{n_j\cos\theta_i + n_i\cos\theta_j} \tag{S3}$$

Where n and θ represent the refractive index and the angle of incidence relative to the surface normal, respectively, and i and j denote two different materials. The complex dielectric function of media $\varepsilon(\omega) = \varepsilon_1(\omega) + i\varepsilon_2(\omega)$ can be determined by

$$N^2 \equiv \varepsilon \tag{S3}$$

where ω denotes the photon frequency.

For a typical two-layer thin-film system, such as a LSCO film on a LSAO substrate, the reflectivity can be expressed as:

$$r_{multi} = \frac{r_{vac,film} + r_{film,sub} exp\left(i2\delta_{film}\right)}{1 + r_{vac,film} \cdot r_{film,sub} exp\left(i2\delta_{film}\right)} \tag{S5}$$

where

$$\boldsymbol{\delta_{film}} = \frac{\boldsymbol{2\pi d_{film}}}{\boldsymbol{\lambda}} \boldsymbol{n_{film}} \cos \boldsymbol{\theta} \tag{S6}$$

where the subscripts *multi* and *vac* refer to the LSCO/LSAO multilayer system and vacuum, respectively. δ_{film} represents the phase shift of light upon reflection from the LSCO film, λ is the wavelength of the incident light, and d_{film} is the thickness of the LSCO layer deposited by PLD. The refractive index of the LSAO substrate was obtained from spectroscopic ellipsometry measurements on a clean bulk LSAO sample.

After determining the complex dielectric function of the LSAO substrate, the dielectric function of the LSCO films can be extracted from the measured Ψ and Δ by fitting the data using a point-by-point model. Then, the characteristic peaks can be fitted using the Drude–Lorentz oscillator model, according to:

$$\tilde{\varepsilon}(\omega) = \varepsilon_\infty + \sum_{k=1}^{n} \frac{{\omega_{pk}}^2}{{\omega_{0k}}^2 - \omega^2 - i\gamma_k\omega} \tag{S7}$$

$$\tilde{\varepsilon}(\omega) = \varepsilon_1(\omega) + i\varepsilon_2(\omega) \tag{S8}$$

where ω_0 is the oscillator frequency parameter, ω_p is the plasma frequency parameter, γ is the line-width parameter. ε_∞ represents the contribution from higher-frequency electronic transitions not captured by the modeled oscillators.

Subsequently, the real part of the optical conductivity σ_1, can be calculated using the following equation [12]:

$$\sigma_1 = \omega \cdot \varepsilon_0 \cdot \varepsilon_2 = \frac{E}{\hbar} \cdot \varepsilon_0 \cdot \varepsilon_2 = \frac{2\pi\varepsilon_0}{h} \cdot E \cdot \varepsilon_2 \tag{S9}$$

where ω is the frequency of photon, ε_0 is a constant which is approximately equal to 8.85×10^{-12} F/M, ε_2 is the imaginary part of the dielectric function, E is the energy of photon, and $\hbar$ is the reduced Planck constant, with a unit-converted value of approximately 4.14×10^{-15} eV·s.

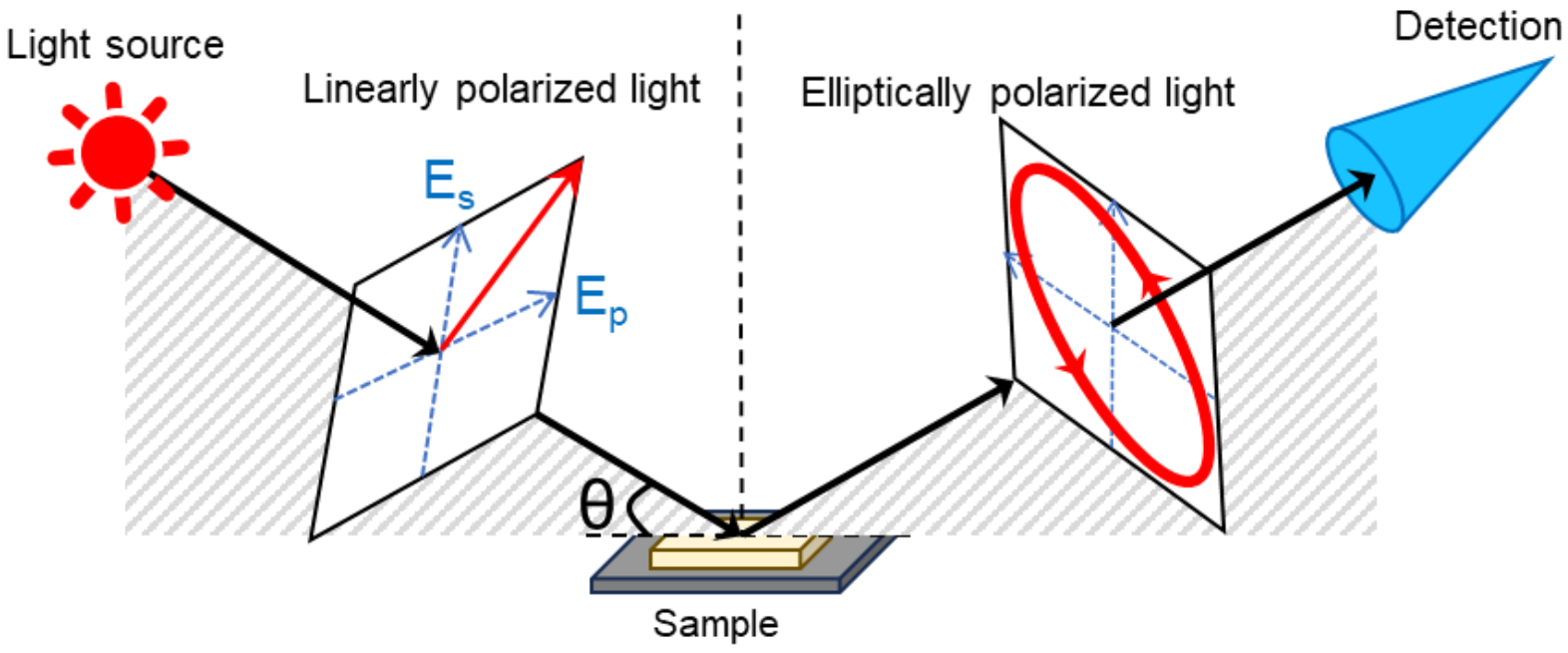

Supplementary Figure 14. The Schematic of experimental principle and methods in spectroscopic ellipsometry

The entire optical conductivity σ_1 spectrum is obtained from spectroscopic ellipsometry (0.5-3.5 eV) and linear extrapolation of electron conductivity (0-0.5 eV), as shown in Supplementary Figure 15(a). Detailed analysis of optical conductivity spectrum in main text. In addition, the spectral weight (SW) is plotted by integrating the σ_1 via the formula $SW = \int_{\omega 2}^{\omega 1} \sigma_1(\omega')d\omega'$ and the results in Supplementary Figure 15(b).

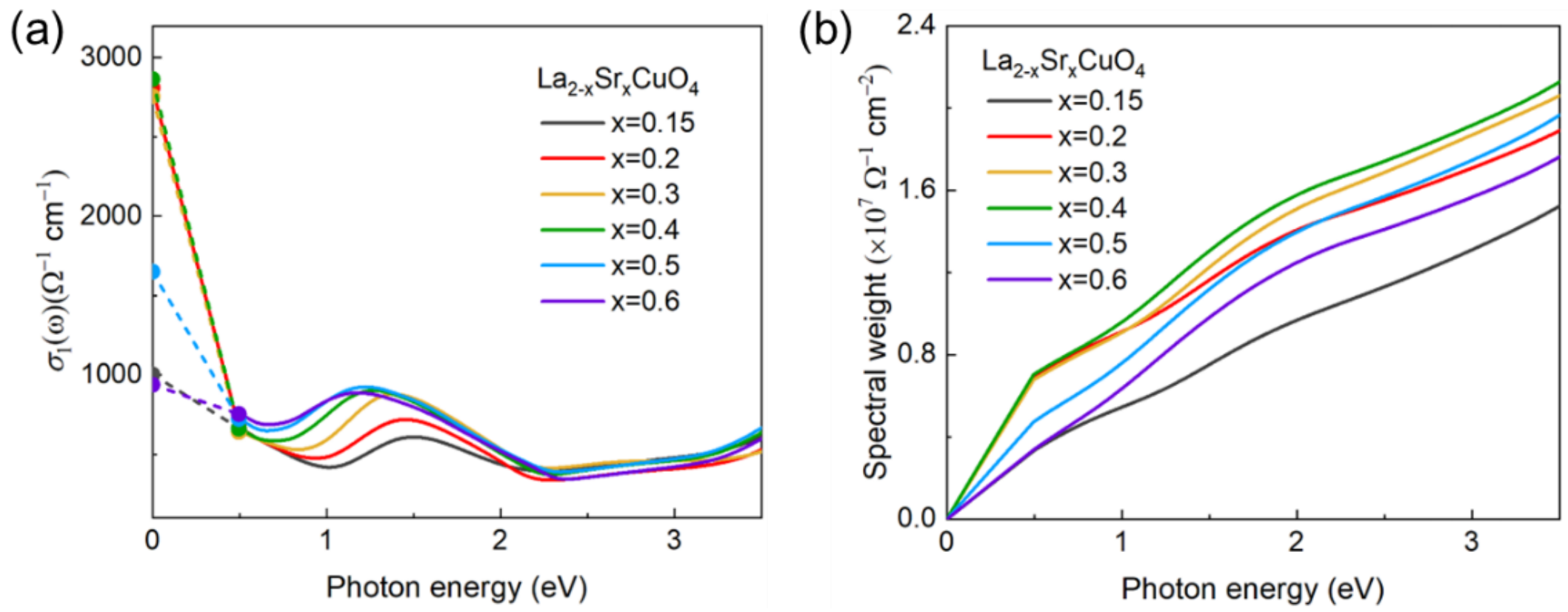

Supplementary Figure 15. (a) Optical conductivity σ_1 and spectral weight (SW) spectra of the LSCO samples with different doping concentration.

4.2. Various-Temperature spectroscopy ellipsometry data

Optical spectroscopic ellipsometry represents a highly versatile tool for probing optical response and band transitions and effectively unweaving the electronic structure in strongly correlated materials at the nanoscale. Thus, OSE be utilized to study the evolving electronic structures and superconductive properties of LSCO sample with different doping levels. The Various-Temperature spectroscopy ellipsometry data of temperature from 5 K to 300 K and photon energy from 0.5 eV to 3.5 eV is present in

Supplementary Figures 16-17, including the real $\varepsilon_1(\omega)$ and imaginary $\varepsilon_2(\omega)$ parts of the dielectric function and optical conductivity σ_1 spectra.

Supplementary Figures 16-17 are various-temperature OSE data of a series of LSCO ($0.15 \leq x \leq 0.6$) samples. Our OSE experimental results show similar ε_1, ε_2, and σ_1 among the different temperature points acrossing the superconducting transition temperature. The zero-crossing point in the ε_1 spectra and high-intensity Drude feature in ε_2 spectra indicate the metal property for all temperatures [13]. while the feature at ~1.50 eV slightly redshifts as the temperature decreasing, indicating the energy position of band shifts as the temperature changing.

Notably, a feature centered around ~1.50 eV in σ_1 spectra exhibits a slight redshift upon cooling, suggesting a temperature-dependent shift in the underlying band structure (Supplementary Figure 17). Simultaneously, the σ_1 intensity in the 1–2 eV energy range gradually increases with increasing doping level, which is attributed to the emergence of a new feature, labeled α^*, near 1.1 eV (discussed in detail in the main text). As doping increases from optimal side to heavily overdoped side, the superconducting transition temperature T_c decreases and the effect of disorder becomes more pronounced, causing more spectral weight transfer among the finite-frequency spectrum [14]. In addition, the fluctuation in σ_1 becomes more pronounced at lower temperatures.

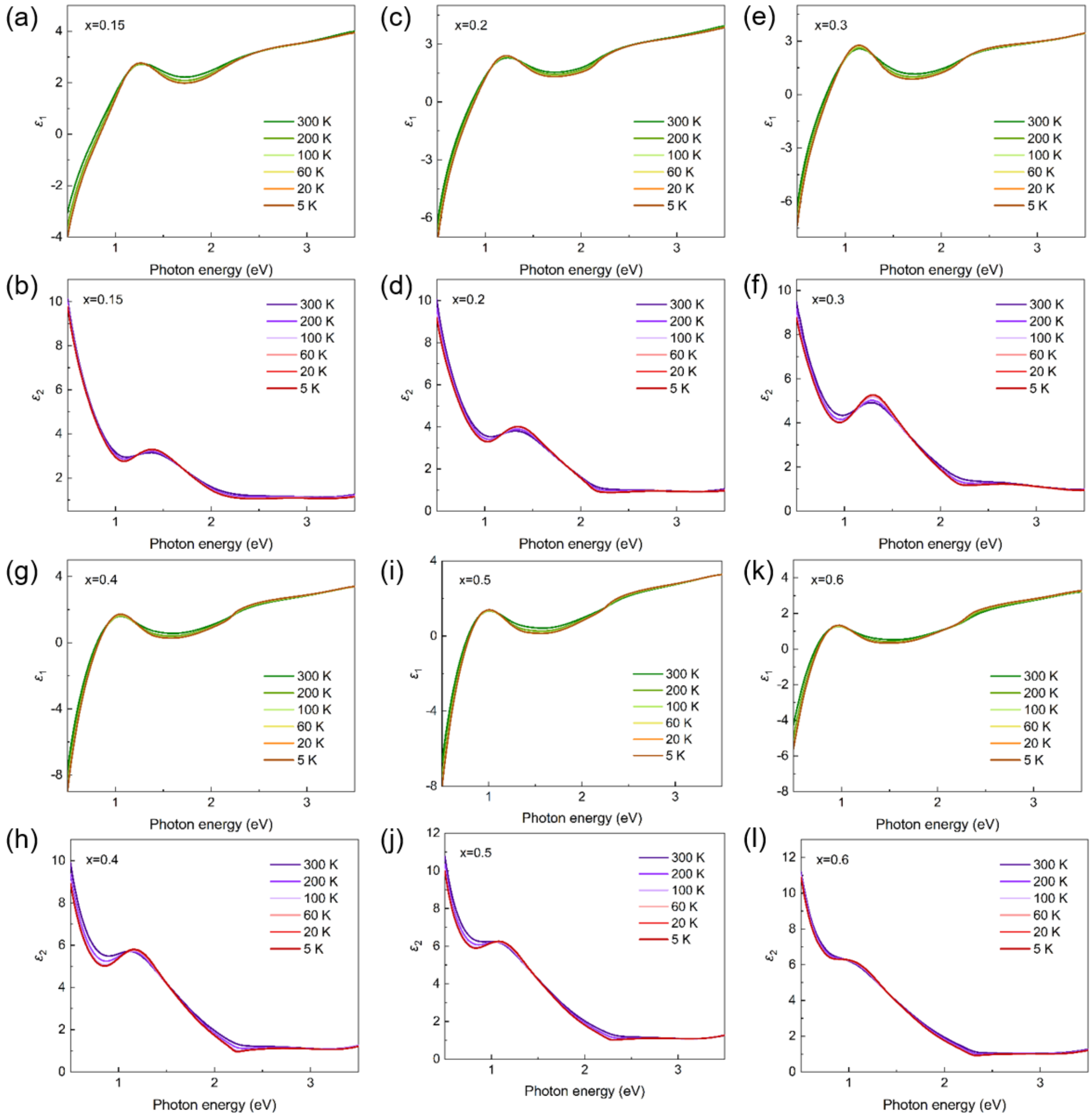

Supplementary Figure 16. The real $\varepsilon_1(\omega)$ and imaginary $\varepsilon_2(\omega)$ parts of the dielectric function for LSCO samples with different doping concentration ($0.15 \leq x \leq 0.6$).

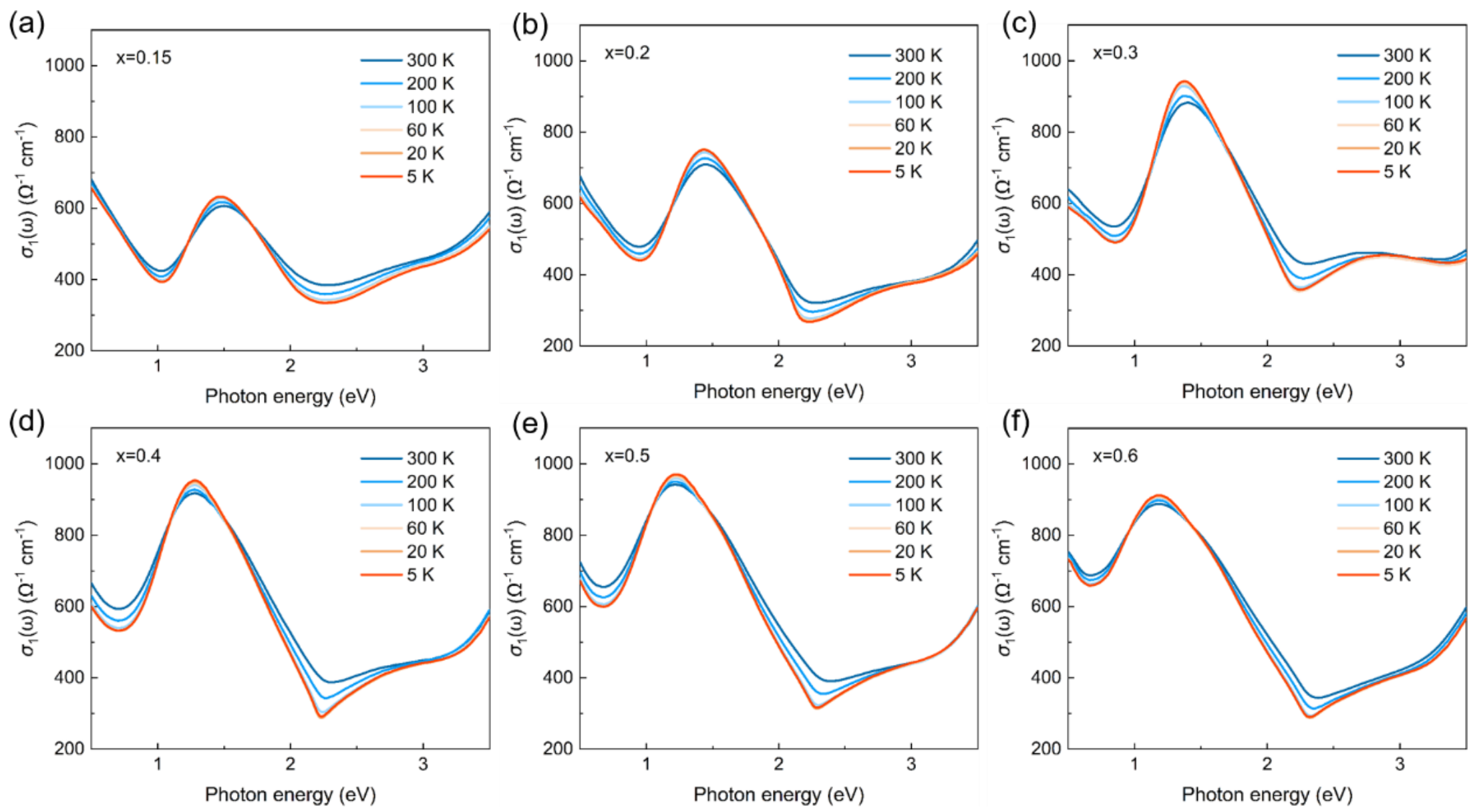

Supplementary Figure 17. The optical conductivity σ_1 spectra for LSCO samples with different doping concentration (0.15≤x≤0.6).

4.3. The observation of new feature *α** and fitting procedure in σ_1 spectra

(1) The observation of new feature *α**

The differential optical conductivity spectra, $\Delta\sigma_1$, provide a more sensitive means of identifying subtle spectral features and estimating their approximate energy positions. Two distinct spectral signatures associated with features α and α* are observed in the $\Delta\sigma_1$ spectra (Supplementary Figure 18(a)), providing independent evidence for the presence of these features in the optical conductivity, σ_1, spectrum.

On this basis, a detailed fitting analysis of the optical conductivity spectra was subsequently performed in order to determine the feature energies more accurately. The fitting results are plotted in Figure 3(d) in the main text.

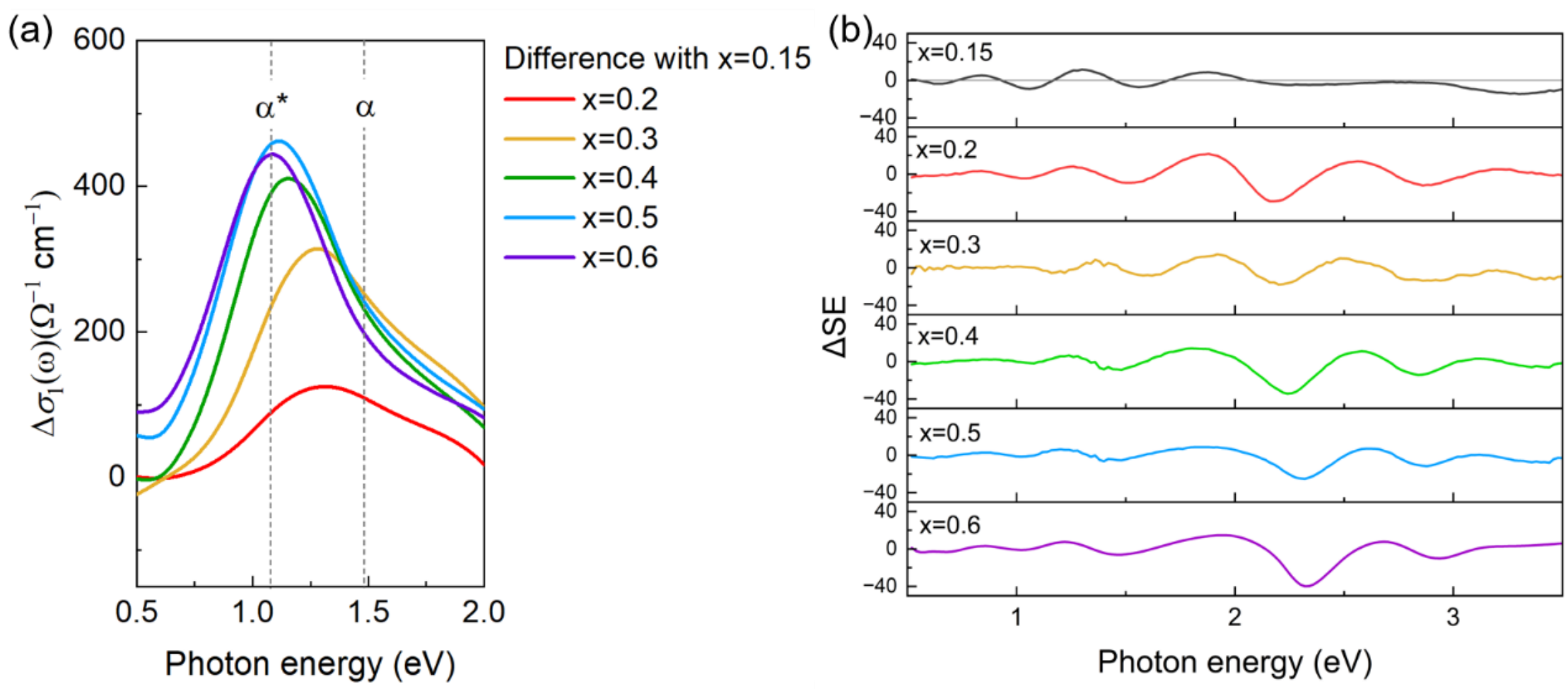

Supplementary Figure 18. (a)The differential optical conductivity $\Delta\sigma_1$ spectra from

optical spectroscopic ellipsometry measurements. (b) Residual spectra, $\Delta SE = \sigma_{1,\mathrm{exp}} - \sigma_{1,\mathrm{fit}}$, for x = 0.15-0.6.

Through this combined analysis of $\Delta\sigma_1$ and the fitted σ_1 spectra, the energies of features α and α* were extracted to be ~1.5 eV and ~1.1 eV, respectively. The appearance of the α^* feature provides further evidence for the emergence of a new low-energy excitation channel in the heavily overdoped regime and supports the conclusion of doping-induced electronic-structure reconstruction in LSCO. Using this systematic fitting procedure, the energy positions of features α and α* were extracted for all doping concentrations and are summarized in Supplementary Table 3.

Supplementary Table 3. The fitting parameters of Drude-Lorentz in the optical conductivity spectra obtained from fitting analysis.

		Amplitude (eV)	Broadening (eV)	Energy (eV)	R^2
X=0.15	Drude	A_D = 4.299 eV^2	B_D = 0.452 eV		0.9970
	Lorentz	1.440	0.523	0.672	
	Feature α	2.121	0.899	1.531	
	Feature β	0.401	1.473	2.774	
X=0.2	Drude	A_D = 4.532 eV^2	B_D = 0.301 eV		0.9950
	Lorentz	1.759	0.569	0.671	
	Feature α	2.921	0.981	1.491	
	Feature β	0.298	0.708	2.860	
X=0.3	Drude	A_D = 4.185 eV^2	B_D = 0.218 eV		0.9962
	Lorentz	2.725	0.729	0.650	
	Feature α*	1.394	0.534	1.253	
	Feature α	2.811	1.008	1.554	

	Feature β	0.455	0.940	2.828	
X=0.4	Drude	A_D = 3.990 eV^2	B_D = 0.148 eV		0.9968
	Lorentz	4.523	0.950	0.508	
	Feature α*	1.262	0.551	1.128	
	Feature α	3.181	1.129	1.445	
	Feature β	0.272	0.587	2.834	
X=0.5	Drude	A_D = 4.481 eV^2	B_D = 0.229 eV		0.9984
	Lorentz	2.407	1.027	0.642	
	Feature α*	2.098	0.670	1.105	
	Feature α	2.702	1.192	1.490	
	Feature β	0.208	0.541	2.860	
X=0.6	Drude	A_D = 4.487 eV^2	B_D = 0.343 eV		0.9967
	Lorentz	1.62	0.899	0.719	
	Feature α*	2.201	0.674	1.079	
	Feature α	2.411	1.178	1.512	
	Feature β	0.178	0.521	2.919	

For the Drude term, $A_D=(\hbar\omega_{p,D})^2$, $B_D=\hbar\gamma_D$

(2) Drude-Lorentz Model

The dielectric functions of metals and semiconductors with high carrier concentrations exhibit strong free carrier absorption characteristics, which can be represented by the Drude model. The carrier concentration and mobility in metals/semiconductors can be estimated using the Drude model for analysis. However, the measured dielectric response contains contributions from both free carriers and bound electronic excitations. Therefore, the dielectric function of a metal/semiconductor is expressed as

$$\tilde{\varepsilon}(\omega) = \varepsilon_1(\omega) + \varepsilon_2(\omega) \quad \text{(S10)}$$

$$\tilde{\varepsilon}(\omega) = \tilde{\varepsilon}_{free}(\omega) + \tilde{\varepsilon}_{bound}(\omega) \quad \text{(S11)}$$

The free electron component as represented by $\tilde{\varepsilon}_{free}$ is modelled using the Drude model while the bound-electrons component represented by $\tilde{\varepsilon}_{bound}$ is modelled using Lorentz oscillators. Together, these terms provide a physically motivated description of the dielectric response and enable quantitative modeling of the optical spectra.

The Drude–Lorentz model has been extensively applied to semiconducting and

correlated-electron systems that contain both free-carrier and bound-electron contribution. Its flexibility and physical basis make it particularly suitable for describing dielectric functions over a broad spectral range and for extracting characteristic optical excitations through curve fitting [13, 15].

(3) Fitting procedure

The optical conductivity spectra were analyzed using a consistent Drude–Lorentz model across the entire doping series. No independent empirical baseline was subtracted prior to fitting; instead, the measured electrodynamic response was described directly by the Drude and Lorentz contributions.

The dominant free-carrier response was represented by a Drude term. We found, however, that a single Drude contribution does not fully reproduce the spectral curvature near the low-energy limit of the measured range. Therefore, an additional low-energy Lorentz oscillator was included for all doping concentrations to account phenomenologically for the residual finite-frequency response not captured by the ideal Drude term. The resonance energy of this low-energy oscillator lies at approximately 0.5–0.7 eV, well below the characteristic energies of Features α^* and α. Its contribution is therefore treated as a correction to the low-energy electrodynamic response and is not assigned to either α^*or α.

Accordingly, the spectra for x = 0.15 and 0.2 were described using one Drude term, the additional low-energy Lorentz oscillator, the Lorentz components associated with Features α ,β .and high energy contribution. For $x \geq 0.3$, an additional Lorentz oscillator associated with Feature α^* was required to reproduce the experimentally observed redistribution of spectral weight near 1.1 eV. This fitting strategy provides a numerically stable and physically consistent description of the optical conductivity spectra while enabling direct comparison of spectral evolution across the full doping series.

The measured spectra, individual oscillator contributions, total fitted curves, and residuals are shown in Figure 3(d) and Supplementary Figure 18(b), and the corresponding fitting parameters and R^2 values are summarized in Supplementary Table 3.

(4) The difference between O *K*-edge XAS and optical conductivity results

A point that deserves clarification is that the O *K*-edge XAS and optical conductivity measurements do not probe the same physical quantity, and therefore their spectral weights (intensity) should not be expected to exhibit a strict one-to-one correspondence. O *K*-edge XAS measures transitions from the O 1*s* core level into unoccupied O 2*p*-derived states above the Fermi level and is thus primarily sensitive to the distribution of unoccupied electronic states and their orbital character [Figures. 3(e)-(f)]. By contrast, optical conductivity probes interband excitations between occupied and

unoccupied states and therefore reflects the transition matrix elements and joint evolution of both sides of the electronic structure [Figures. 3(e)-(f)]. Consequently, although the XAS features A and A* and the optical features α and α* originate from the same underlying doping-induced reconstruction of the electronic structure, their absolute intensities and doping dependences are not required to evolve identically. In addition, XAS and optical measurements were carried out under different incident-light angles, which introduce different sensitivities to in-plane and out-of-plane orbital components. Accordingly, the most physically meaningful comparison between the two techniques lies not in an exact correspondence of spectral weight, but rather in their consistent identification of the same doping-driven electronic-structure evolution and the emergence of additional electronic states in the heavily overdoped regime.

Supplementary Note 5. Oxygen vacancies in heavily overdoped LSCO

The temperature-dependent resistivity for various doping levels is shown in Supplementary Figure 19. As the doping level increases up to x = 0.4, the resistivity decreases, which is attributed to an increase in carrier concentration due to hole doping. However, at higher doping levels (x > 0.4), the resistivity in turn increases, consistent with previous reports on LSCO [16, 17]. This upturn in resistivity may be ascribed to the formation of oxygen deficiencies at high doping levels, leading to enhanced carrier scattering and disorder-induced localization effects.

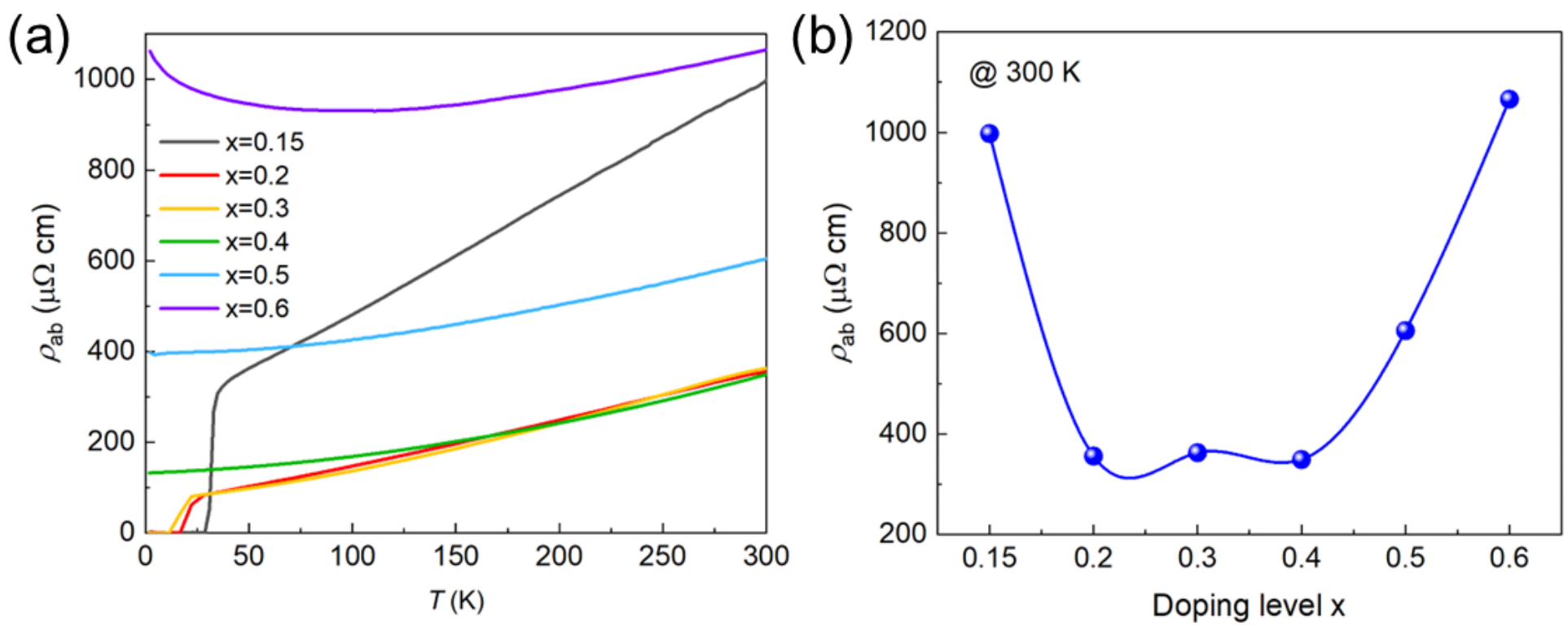

Supplementary Figure 19. The temperature-dependent resistivity for various doping levels.

The presence of oxygen deficiencies at higher overdoping levels is supported by changes in the lattice structure. Supplementary Figure 20(a) displays the XRD patterns around the (006) Bragg peak. While the (006) peak of the LSAO substrate (♦) remains unchanged with doping, the corresponding peak of the LSCO films (♣) shifts to lower angles as x increases up to 0.4, and then shifts back to higher angles for x > 0.4. The out-of-plane lattice parameter c, extracted from the XRD data and plotted in Supplementary Figure 20(b), exhibits a doping-dependent trend that closely mirrors the behavior of the resistivity.

In the doping range $0.15 \leq x \leq 0.4$, the c-axis parameter increases monotonically, which is attributed to interfacial strain effects between the LSCO films and the LSAO substrate. In contrast, for $x \geq 0.4$, a sharp decrease in c is observed, which is consistent with lattice contraction due to oxygen deficiency [17].

Because oxygen nonstoichiometry can influence O K-edge spectral line shapes in complex oxides, the possibility that the newly observed A* and α* features are primarily defect-related was considered. Oxygen-vacancy-related effects may contribute to spectral broadening and background variations, particularly in the most highly doped LSCO films. However, A* in the O K-edge XAS and α* in the optical conductivity emerge already for $x > 0.2$, whereas the transport and lattice signatures most consistent with oxygen deficiency become evident mainly at higher doping levels. This mismatch in onset, together with the concurrent appearance of A* and α* in two independent spectroscopic probes and the vacancy-free DQMC results, indicates that oxygen vacancies are unlikely to be the primary origin of the new spectral features. Accordingly, the A* and α* features are interpreted primarily as manifestations of a doping-induced reconstruction of Zhang–Rice-related electronic states, while oxygen nonstoichiometry may provide a secondary contribution, particularly in the most heavily doped films.

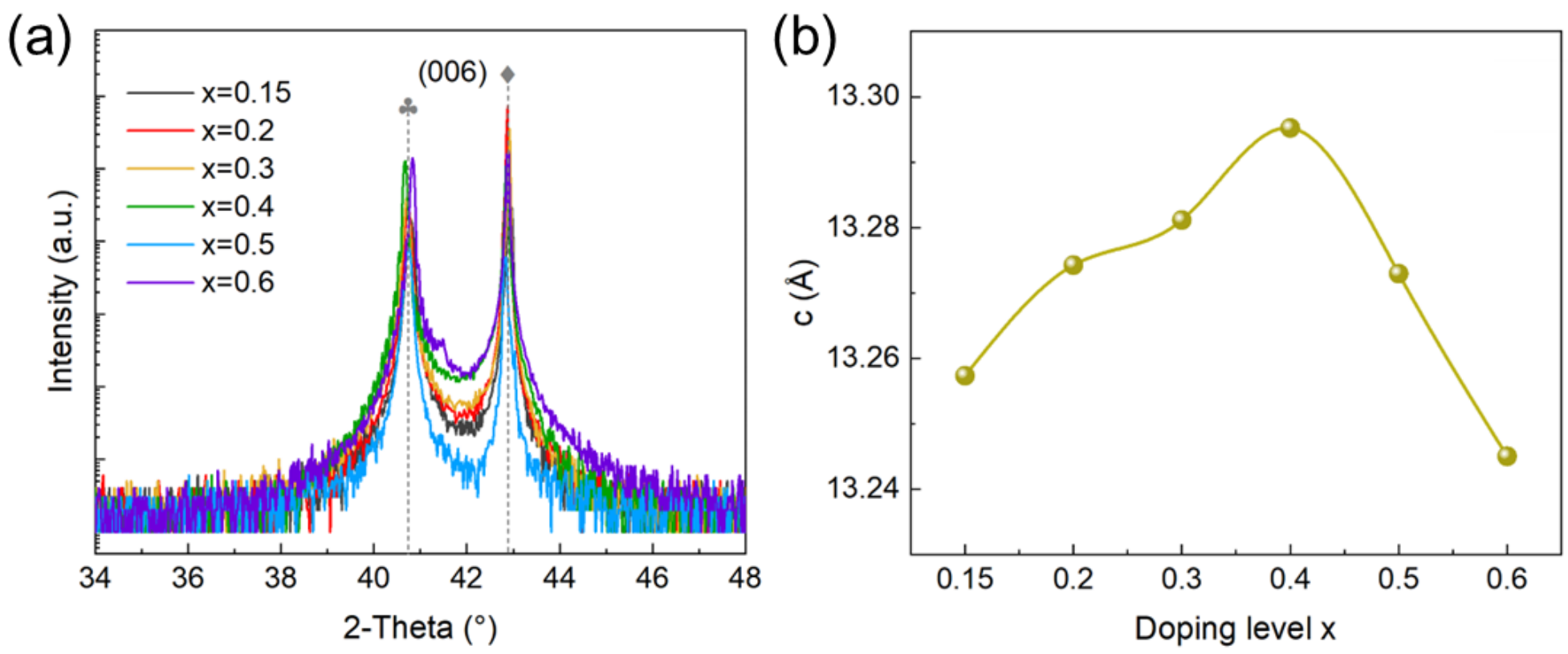

Supplementary Figure 20. (a) The XRD patterns around the (006) Bragg peak with different doping levels. (b) Doping dependence of the out-of-plane lattice parameter c.

Supplementary Note 6. Electrical Resistivity Fitting model of heavily overdoped LSCO

According to the established LSCO phase diagram, the system evolves from exhibiting strange-metal behavior to Fermi-liquid behavior as the doping level increases from x = 0.15 to 0.6 [18]. Therefore, the resistivity properties of LSCO change significantly at different doping levels. When analyzing resistivity data, a widely used unified approach for fitting the in-plane resistivity of overdoped cuprates employs a single theoretical model across the entire temperature range, typically expressed as $\rho(T) = \mathrm{a} + \mathrm{b}T +$

cT^2 [19].

This empirical expression, consisting of both linear and quadratic terms in temperature, describes a smooth crossover from a T^2 dependence at low temperatures to a linear T dependence at higher temperatures. In this model, a represents the residual resistivity, the T^2 term reflects Fermi-liquid-like scattering processes, while the linear T term is associated with additional scattering mechanisms, likely arising from strong electron correlations at elevated temperatures [20]. Empirically, this two-term form fits many overdoped LSCO resistivity curves well across the full measured temperature range. The theoretical basis for simply adding the T and T^2 terms relies on the assumption that two independent scattering mechanisms contribute to the total resistivity, consistent with Matthiessen's rule [21]. In a metallic system, if different scattering processes act in parallel—such as electron-electron scattering and bosonic scattering—their scattering rates add, allowing ρ(T) to be approximated as the sum of these separate contributions.

Supplementary Note 7. Three-orbital Emery theoretic model

7.1. The determinant quantum Monte Carlo (DQMC) and maximum entropy analytic continuation (MaxEnt) method

The determinant quantum Monte Carlo (DQMC) algorithm is a well-established finite-temperature method to simulate manifold types of strongly correlated electron models such as the periodic Anderson model, the single-orbital Hubbard model and the three-orbital Emery model [22-25]. In this algorithm, the expectation values of an observable are computed in the grand canonical ensemble

$$\langle \hat{O} \rangle = \frac{\mathrm{Tr}[\hat{O} e^{-\beta H}]}{\mathrm{Tr}[e^{-\beta H}]} \tag{S12}$$

with the imaginary-time interval $[0, \beta]$ divided into L slices of width $\Delta\tau$. Then the exponential can be decomposed using the Trotter approximation such that

$$e^{-L\Delta\tau H} \approx (e^{-\Delta\tau E_s} e^{-\Delta\tau K} e^{-\Delta\tau U_{dd}} e^{-\Delta\tau U_{pp}})^L.$$

A trace over the quadratic terms can be evaluated directly whereas the interaction terms are quartic in the fermion operators and require more care. One can introduce auxiliary Hubbard-Stratonovich fields to transform them to quadratic form, i.e.

$$e^{-\Delta\tau U_{\alpha\alpha} n_{i,\uparrow}^{\alpha} n_{i,\downarrow}^{\alpha}} = \frac{1}{2} \sum_{s_{i,\alpha,l}} s_{i,\alpha,l} e^{\lambda_\alpha s_{i,\alpha,l}(n_{i\uparrow}^{\alpha} - n_{i\downarrow}^{\alpha}) - \frac{1}{2}\Delta\tau U_{\alpha\alpha}(n_{i\uparrow}^{\alpha} + n_{i\downarrow}^{\alpha})} \tag{S13}$$

at each site m and time slice l where α is the orbital index. Here λ_α is defined by $\tanh^2(\lambda_\alpha/2) = \tanh(\Delta\tau U_{\alpha\alpha}/4)$. Once the interacting terms are rewritten in a quadratic form, we can evaluate the trace over the fermionic degree of freedom to obtain an expression for the partition function in terms of matrix determinants,

$$Z = \sum_{s_{m,\alpha,l}=\pm 1} \det M^{+} \det M^{-} \tag{S14}$$

where $M^{\sigma} = I + \prod_{l=L}^{1} B_l^{\sigma}$ and $B_l^{\pm} = e^{-\Delta\tau K} e^{v_{\pm}^{d}(l)} e^{v_{\pm}^{p}(l)}$. Here I is the identity matrix and the elements of matrices are

$$v_{\pm}^{\alpha}(l)_{mm'} = \delta_{mm'} \left[\pm\lambda_{\alpha} s_{m,\alpha,l} - \Delta\tau \frac{U_{\alpha\alpha}}{2} \right]. \tag{S15}$$

Then by sampling the Hubbard-Stratonovich fields using the Markov chain Monte Carlo method where the system accepts proposed local and global changes using a modified heat bath algorithm, the observable $\langle \hat{O} \rangle$ can therefore be calculated. The weight of a specific Hubbard-Stratonovich field configuration is not always positive definite. This is the specific manifestation of the infamous fermion sign problem in DQMC. Hence the average fermion sign f_{sign} must be included and the expectation values are then reweighted as

$$\langle \hat{O} \rangle = \frac{\sum_{s_{m,\alpha,l}} \hat{O} f_{\text{sign}} P(s)}{\sum_{s_{m,\alpha,l}} f_{\text{sign}} P(s)}, \tag{S16}$$

where $P(s) = |\det M^{+} \det M^{-}/Z|$.

In order to obtain the density of states (DOS) which are defined at real frequencies, the imaginary-time Green's functions can be analytically continued. Specifically, recovering the spectrum from the following equation is a typical *ill-conditioned* problem in which small errors on Green's functions can greatly amplify the ones on the spectrum $A(\omega)$.

$$G(\tau) = \int_{-\infty}^{\infty} \frac{d\omega}{2\pi} \frac{e^{-\omega\tau} A(\omega)}{1 \pm e^{-\beta\omega}} \tag{S17}$$

The Maximum Entropy (MaxEnt) Method can extract the least biased spectral function from all the feasible solutions by minimizing

$$Q = \chi^2 - \alpha S, \tag{S18}$$

where χ^2 is a measure of goodness-of-fit and the differential entropy is defined as

$$S = -\int_{-\infty}^{\infty} \frac{d\omega}{2\pi} A(\omega) \ln \frac{A(\omega)}{D(\omega)}. \tag{S19}$$

where $D(\omega)$ is called the *default model* and α is a parameter to be determined [26, 27].

7.2. Simulating three-orbital Emery model with determinant quantum Monte Carlo and maximum entropy analytic continuation method

In order to further check the origin of the anomalous low energy behavior, we perform numerically unbiased determinant quantum Monte Carlo simulation on a 8×8 square lattice within the framework of three-orbital Emery model [28]. As illustrated in Figure 4(a), the three-orbital model only include $d_{x^2-y^2}$ and $p_{x/y}$ orbitals among which the phase convention of hopping terms in hole language is clearly labeled. Without assuming the existence of ZRS in advance, the three-orbital model represents the minimal model complexity which successfully captures the essential physics [22]. We employ a parameter set close to that of cuprates, with the d-p hopping term taken as the energy unit. The descriptions of parameters and their detailed values are listed in Supplementary Table 4. Here, the parameters of primary interest are U_{pp} and the inverse temperature β. The full Hamiltonian reads as follows.

$$\hat{H} = \hat{E}^s + \hat{K} + \hat{U} \tag{S20}$$

$$\hat{E}^s = (\epsilon_d - \mu)\sum_{i\sigma} \hat{n}_{i\sigma}^d + (\epsilon_p - \mu)\sum_{j\sigma} \hat{n}_{j\sigma}^p \tag{S21}$$

$$\hat{K} = \sum_{\langle ij\rangle\sigma} t_{pd}^{ij}\left(\hat{d}_{i\sigma}^\dagger \hat{p}_{j\sigma} + h.c.\right) + \sum_{\langle jj'\rangle\sigma} t_{pp}^{jj'}\left(\hat{p}_{j\sigma}^\dagger \hat{p}_{j'\sigma} + h.c.\right) \tag{S22}$$

$$\hat{U} = U_{dd}\sum_i \hat{n}_{i\uparrow}\hat{n}_{i\downarrow} + U_{pp}\sum_j \hat{n}_{j\uparrow}\hat{n}_{j\downarrow} \tag{S23}$$

Here i or j is the label of Cu or O orbitals and σ stands for spin. The notation $\langle ij\rangle$ and $\langle jj'\rangle$ indicate that the summation is restricted to nearest-neighbor pairs of lattice sites. The superscripts of t_{pd} and t_{pp} represent the sign of their phase which are already labeled in Figure 4(a). Specifically, the onsite Coulomb repulsion $U_{dd} = 6.0$ and $U_{pp} = 0, 1.0, 2.0$ for d or p orbitals. The onsite energy difference between d and p orbitals is set to $\Delta = \epsilon_p - \epsilon_d = 3.0$. Then the hopping of nearest-neighbor (NN) d-p or p-p bonds are $t_{pd} = 1.0$ or $t_{pp} = 0.4$. In the grand canonical ensemble, the total density of holes can be tuned by the chemical potential μ. Using The DQMC algorithm works on discrete imaginary-time slices, with an imaginary-time step of $\Delta\tau = 0.1$ for inverse temperature βt_{pd} = 8.0 and 10.0. where the inverse temperature $\beta t_{pd} = 10$ is divided into 160 steps. Hence in order to deal with the *ill-posed* problem caused by the inversion of the fermionic imaginary-time Green's function to obtain the spectral function via

$$G(\boldsymbol{k},\tau) = \int_{-\infty}^{+\infty} \frac{d\omega}{2\pi} \frac{e^{-\omega\tau} A(\boldsymbol{k},\omega)}{1+e^{-\beta\omega}}, (2) \tag{S24}$$

we adopt the maximum entropy analytic continuation method (MaxEnt) to extract the

least biased spectral function from all feasible solutions [26, 27]. Then integrating the spectral function in all $\boldsymbol{k}$ point yields the local density of states (DOS), which is the focus of the following subsection.

Supplementary Table 4. Parameters used in the DQMC simulation.

Parameter	Value	Description
U_{dd}	6.0	Interaction on $d_{x^2-y^2}$
U_{pp}	0, 1.0 and 2.0	Interaction on $p_{x/y}$
$\Delta = \epsilon_p - \epsilon_d$	3.0	Charge transfer energy
t_{pd}	1.0 as unit	Hopping between $d_{x^2-y^2}$ and $p_{x/y}$
t_{pp}	0.4	Hopping between p_x and p_y
β	8.0 and 10.0	Inverse temperature
N_c	8×8	Number of unit cells

The superscripts associated with t_{pd} and t_{pp} denote the phase signs of the corresponding hopping integrals, as illustrated in Figure 4a. The calculations are based on the three-orbital Emery model with an on-site Coulomb repulsion of U_{dd}=6.0 on the Cu *d*-orbitals and U_{pp}=0, 1.0, or 2.0 on the O *p*-orbitals. The charge-transfer energy is fixed at $\Delta=\varepsilon_p-\varepsilon_d=3.0$, while the nearest-neighbour hopping amplitudes are t_{pd}=1.0 and t_{pp}=0.4. Within the grand-canonical ensemble, the hole concentration is controlled through the chemical potential μ. The DQMC simulations are performed using a discrete imaginary-time formulation with a time step of $\Delta\tau$=0.1 for inverse temperatures βt_{pd}=8.0 and 10.0. For βt_{pd}=10, the imaginary-time interval is discretized into 160 time slices.

7.3. Additional O feature peak above the Fermi level

As the hole density labeled on the right, Figure 4(b) shows the local DOS in a considerable hole density range from 1.0, i.e. half-filling in the hole language, to 1.6 for the purpose of examining the experimental results. When the doping level increases, the red Cu-feature peak upon the Fermi level, which is commonly identified as the UHB [29], moves toward higher energy with the spectral weight significantly reduced. The broad blue band is the charge transfer band which locates around -3 eV beneath the Fermi level. Due to a relatively high simulating temperature, the lower Hubbard band is emerged in the background. Particularly noteworthy are the characteristics of the low-

energy spectra. In a doping range of n=1.1~1.4, the so-called ZRS remains intact and shows strong d-p coupling feature which is understandable. However, when the doping level keeps increasing, an O-feature peak naturally emerges at approximately 0.5 eV above the Fermi level. This peak continues to move to higher energy until the doping level reaches 1.6 along with a rising weight. Besides, the Cu spectra near the Fermi level are more robust against doping. When doped to n~1.5, the spectra begin to be less stable and show a signature of splitting. On the contrary, the spectra of O on the Fermi level maintain complete until n=1.6 doping.

Supplementary Figure 21 corresponds to different values of $U_{\rm pp}$ ranging from 0 to 2, or inverse temperatures β equals to 8 or 10. Supplementary Figure 21(a, d) present the results obtained with an increased oxygen on-site Coulomb interaction, $U_{\rm pp}$=2, While Supplementary Figure 21(b,c) correspond to reduced values of $U_{\rm pp}$=1.0 and 0. A systematic decrease in $U_{\rm pp}$ leads to a noticeable reduction in the critical hole concentration at which the low-energy spectral peak begins to split, indicating that $U_{\rm pp}$ quantitatively influences the onset of the spectral reconstruction. Supplementary Figure 21(d) further shows results obtained at a higher temperature (β = 8). The overall spectral evolution remains qualitatively similar to that observed for (β= 10), with the splitting of the low-energy spectral peak emerging only for hole doping levels $p_{\rm h}$ exceeding ~0.4. This observation suggests that the spectral splitting is governed predominantly by hole doping, while exhibiting only a weak dependence on the simulation temperature within the parameter range considered.

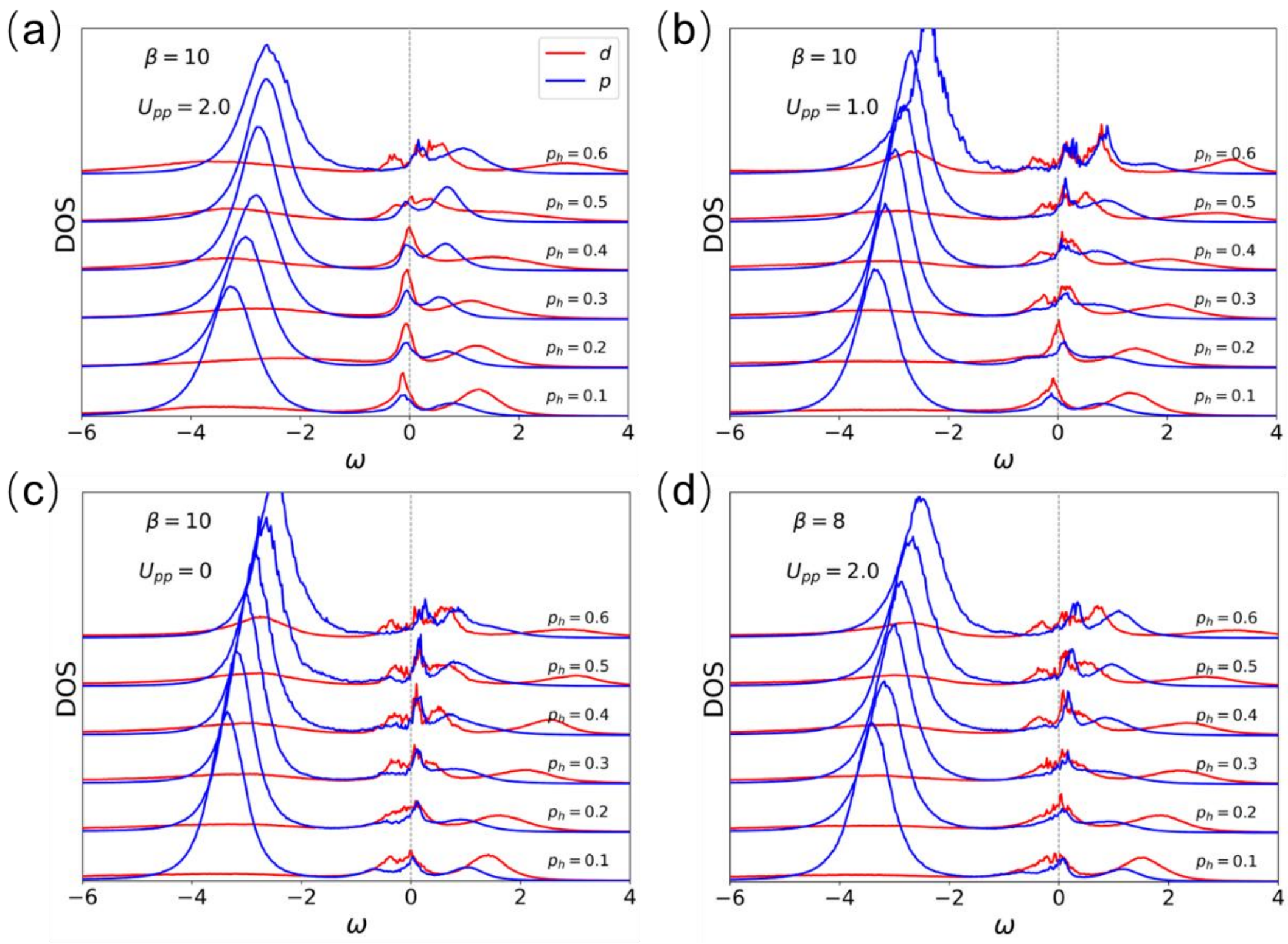

Supplementary Figure 21. DQMC-calculated density of states (DOS) of LSCO for different interaction parameters and temperatures. (a) with β = 10 and $U_{\rm pp}$ = 2.0, (b)

with β = 10 and U_{pp} = 1.0, (c) with β = 10 and U_{pp} = 0, (d) with β = 8 and U_{pp} = 2.0 for varying doping levels p_h. The red and blue curves correspond to the projected DOS of the Cu and O orbitals, respectively.

The main spectroscopic comparison is based on the 300 K XAS and OSE data, which show consistent doping-dependent evolution. The weak temperature dependence of the OSE spectra, together with additional temperature-dependent DQMC results, indicates that the key 1–2 eV spectral features are governed primarily by doping rather than thermal fluctuations. This supports the interpretation that the observed electronic-structure reconstruction is an intrinsic property of the normal state.

7.4. Relationship with previous works and experimental results

Although $\beta = 10$ is still high when compared with the experiment, there are opinions that DQMC can reproduce essential phenomena without really reaching the necessary temperature [22]. We are not the first group to theoretically identify indications of ZRS splitting within model calculations; earlier studies have already found new quasi-peaks near the Fermi level [30, 31]. However, no signature of unstable ZRS is observed in our simulation when the doping level n is less than 1.4. Here the evolution of the spectra is akin to the dynamical spectral weight transfer scenario proposed by George et al [29, 32]. On the contrary, the high Cu–O coupling feature, which is commonly believed to be associated with the formation of ZRS, is somewhat reduced in the extremely high doped regime. The additional O-feature peak seems originate from the transferred spectral weight of UHB and reside upon the ZRS band. Hence it is detected by the O-K edge XAS and the optical conductivity. Since the ZRS is formed by the linear combination of one hole on Cu-$3d$ orbital and the other on the four ligand O-$2p$ orbitals, it should be sensitive to the hole densities on Cu and O [33]. In the charge transfer regime, the doped holes mainly reside on O orbitals. This may lead to a gradual deviation of the Cu–O hole concentration ratio from the stability condition of the ZRS. Obviously, further investigation of the $\boldsymbol{k}$-resolved spectral dispersion will be essential in future studies.

Supplementary Reference

1. Dong Y*, et al.* Interfacial octahedral manipulation imparts hysteresis-free metal to insulator transition in ultrathin nickelate heterostructure. *Advanced Materials Interfaces* **6**, 1900644 (2019).

2. Ketenoglu D. A general overview and comparative interpretation on element-specific X-ray spectroscopy techniques: XPS, XAS, and XRS. *X-Ray Spectrometry* **51**, 422-443 (2022).

3. CONCHA C, GARCIA J, BLASCO J, K. B. GARG RKS, D. CHATURVEDI. Polarised temperature dependent XANES study of superconducting $La_{2-x}Sr_xCuO_{4-y}$ under- and over-doped single crystals. *International Journal of Modern Physics B* **16**, 1327-1339 (2002).

4. Chen CT*, et al.* Electronic states in $La_{2-x}Sr_xCuO_{4+\delta}$ delta probed by soft-x-ray absorption. *Physical Review Letters* **66**, 104-107 (1991).

5. Taguchi M*, et al.* Evidence for suppressed screening on the surface of high temperature $La_{2-x}Sr_xCuO_4$ and $Nd_{2-x}Ce_xCuO_4$ superconductors. *Physical Review Letters* **95**, 177002 (2005).

6. Chen CT*, et al.* Out-of-plane orbital characters of intrinsic and doped holes in $La_{2-x}Sr_xCuO_4$. *Physical Review Letters* **68**, 2543-2546 (1992).

7. Zhang Z*, et al.* Nematicity and Charge Order in Superoxygenated $La_{2-x}Sr_xCuO_{4+y}$. *Physical Review Letters* **121**, 067602 (2018).

8. Li WM*, et al.* Superconductivity in a unique type of copper oxide. *Proceedings of the National Academy of Sciences of the United States of America* **116**, 12156-12160 (2019).

9. Cancellieri C, Strocov VN. *Spectroscopy of complex oxide interfaces*. Springer International Publishing (2018).

10. Bianconi A, Budnick J, Flank AM, A. Fontaine PL, A. Marcelli, H. Tolentino, B. Chamberland, C. Michel, B. Raveau, G. Demazeau. Evidence of $3d^9$-ligand hole states in the superconductor $La_{1.85}Sr_{0.15}CuO_4$ from L_3 X-ray absorption spectroscopy. In: *Physics Letters A*) (1988).

11. Yin X*, et al.* Unraveling how electronic and spin structures control macroscopic properties of manganite ultra-thin films. *NPG Asia Materials* **7**, e196-e196 (2015).

12. Wong FJ, Hong N, Ramanathan S. Orbital splitting and optical conductivity of the insulating state of NbO2. *Physical Review B* **90**, 115135 (2014).

13. Liu X*, et al.* Small polarons mediated near-room-temperature metal–insulator transition in vanadium dioxide and their hopping dynamics. *Applied Physics Reviews* **12**, 011403 (2025).

14. Lee-Hone NR, Mishra V, Broun DM, Hirschfeld PJ. Optical conductivity of overdoped cuprate superconductors: application to $La_{2-x}Sr_xCuO_4$. *Physical Review B* **98**, 054506 (2018).

15. Vial A, Grimault A-S, Macías D, Barchiesi D, de la Chapelle ML. Improved analytical fit of gold dispersion: application to the modeling of extinction spectra with a finite-difference time-domain method. *Physical Review B* **71**, 085416 (2005).

16. Takagi H, Ido T, Ishibashi S, Uota M, Uchida S, Tokura Y. Superconductor-to-nonsuperconductor transition in $(La_{1-x}Sr_x)_2CuO_4$ as investigated by transport and magnetic measurements. *Physical Review B* **40**, 2254-2261 (1989).

17. Sato H, Tsukada A, Naito M, Matsuda A. $La_{2-x}Sr_xCuO_y$ epitaxial thin films (x = 0 to 2): structure, strain, and superconductivity. *Physical Review B* **61**, 12447 (2000).

18. Takagi H*, et al.* Systematic evolution of temperature-dependent resistivity in $La_{2-x}Sr_xCuO_4$. *Physical Review Letters* **69**, 2975-2978 (1992).

19. Cooper RA*, et al.* Anomalous criticality in the electrical resistivity of $La_{2-x}Sr_xCuO_4$. *Science* **323**, 603-607 (2009).

20. Legros A*, et al.* Universal T-linear resistivity and Planckian dissipation in overdoped cuprates. *Nature Physics* **15**, 142-147 (2018).

21. Feng T, Qiu B, Ruan X. Coupling between phonon-phonon and phonon-impurity scattering: a critical revisit of the spectral Matthiessen's rule. *Physical Review B* **92**, 235206 (2015).

22. Kung YF*, et al.* Characterizing the three-orbital Hubbard model with determinant quantum Monte Carlo. *Physical Review B* **93**, 155166 (2016).

23. Wang M, Hu D, Yang Y-f. Insulator-to-insulator transition and sign problem in the periodic Anderson model with a staggered potential. *Physical Review B* **110**, 195122 (2024).

24. Moritz B*, et al.* Effect of strong correlations on the high energy anomaly in hole- and electron-doped high-Tcsuperconductors. *New Journal of Physics* **11**, 093020 (2009).

25. Mai P, Balduzzi G, Johnston S, Maier TA. Pairing correlations in the cuprates: a numerical study of the three-band Hubbard model. *Physical Review B* **103**, 144514 (2021).

26. Bergeron D, Tremblay AMS. Algorithms for optimized maximum entropy and diagnostic tools for analytic continuation. *Physical Review E* **94**, 023303 (2016).

27. Mark Jarrell JEG. Bayesian inference and the analytic continuation of imaginary-time quantum Monte Carlo data. *Physics Reports* **269**, 133-195 (1996).

28. Blankenbecler R, Scalapino DJ, Sugar RL. Monte Carlo calculations of coupled boson-fermion systems. I. *Physical Review D* **24**, 2278-2286 (1981).

29. Eskes H, Meinders MBJ, Sawatzky GA. Anomalous transfer of spectral weight in doped strongly correlated systems. *Physical Review Letters* **67**, 1035-1038 (1991).

30. Chen CC*, et al.* Doping evolution of the oxygen K-edge x-ray absorption spectra of cuprate superconductors using a three-orbital Hubbard model. *Physical Review B* **87**, 165144 (2013).

31. Weber C, Haule K, Kotliar G. Apical oxygens and correlation strength in electron- and hole-doped copper oxides. *Physical Review B* **82**, 125107 (2010).

32. Meinders MB, Eskes H, Sawatzky GA. Spectral-weight transfer: Breakdown of low-energy-scale sum rules in correlated systems. *Phys Rev B Condens Matter* **48**, 3916-3926 (1993).

33. Zhang FC, Rice TM. Effective Hamiltonian for the superconducting Cu oxides. *Physical Review B* **37**, 3759-3761 (1988).
