# Supplementary material for: Checkerboard-type Zhang-Rice states in overdoped cuprate superconductors
Source: Nat Commun. 2026 Sep 19;17:10975. doi: 10.1038/s41467-026-77985-0 (PMC13683379; doi:10.1038/s41467-026-77985-0)
Supplement: Supplementary file 2 [file 41467_2026_77985_MOESM2_ESM.pdf]

nature portfolio

Peer Review File

Checkerboard-type Zhang-Rice States in Overdoped Cuprate Superconductors

Corresponding Author: Professor Xinmao Yin

This file contains all reviewer reports in order by version, followed by all author rebuttals in order by version.

Version 0:

Reviewer comments:

Reviewer #1

(Remarks to the Author)
This manuscript presents a systematic spectroscopic study of LSCO thin films across a wide doping range (x=0.15-0.60) using high-resolution X-ray absorption spectroscopy and optical spectroscopic ellipsometry. Combined with determinant quantum Monte Carlo (DQMC) simulations of the three-orbital Emery model, it reports the emergence of new spectral features (A* and α*) in the heavily overdoped region (x>0.2). These findings provide important experimental evidence and theoretical insights into the evolution of the electronic structure in overdoped cuprates, the breakdown of the conventional Zhang-Rice singlet picture in this regime, and the possible electronic state reconstruction (e.g., checkerboard-type configuration). The experimental work is solid and the main conclusions are scientifically valuable.

1. Section 4 presents the doping evolution of the density of states from DQMC simulations, noting a split in spectral features at ph=0.5, which is stated to be "consistent" with the experimental observation of ZRS breakdown.

Could more directly comparable quantities be extracted from the DQMC calculations? For instance, could the simulated spectral functon(not just local DOS) show weight transfer or emergence of new peaks in the relevant energy range (corresponding to the experimental 1.1 eV and 1.5 eV features)? Could the calculated optical conductivity (even at finite temperature/with approximations) reproduce, in trend, the experimental emergence of the α* peak and evolution of the α peak? Such direct spectral comparisons would significantly strengthen the theory-experiment link.

2. The specific numerical values (or ranges) of parameters used in the DQMC simulations should be clearly listed in the main text or supplementary materials, with a brief discussion on their reasonableness compared to estimated physical parameters for LSCO. The role of Upp is emphasized in the introduction and discussion; what value was used for Upp in the simulations? Could varying Upp more directly drive the simulated electronic structure reconstruction beyond a specific doping threshold, similar to experiments?

3. Could analysis of local density of states distribution, hole density distribution, or spin-spin correlation functions from the DQMC simulations probe whether there is a tendency towards real-space inhomogeneity or some type of charge/orbital order in the heavily overdoped regime? Even if a perfect "checkerboard" is not reproduced, providing simulation hints that electronic states may tend towards a non-uniform distribution would make the combination of theory and experimental conjecture more compelling.

In summary, the authors need to substantially strengthen the connection between Section 4 (DQMC simulations) and Section 3 (experimental results) by providing more direct comparisons of spectroscopic quantities, clearer parameter correspondence, and exploring the implications of simulation results for spatial configurations,or clearly stating their limitations.

Upon completing these revisions, the manuscript may meet the publication standards.

Reviewer #2

(Remarks to the Author)

This study presents a comprehensive investigation into the electronic structure evolution of LSCO from optimal doping (x=0.15) to heavy over-doping (x=0.60). Combining high-resolution X-ray absorption spectroscopy (XAS), optical spectroscopic ellipsometry (OSE), and determinant quantum Monte Carlo (DQMC) simulations, the authors proposed a doping-induced checkerboard-type Zhang–Rice electronic configuration of over-doped LSCO. Although the authors have proposed a new and seemingly impressive model to explain the electronic structure in heavily doped materials, the experimental evidence is overly limited and cannot rule out issues such as oxygen vacancies introduced during sample preparation. This oversimplified and idealized model lacks rigorous experimental support and therefore fails to meet the journal's publication criteria. I do not recommend its publication in Nature Communication. Several comments are listed below.

Comment 1: XAS results and interpretation

The authors attempt to demonstrate electronic structure changes through the evolution of the O K-edge absorption spectra with doping concentration and the emergence of the new A* peak, utilizing peak fitting analysis. However, I have identified significant discrepancies in the peak positions (e.g., features A and A*) between Figure 2 in the main text and Supplementary Figure S7. The peak fitting procedure in Figure 2(e) appears overly arbitrary, with critical details inadequately described—such as the criteria for baseline selection, the determination of the A* peak position, and the full width at half maximum (FWHM) constraints. Notably, the A* peak position appears to shift across the doping range x=0.3–0.6. This raises a fundamental question: does this shift arise from fitting artifacts, or does it reflect a genuine evolution of the electronic structure?

Furthermore, the A* contribution is more pronounced in the 25° incidence geometry. What is the underlying physical mechanism for this angular dependence? A more rigorous analysis of the A* feature under different polarization conditions—particularly at 25°—could provide stronger evidence for electronic structure modification, yet the authors have not pursued this line of investigation.

The authors claimed: A pronounced drop in spectral intensity is observed at x = 0.6, which may be attributed to a transfer of spectral weight to higher absorption energies" in the main text. However, this is not true, since the transfer of spectral weight is also shown for x=0.5 without the drop in spectral intensity.

I wonder why the Cu L-edge XAS are not involved in the investigation, which certainly helps to understand the electronic structure of LSCO.

Comment 2: Optical spectroscopic ellipsometry results

The data analysis in Figure 3(d) exhibits similar problems to the XAS fitting procedure. Key parameters including precise peak positions, full width at half maximum (FWHM) values, and fitting constraints remain unspecified. The absence of clear criteria for the Drude-Lorentz fitting methodology undermines the reliability of the extracted spectral features.

A significant discrepancy emerges when comparing the doping-dependent intensity variations between optical and XAS measurements: In the optical conductivity spectra, the α* feature surpasses the α feature in intensity with increasing doping (x). Conversely, in the XAS spectra, the A* feature shows relatively weaker enhancement compared to feature A. This contradictory behavior raises critical questions about the interpretation: Does this inconsistency reflect fundamentally different physical origins of the α* and A* features? Could methodological artifacts in the fitting procedures account for the divergent trends? What is the theoretical basis for expecting parallel evolution between these spectroscopically distinct features? Besides, is there any relationship between the energy difference between that of A and A*, and α* and α?

The authors must address whether this discrepancy indicates limitations in the proposed checkerboard-type ZR configuration model or reveals more complex electronic reconstruction mechanisms.

Comment 3: Critical issue regarding oxygen defects in samples

The authors have insufficiently addressed the issue of oxygen defects in the main text, relegating crucial descriptions to the supplementary materials. This is particularly problematic for O K-edge studies, where oxygen vacancies exert significant and unavoidable influences on spectral line shapes. While the supplementary materials mention oxygen defect formation for x > 0.4, the authors provide no experimental evidence to conclusively demonstrate the absence of such defects at lower doping levels (x < 0.4). The authors' categorical claim that the 526-530 eV absorption region remains unaffected by oxygen vacancies is scientifically untenable. Existing literature consistently demonstrates that oxygen defects can substantially modify the relative peak intensities between spectral features, energy positions of characteristic absorption edges and the overall spectral line shape and background contribution.

Without rigorous exclusion of oxygen vacancy effects, the proposed electronic structure reorganization and checkerboard-type ZR configuration hypothesis remains speculative. The attribution of spectral changes solely to doping-induced electronic reconstruction requires substantially stronger experimental validation. This fundamental methodological issue must be conclusively addressed to establish the scientific robustness of the proposed model.

Reviewer #3

(Remarks to the Author)

In this manuscript, the authors systematically investigate the electronic structure evolution of heavily overdoped LSCO thin films in the regime of x = 0.15–0.60. This is achieved through a combination of high-resolution X-ray absorption spectroscopy (XAS) , optical spectroscopic ellipsometry (OSE) , and determinant quantum Monte Carlo (DQMC) simulations based on the three-orbital Emery model. The authors successfully observe spectroscopic evidence of Zhang-Rice singlet (ZRS) breakdown, characterized by the emergence of A* and α* peaks in the overdoped region. Furthermore, they creatively propose a "checkerboard-type" physical picture for the electronic spatial configuration. The experimental data is robust, and the theoretical physical picture is clear. This work is of significant importance for advancing our understanding of electron correlation effects and the superconducting mechanisms in heavily overdoped cuprates. Therefore, I recommend the manuscript for acceptance after minor revisions to address the following specific points.

1.Many studies indicate a close correlation between the physical properties of the LSCO system and film thickness. In Section 2.1, the authors state that the film thickness used in this work is 90 nm, but they do not explain the rationale behind choosing 90 nm instead of 80 nm, 100 nm, or other thicknesses. Furthermore, when fitting the optical properties of LSCO using spectroscopic ellipsometry, the film thickness is inevitably required as an initial parameter. Therefore, it is strongly recommended that the authors appropriately zoom out the scale of the STEM image in Figure 1(b) to explicitly demonstrate that the actual film thickness is indeed 90 nm. This direct visual evidence would be much more convincing than merely estimating the thickness based on the number of PLD deposition pulses.
2.The authors present XAS data collected at 300 K, while OSE data spans 5 K to 300 K, and DQMC simulations are performed at an effectively much higher temperature (β=10). The manuscript currently lacks a clear justification for this temperature mismatch. While it is physically reasonable that the high-energy scale electronic structure (eV range, such as ZRS breakdown) is dominated by doping rather than thermal fluctuations (meV range)—a point supported by the temperature-insensitivity of the OSE spectra —the authors must explicitly state this rationale in the main text. I recommend adding a brief discussion explaining why the 300 K XAS results and high-temperature DQMC simulations represent the intrinsic, normal-state electronic structure that directly correlates with the low-temperature OSE and transport findings.
3.In the fifth line of the abstract, the subscripts in the chemical formula "$La_{2-}xSr_xCuO_4$" are not formatted according to standard conventions. Such typographical errors should not appear in a formal manuscript. The authors should conduct a thorough and careful check throughout the entire text to correct this and any similar formatting issues.

Version 1:

Reviewer comments:

Reviewer #1

(Remarks to the Author)
The authors have revised their manuscript based on three referees' comments. The limit of numerical simulations and its weak point that connected with the experimental data has been discussed.
I think the rebuttal is reasonable. The proposed checkerboard picture offers a fresh microscopic viewpoint on the long-standing puzzle of the overdoped cuprate regime. After this major revision, incorporating the substantial new supplementary data and refined discussions, the manuscript may meet the standards for publication in Nature Communications.

Reviewer #2

(Remarks to the Author)
I thank the authors for their detailed responses to my previous round of comments, however, a critical unresolved issue remains concerning the spectral fitting procedures that form the foundation of the manuscript's core conclusions, which I outline below.
The current presentation of fitting results for Figures 2e and 3d does not permit robust assessment of their reliability. Specifically, the figures did not display the total fitted curve overlaid on experimental data, with only individual component peaks, no quantification of agreement between the total fit and raw spectra, and no inclusion of residual plots to evaluate goodness-of-fit. This lack of transparency makes it impossible to verify whether decomposed spectral features (e.g., peaks Z, A*, A) are physically meaningful or artifacts of an inappropriate fitting routine.
My specific concerns about fitting quality are as follows:
For x = 0.15 spectrum (Figure 2e): The raw spectral line shape does not conform to the symmetric profile of a standard Gaussian-Lorentzian (Voigt) function.
For x = 0.5 and x = 0.6 spectra (Figure 2e): The sum spectra (which is missing) of fitted components, obviously deviates from experimental data.
Figure 3d: Similar issues apply. There is no presentation of individual fit components, residuals, or quantitative agreement metrics, making it impossible to verify the fitting results that underpin the discussion of spectral evolution with doping.
This is not a minor technical point: the entire manuscript's narrative regarding electronic structure reconstruction in the heavily over-doped regime, the origin of the A* (and corresponding α* optical) feature, and the comparison to DQMC simulations rests entirely on the reliability of fitting results presented in Figures 2e and 3d. Without transparent documentation of the fitting workflow and convincing evidence that decomposed features match experimental data, these core conclusions lack a solid empirical foundation.
To address these concerns, I request the following revisions:
• Supplement Figures 2e and 3d with panels showing: (i) individual fitted component peaks, (ii) a direct point-by-point overlay of the total fitted curve and raw experimental data, and (iii) a residual spectrum (raw data minus total fit) to visualize systematic deviations.

• Provide a complete table of all fitting parameters in the Supplementary Materials, including for each decomposed peak: center energy, full width at half maximum (FWHM), integrated intensity, Gaussian-Lorentzian mixing ratio, and goodness-of-fit metrics (e.g., R^2, reduced χ^2).
• Include a brief methodological justification for the choice of Gaussian-Lorentzian line shapes, with a specific discussion of the x = 0.15 spectrum's asymmetry and tests of alternative line shape models to confirm robustness of peak assignments.
• Quantify the deviation between the total fitted curve and raw data for the x = 0.15, 0.5, and 0.6 spectra (e.g., root-mean-square error across the 526–529 eV window) to demonstrate that fit quality is acceptable for the claims being made.
Once these revisions are completed and the reliability of fitting results is clearly demonstrated, I will be able to reassess the manuscript's suitability for publication. I appreciate the authors' efforts to address prior feedback, and look forward to evaluating the updated version.

Reviewer #3

(Remarks to the Author)
After being revised, the author of the paper has corrected the relevant errors and answered my questions. At the same time, I also carefully read the author's responses to the questions raised by other reviewers. I believe that this paper has strong theoretical and innovative qualities, and its current overall level has basically met the publication requirements of the journal.

Version 2:

Reviewer comments:

Reviewer #2

(Remarks to the Author)
Given the authors' thorough revision of the spectral fitting protocols,including the transparent presentation of total fits, residuals, and quantitative metrics such as RMSE,I am satisfied that the reliability of the core conclusions is now firmly established, and I recommend the manuscript for publication.

Open Access This Peer Review File is licensed under a Creative Commons Attribution 4.0 International License, which permits use, sharing, adaptation, distribution and reproduction in any medium or format, as long as you give appropriate credit to the original author(s) and the source, provide a link to the Creative Commons license, and indicate if changes were made.
In cases where reviewers are anonymous, credit should be given to 'Anonymous Referee' and the source.
The images or other third party material in this Peer Review File are included in the article's Creative Commons license, unless indicated otherwise in a credit line to the material. If material is not included in the article's Creative Commons license and your intended use is not permitted by statutory regulation or exceeds the permitted use, you will need to obtain permission directly from the copyright holder.
To view a copy of this license, visit https://creativecommons.org/licenses/by/4.0/

Responses to the Reviewers

We are very grateful for the feedback and suggestions that Reviewers #1, #2 and #3 have provided. They are essential to make improvements to our manuscript. In the paragraphs below, we provide our responses to the respective remarks.

Note：All Reviewers' comments/questions are shown in black, while our corresponding responses are annotated in blue and the changes in red.

Reviewer #1 (Remarks to the Author):

This manuscript presents a systematic spectroscopic study of LSCO thin films across a wide doping range (x=0.15-0.60) using high-resolution X-ray absorption spectroscopy and optical spectroscopic ellipsometry. Combined with determinant quantum Monte Carlo (DQMC) simulations of the three-orbital Emery model, it reports the emergence of new spectral features (A* and α*) in the heavily overdoped region (x>0.2). These findings provide important experimental evidence and theoretical insights into the evolution of the electronic structure in overdoped cuprates, the breakdown of the conventional Zhang-Rice singlet picture in this regime, and the possible electronic state reconstruction (e.g., checkerboard-type configuration). The experimental work is solid and the main conclusions are scientifically valuable.

We thank Reviewer #1 for the positive evaluation of our work and for recognizing the importation of the subject and the results presented. In response to the Reviewer's valuable suggestions, we have carefully revised the manuscript to improve clarity of calculated insights and enhance connection between experiment and theory. Additionally, we have included supplementary details to further support our arguments.

1. Section 4 presents the doping evolution of the density of states from DQMC simulations, noting a split in spectral features at ph=0.5, which is stated to be "consistent" with the experimental observation of ZRS breakdown. Could more directly comparable quantities be extracted from the DQMC calculations? For instance, could the simulated spectral function(not just local DOS) show weight transfer or emergence of new peaks in the relevant energy range (corresponding to the experimental 1.1 eV and 1.5 eV features)? Could the calculated optical conductivity (even at finite temperature/with approximations) reproduce, in trend, the experimental emergence of the α* peak and evolution of the α peak? Such direct spectral comparisons would significantly strengthen the theory-experiment link.

We appreciate the Reviewer's suggestion. Indeed, this is a very reasonable point, although it is also technically highly demanding for numerical simulations. In principle, the optical conductivity can be evaluated within DQMC in imaginary time. However, obtaining the real-frequency $\sigma_1(\omega)$ requires analytic continuation from imaginary time to real frequency, which is a well-known ill-posed numerical problem. Even in the existing literature, most conductivity results—including the dc limit—have typically relied on approximations at elevated temperatures and therefore remain somewhat ambiguous, not to mention the much more challenging case of frequency-dependent optical conductivity.

In the present work, this difficulty is further compounded by several factors: (i) optical conductivity is a two-particle correlation function and is therefore substantially noisier than single-particle quantities such as the Green's function, from which the local density of states can be extracted; (ii) the three-orbital nature of the Emery model considerably increases the complexity of the current-current correlations; and (iii) the combination of relatively large system size and low temperature gives rise to a severe fermion sign problem, which further amplifies the statistical uncertainty.

Nevertheless, we did attempt such calculations for several representative cases. However, the resulting spectra were too noisy to be considered reliable, and we therefore felt that it would not be appropriate to present them in a publishable form. Regarding the spectral properties, our recent separate theoretical work [PRB 112, 235147 (2025)] provides more detailed information, including the local density of states and momentum-dependent spectral functions. These results do show spectral-weight transfer, but we do not expect quantitative agreement with the experimental peak positions at 1.1 eV and 1.5 eV.

For these reasons, a reliable reconstruction of $\sigma_1(\omega)$ with sufficient resolution to resolve features on the energy scale of 1–2 eV remains beyond the capability of the present DQMC simulations.

Importantly, this limitation does not affect the main conclusion of the present work, as the observed electronic reconstruction is independently supported by both the experimental measurements and the DQMC-derived evolution of the local density of states.

Changes made to the Manuscript:

In the main text：

Although direct calculation of real-frequency spectral quantities within DQMC remains challenging, the local density of states obtained from our simulations reproduces the essential electronic-structure evolution observed experimentally, thereby supporting the conclusion of electronic reconstruction.

2. The specific numerical values (or ranges) of parameters used in the DQMC simulations should be clearly listed in the main text or supplementary materials, with a brief discussion on their reasonableness compared to estimated physical parameters for LSCO. The role of Upp is emphasized in the introduction and discussion; what value was used for Upp in the simulations? Could varying Upp more directly drive the simulated electronic structure reconstruction beyond a specific doping threshold, similar to experiments?

We are grateful to the Reviewer for this valuable comment. In response, we have revised the manuscript to provide a more detailed description of the DQMC simulations, including the specific numerical parameter values, the physical role of U_{pp}, and the full set of simulation parameters employed.

Our model employs a standard set of parameters for cuprates, with t_{pd} taken as the energy unit, consistent with those widely adopted in previous theoretical studies of cuprate systems, consistent with values widely adopted in previous theoretical studies of cuprate

systems. We have now explicitly specified these parameter choices in the Supplementary Materials. In addition, we have added a new summary table containing all DQMC simulation parameters and expanded the discussion of the dependence on U_{pp}. To further address the Reviewer's concern, we also present the calculated density of states for several values of U_{pp}, thereby explicitly demonstrating its influence on the electronic structure. All parameters used in the DQMC calculations are now fully documented in the revised manuscript and Supplementary Materials to improve transparency and reproducibility.

Changes made to the Manuscript:

In the main text：

The descriptions of parameters and their detailed values are listed in Table S4. Here, the parameters of primary interest are U_{pp} and the inverse temperature β.

In the Supplementary Materials

Fig. S21 corresponds to different values of U_{pp} ranging from 0 to 2, or inverse temperatures β equals to 8 or 10.

Table S4: Parameters used in the DQMC simulation.

Parameter	Value	Description
U_{dd}	6.0	Interaction on $d_{x^2-y^2}$
U_{pp}	0, 1.0 and 2.0	Interaction on $p_{x/y}$
$\Delta = \epsilon_p - \epsilon_d$	3.0	Charge transfer energy
t_{pd}	1.0 as unit	Hopping between $d_{x^2-y^2}$ and $p_{x/y}$
t_{pp}	0.4	Hopping between p_x and p_y
β	8.0 and 10.0	Inverse temperature
N_c	8×8	Number of unit cells

Figs S21(a, d) present the results obtained with an increased oxygen on-site Coulomb interaction, U_{pp}=2, While Figs S21(b,c) correspond to reduced values of U_{pp}=1.0 and 0. A systematic decrease in U_{pp} leads to a noticeable reduction in the critical hole concentration at which the low-energy spectral peak begins to split, indicating that U_{pp} quantitatively influences the onset of the spectral reconstruction. Fig S21(d) further shows results obtained at a higher temperature (β = 8). The overall spectral evolution remains qualitatively similar to that observed for (β= 10), with the splitting of the low-energy spectral peak emerging only for hole doping levels p_h exceeding ~0.4. This observation suggests that the spectral splitting is governed predominantly by hole doping, while exhibiting only

a weak dependence on the simulation temperature within the parameter range considered.

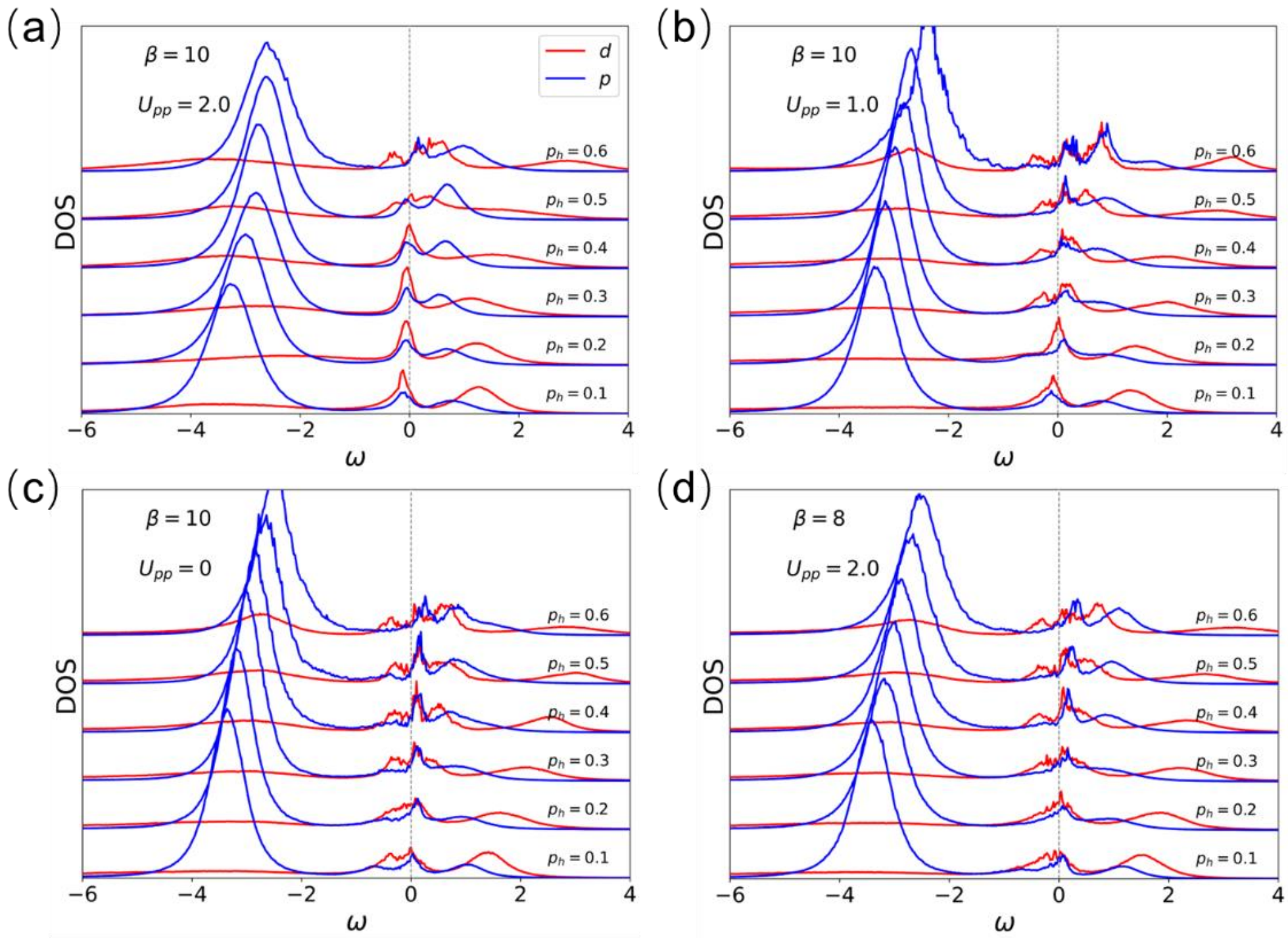

Fig S21. DQMC-calculated density of states (DOS) of LSCO for different interaction parameters and temperatures. (a) with $\beta = 10$ and $U_{pp} = 2.0$, (b) with $\beta = 10$ and $U_{pp} = 1.0$, (c) with $\beta = 10$ and $U_{pp} = 0$, (d) with $\beta = 8$ and $U_{pp} = 2.0$ for varying doping levels p_h. The red and blue curves correspond to the projected DOS of the Cu and O orbitals, respectively.

The superscripts associated with t_{pd} and t_{pp} denote the phase signs of the corresponding hopping integrals, as illustrated in Fig. 4a. The calculations are based on the three-orbital Emery model with an on-site Coulomb repulsion of U_{dd}=6.0 on the Cu *d*-orbitals and U_{pp}=0, 1.0, or 2.0 on the O *p*-orbitals. The charge-transfer energy is fixed at $\Delta=\varepsilon_p-\varepsilon_d=3.0$, while the nearest-neighbour hopping amplitudes are t_{pd}=1.0 and t_{pp}=0.4. Within the grand-canonical ensemble, the hole concentration is controlled through the chemical potential μ. The DQMC simulations are performed using a discrete imaginary-time formulation with a time step of $\Delta\tau$=0.1 for inverse temperatures βt_{pd}=8.0 and 10.0. For βt_{pd}=10, the imaginary-time interval is discretized into 160 time slices.

3. Could analysis of local density of states distribution, hole density distribution, or spin-spin correlation functions from the DQMC simulations probe whether there is a tendency towards real-space inhomogeneity or some type of charge/orbital order in the heavily overdoped regime? Even if a perfect "checkerboard" is not reproduced, providing simulation hints that electronic states may tend towards a non-uniform distribution would make the combination of theory and experimental conjecture more compelling.

We thank the Reviewer for this insightful suggestion. To investigate this possibility, we examined both the local density of states and the charge-correlation functions obtained

from our DQMC simulations in the heavily overdoped regime. Within the range of system sizes and temperatures accessible to the present calculations, we do not observe clear signatures of long-range charge or orbital ordering. Instead, the charge distribution remains largely uniform across the lattice.

These findings are consistent with our recent theoretical study [PRB 112, 235147 (2025)], in which we demonstrated that the spin correlations evolve substantially with doping and exhibit an increasing tendency toward ferromagnetic fluctuations in the heavily overdoped regime, accompanied by a suppression of antiferromagnetic correlations. However, despite these pronounced changes in the spin sector, no corresponding real-space charge inhomogeneity was observed.

Our results are also broadly consistent with previous DQMC studies of purely electronic models, where charge-correlation fluctuations are generally found to remain weak in the absence of additional interactions, such as electron-phonon coupling (e.g., PNAS 121 (50), e2408717121 (2024)).

We note, however, that the accessible DQMC temperature range remains relatively high. In the present simulations, the characteristic temperature scale is on the order of 0.1 eV, corresponding to temperatures well above 10^3 K. Under such conditions, subtle real-space ordering tendencies may be significantly suppressed by thermal fluctuations. Therefore, while our calculations do not provide evidence for charge or orbital ordering, we cannot completely exclude the possibility that such correlations could emerge at lower temperatures. Establishing this conclusively would require substantially lower-temperature simulations and/or alternative many-body approaches, which remain computationally challenging for the three-orbital Emery model.

Changes made to the Manuscript:

In the main text:

We note that the present DQMC simulations do not provide clear evidence for non-uniform real-space charge distribution. This may reflect the limitations imposed by the currently accessible temperature range and system size, under which subtle ordering tendencies can be significantly suppressed.

In summary, the authors need to substantially strengthen the connection between Section 4 (DQMC simulations) and Section 3 (experimental results) by providing more direct comparisons of spectroscopic quantities, clearer parameter correspondence, and exploring the implications of simulation results for spatial configurations, or clearly stating their limitations. Upon completing these revisions, the manuscript may meet the publication standards.

We sincerely thank the Reviewer for raising these important points. In response, we have revised the manuscript to strengthen the connection between Section 4 (DQMC simulations) and Section 3 (experimental results) by clarifying how the simulations support the experimentally observed electronic-structure evolution, while more explicitly discussing the scope and limitations of the present calculations. To improve transparency and

reproducibility, all DQMC simulation parameters are now fully summarized in the newly added Table S4. In addition, we have included the new Fig. S21, which illustrates the effects of U_{pp} and inverse temperature β on the calculated density of states, together with an expanded discussion in the revised manuscript.

Reviewer #2 (Remarks to the Author):

This study presents a comprehensive investigation into the electronic structure evolution of LSCO from optimal doping (x=0.15) to heavy over-doping (x=0.60). Combining high-resolution X-ray absorption spectroscopy (XAS), optical spectroscopic ellipsometry (OSE), and determinant quantum Monte Carlo (DQMC) simulations, the authors proposed a doping-induced checkerboard-type Zhang–Rice electronic configuration of over-doped LSCO. Although the authors have proposed a new and seemingly impressive model to explain the electronic structure in heavily doped materials, the experimental evidence is overly limited and cannot rule out issues such as oxygen vacancies introduced during sample preparation. This oversimplified and idealized model lacks rigorous experimental support and therefore fails to meet the journal's publication criteria. I do not recommend its publication in Nature Communication. Several comments are listed below.

We sincerely thank the Reviewer for the careful reading of our manuscript and for raising these important concerns regarding the experimental support for our conclusions and the possible role of oxygen vacancies.

We would first like to clarify that the primary conclusion of the present work is the observation of substantial electronic-structure evolution in heavily overdoped LSCO, as revealed by XAS, optical spectroscopic ellipsometry (OSE), and transport measurements, and supported by DQMC calculations. The experimental observations include the emergence of new spectral features, the redistribution of spectral weight, and the correlated evolution of transport properties, all of which consistently indicate a reconstruction of the electronic states in the heavily overdoped regime.

The checkerboard-type Zhang-Rice state is proposed as a physically motivated interpretation of these observations rather than as the central experimental claim of the manuscript. In this regard, the checkerboard picture should be viewed as a hypothesis that provides a consistent framework for understanding the observed electronic reconstruction, guided by both the experimental results and the established physics of cuprate systems.

We therefore emphasize that the principal conclusions of this work are based on the experimentally observed electronic-structure evolution, whereas the checkerboard model represents a possible microscopic description of the reconstructed state.

We respectfully address the reviewer's specific concerns below.

Comment 1: XAS results and interpretation

The authors attempt to demonstrate electronic structure changes through the evolution of the O K-edge absorption spectra with doping concentration and the emergence of the new

A* peak, utilizing peak fitting analysis. However, I have identified significant discrepancies in the peak positions (e.g., features A and A*) between Figure 2 in the main text and Supplementary Figure S7. The peak fitting procedure in Figure 2(e) appears overly arbitrary, with critical details inadequately described—such as the criteria for baseline selection, the determination of the A* peak position, and the full width at half maximum (FWHM) constraints. Notably, the A* peak position appears to shift across the doping range x=0.3–0.6. This raises a fundamental question: does this shift arise from fitting artifacts, or does it reflect a genuine evolution of the electronic structure?

1. We thank the Reviewer for the important comments regarding the treatment and peak positions of the O K-edge XAS data. First, concerning the apparent inconsistency in the peak positions of A/A* between Figure 2 in the main text and Supplementary Figure S7, we have carefully re-examined the original data, fitting results, and plotting workflow. Following this review, we identified differences in the plotting convention and energy referencing used in the figures. In the revised manuscript, the energy calibration and plotting conventions have been unified across all XAS spectra to ensure consistency.

Changes made to the Manuscript:

In the main text and supplementary materials:

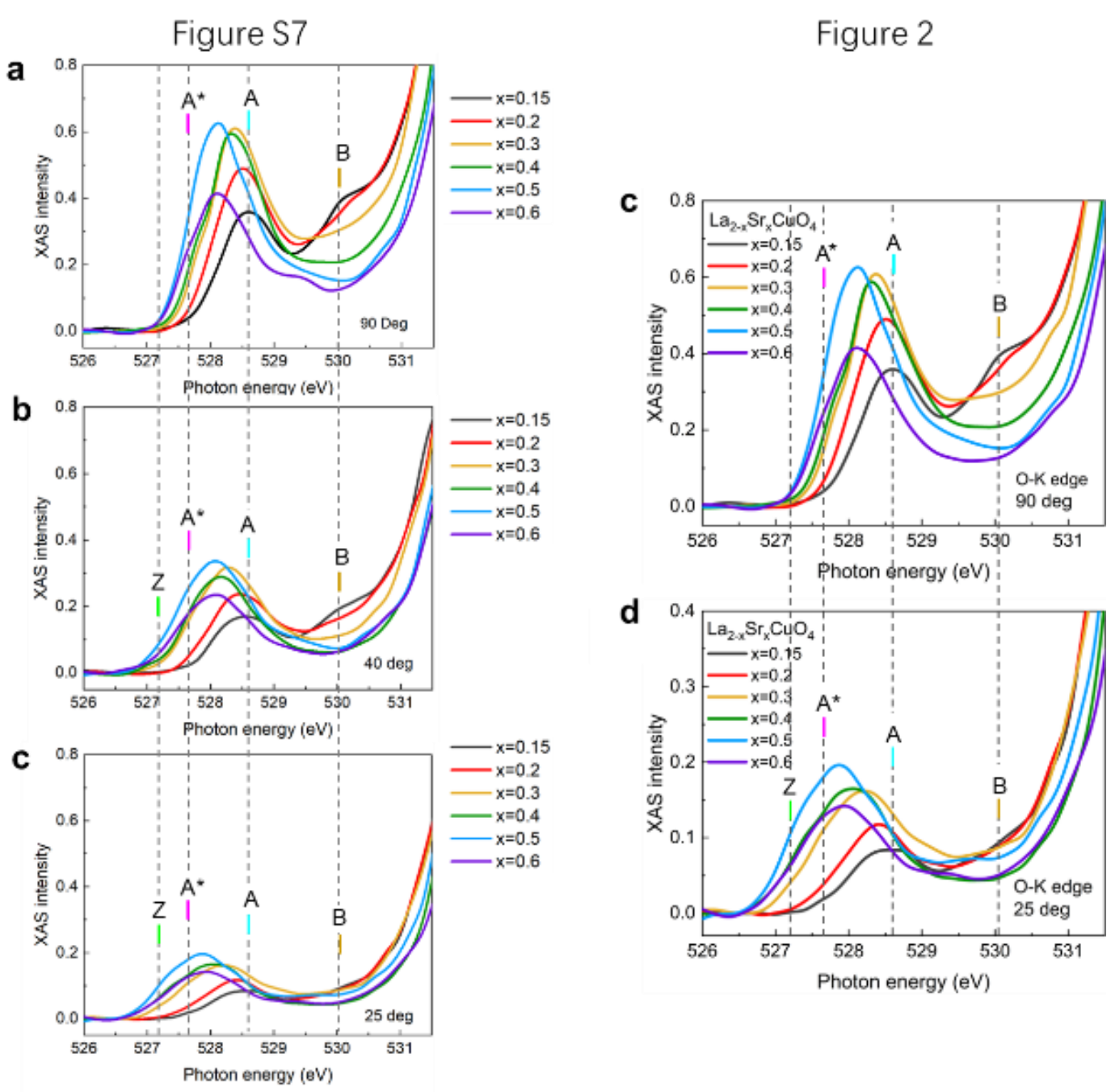

Fig. 2. (c) 90° (E//ab) and (d) 25° (E//c) incident XAS at the O *K*-edge for LSCO/LSAO samples of different doping concentration.

Fig. S7. The polarization-dependent O *K*-edge XAS spectra of LSCO ($0.15 \leq x \leq 0.6$) at incident angles of (a) 90°, (b) 40°, and (c) 25°.

2、Regarding the existence of peak A*, a weak shoulder (peak A*) is visible in the XAS spectrum near ~527.7 eV. This energy position corresponds to a reproducible curvature

feature in the second-derivative spectrum and remains consistent across different heavily overdoped LSCO samples (x>0.2). The second derivative is used only as a supplementary indicator and not as the sole basis for the peak assignment. Differential XAS was employed as a contrast-enhancement method to highlight subtle spectral changes that are difficult to discern directly in the raw XAS spectra. We also observed changes in the spectral line shape in the differential XAS spectra associated with the appearance and evolution of features Z, A*, and A. However, the differential XAS response is not uniquely determined by the emergence of new features, since it can also be influenced by peak shifts and variations in peak intensity. Taken together with the local line shape in the original spectrum, and the revised spectral fitting analysis, these observations provide consistent evidence that the shoulder at ~527.7 eV (peak A*) is a genuine spectral feature rather than an artifact arising from the analysis procedure.

Changes made to the Manuscript:

In the Supplementary Materials

A weak shoulder, denoted as feature A*, is observed in the XAS spectra at ~527.7 eV. The assignment of this feature A* is supported by several independent analyses. First, its energy position coincides with a curvature feature in the second-derivative XAS spectra (Fig. S9-S10). This feature remains stable across heavily overdoped LSCO samples with $x \geq 0.3$, indicating that it is not an artifact of a particular dataset or a statistical fluctuation. Here, the second-derivative analysis is used only as a supplementary diagnostic to assist in identifying subtle spectral structure and is not relied upon as the sole basis for the peak assignment.

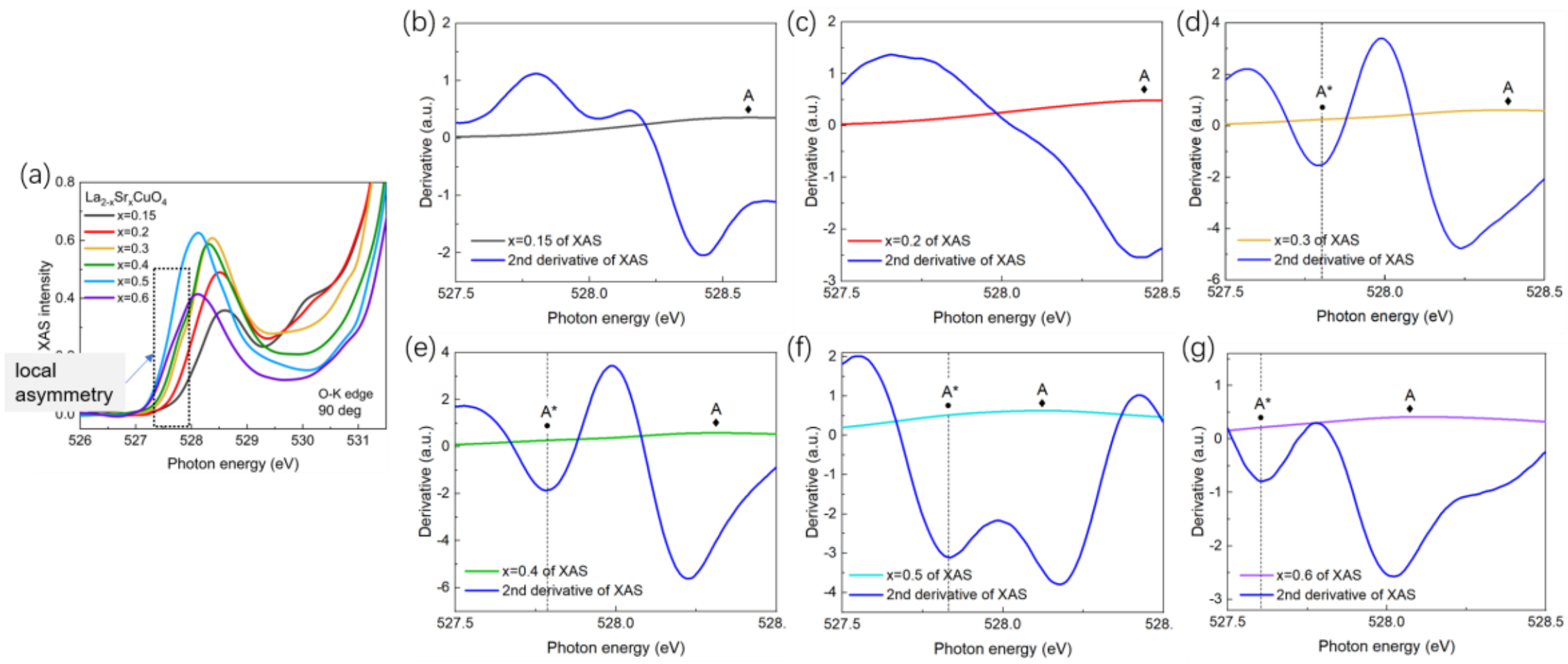

Fig. S9. The second derivative of the O K-edge XAS spectra measured at 90° incident angle.

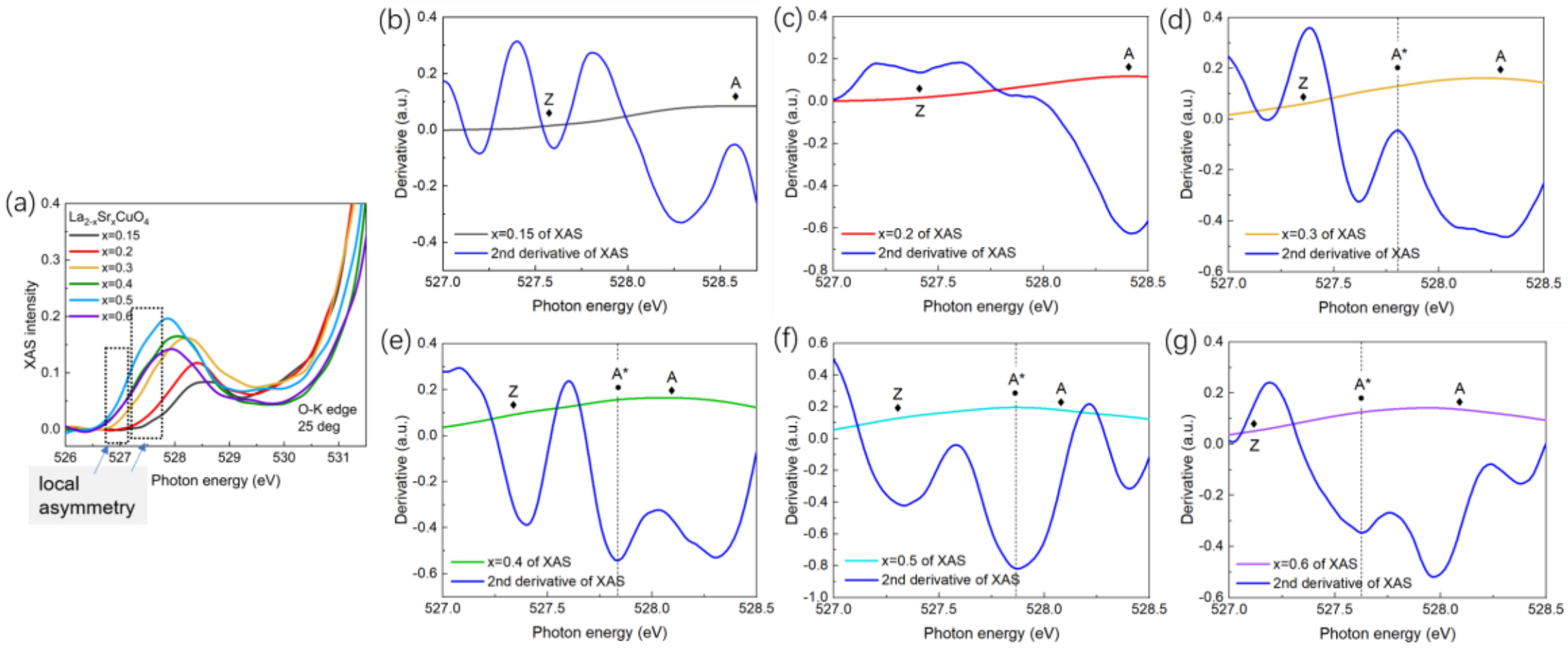

Fig. S10. The second derivative of the O K-edge XAS spectra measured at 25° incident angle.

In addition, differential XAS analysis was employed as a contrast-enhancement method to reveal weak spectral changes that are difficult to resolve directly in the raw XAS spectra (Fig. S11). The differential spectra show systematic line-shape variations associated with the emergence and evolution of features Z, A*, and A. We note, however, that the differential XAS response cannot by itself be interpreted as direct evidence for new spectral peaks, because it may also arise from shifts in peak position or changes in spectral intensity. Therefore, the differential XAS results were evaluated together with the local line shape of the original spectra and the outcomes of the fitting analysis. Taken together, the consistency of the second-derivative feature, the systematic behavior observed in the differential XAS spectra, the direct shoulder-like structure in the raw spectra, and the quantitative fitting results collectively support the interpretation that the shoulder near 527.7 eV (feature A*) represents a genuine spectral component rather than an artifact of the analysis procedure.

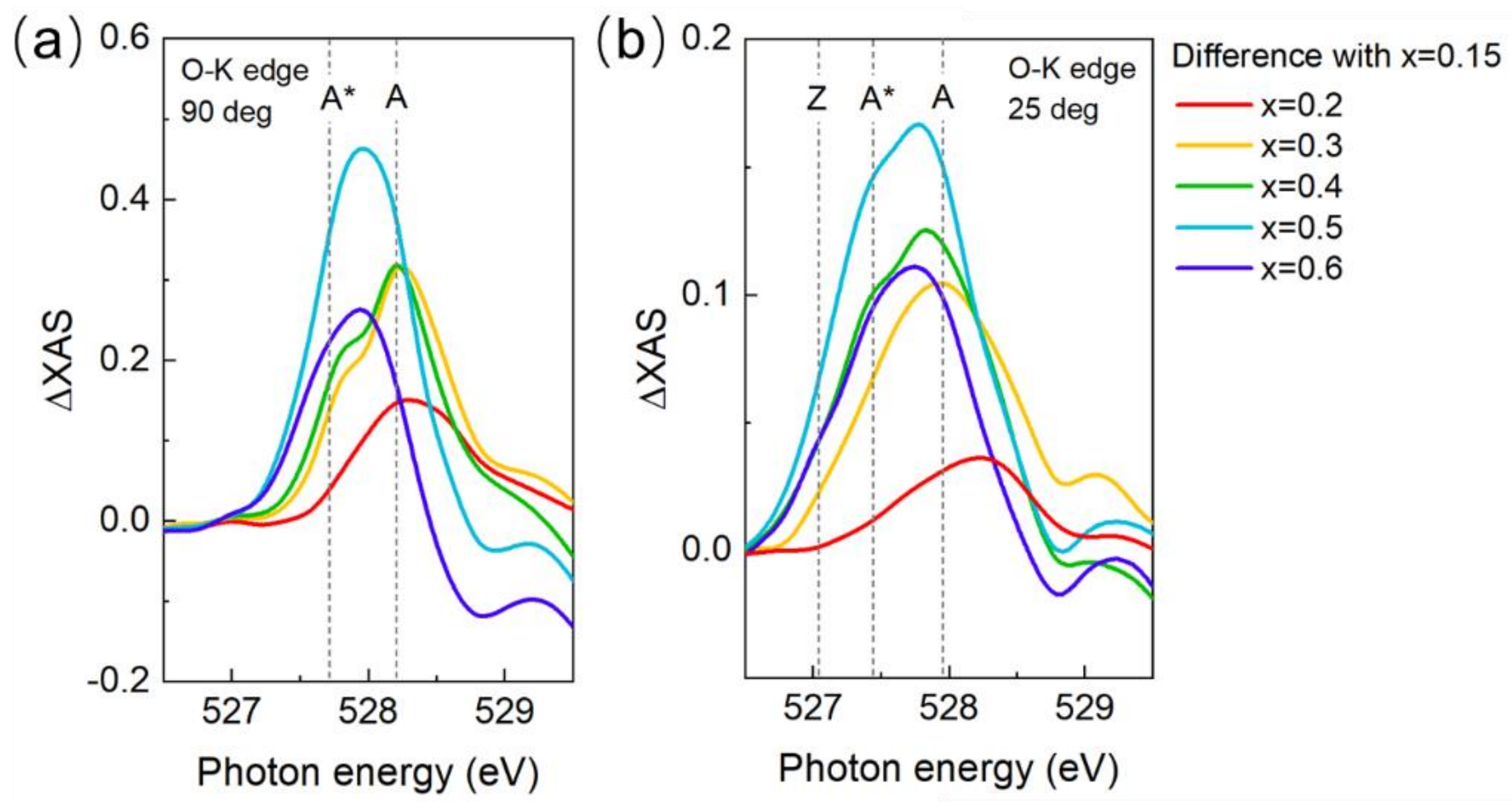

Fig. S11. The differential XAS spectra ΔXAS measured at 90° and 25° incident angles.

3. We thank the Reviewer for pointing out the insufficient description of the peak-fitting procedure. The Shirley background was adopted here as a phenomenological way to describe the slowly varying underlying tail beneath the pre-edge structure. We recognize that the Shirley function is not a uniquely prescribed physical background for XAS. Its role in the present analysis is therefore practical: it provides a consistent treatment of the non-flat baseline across the doping series and helps separate the weak shoulder feature from the broader underlying absorption. Importantly, the emergence of feature A* is visible already in the raw spectra and remains qualitatively robust under reasonable alternative background choices, indicating that the feature is not an artifact of the baseline model.

The choice of baseline does not influence the extracted peak positions. To ensure consistency across the dataset, the same baseline treatment was applied to all samples throughout the fitting procedure. In addition, the fitting was carried out under the constraint that the full width at half maximum (FWHM) of each component peak remained within a physically reasonable range. Finally, a comparative analysis of the fitted spectra was performed to qualitatively evaluate changes in peak intensity. Because this comparison was based on uniformly processed data and focused on relative trends in the fitted results, the conclusions are not sensitive to the specific baseline choice within reasonable limits.

Likewise, we used a Gaussian-Lorentz peak profile because the observed XAS line shape contains multiple broadening mechanisms. Therefore, a mixed profile provides a more realistic description than either a purely Gaussian or purely Lorentzian function.

In the O K-edge XAS fitting, the FWHM values were not allowed to vary freely, but were constrained within a physically reasonable range to ensure convergence toward physically meaningful linewidths during the fitting process. Based on prior reports on the line-shape characteristics and typical linewidths of O K-edge pre-edge features in cuprates [Physical Review Letters, 1991, 66(1): 104-107; Physical Review Letters, 1992, 68(16): 2543-2546; Nature Communications, 2023, 14(1): 529], the FWHM of Feature A was limited to 0.7–1.1 eV, consistent with the characteristic broadening of the ZRS-related pre-edge structure, while the FWHM of Feature A* was constrained to remain smaller than that of Feature A, consistent with its experimentally observed shoulder-like character.

We emphasize that the appearance of Features A and A* is not generated by the fitting procedure itself. Both features are already directly discernible in the raw XAS spectra of the heavily overdoped samples, and their approximate energy positions can be identified prior to any fitting analysis. Therefore, the fitting procedure was not used to define or create these features, but rather to quantify their spectral evolution and present the results in a systematic and reproducible manner.

We have incorporated the above details of the peak-fitting procedure into the revised manuscript in response to the reviewer’s suggestions, including the criteria for baseline selection, the determination of the A* peak position, the constraints imposed on the full width at half maximum (FWHM), and the choice of fitting model.

Changes made to the Manuscript:

In the Supplementary Materials

For the O K-edge XAS analysis, peak fitting was performed using a consistent procedure across the entire doping series. A Shirley-type background was adopted as a phenomenological baseline to describe the slowly varying absorption tail underlying the pre-edge region. The extracted peak positions were found to be insensitive to reasonable variations in the baseline model, and the same background treatment was applied to all spectra to ensure consistency. The spectra were fitted using a Gaussian–Lorentzian model, which accounts for the coexistence of multiple broadening mechanisms and provides a more realistic description than either a purely Gaussian or purely Lorentzian profile. During the fitting, the full width at half maximum (FWHM) of each component was constrained within physically reasonable limits to prevent unphysical broadening or narrowing and to ensure stable convergence of the fitting results.

4、In response to the Reviewers' suggestions, we have performed the same fitting procedure for the 25 deg XAS data. The photon energy position of A* across doping remains largely unchanged and consistent with that extracted from the 90 deg XAS spectra, indicating that feature A* is present in both the in-plane and out-of-plane orientations, as summarized in the table below. We find that the A peak exhibits a clear redshift with increasing doping concentration, in agreement with previous reports describing the shift of the ZRS band upon hole doping [Physical Review Letters, 1992, 68(16): 2543-2546; Phys Rev Lett, 1998, 80(23): 5192]. Feature A* shows only minor variations across the doping series. These results further support the robustness of feature A and indicate that its energy position is substantially less sensitive to hole doping than that of feature A*.

Changes made to the Manuscript:

At the Reviewer's request, the following sentence has been updated:

In the Supplementary Materials:

Through the above systematic fitting procedure, the energy positions of features A, Z, and A* in the O K-edge 90° and 25° XAS spectra were extracted and are summarized in Tables S1 and S2, respectively.

Table S1. The energy positions of features A and A* in the O K-edge 90 deg XAS spectra obtained from fitting analysis.

LSCO sample O-K edge 90 deg	Energy position of A* (eV)	Energy position of A (eV)
x=0.15	/	528.6
x=0.2	/	528.5
x=0.3	527.8	528.4
x=0.4	527.8	528.3
x=0.5	527.8	528.1
x=0.6	527.7	528.1

Table S2. The energy positions of features A, A* and Z in the O K-edge 25 deg XAS spectra obtained from fitting analysis.

LSCO sample O-K edge 25 deg	Energy position of Z (eV)	Energy position of A* (eV)	Energy position of A (eV)
x=0.15	527.5	/	528.6
x=0.2	527.4	/	528.4
x=0.3	527.2	527.8	528.4
x=0.4	527.2	527.8	528.2
x=0.5	527.2	527.8	528.1
x=0.6	527.1	527.7	528.1

In the revised version, we have included a more explicit description of the fitting procedure, including the number of features, fitting strategy, and parameter constraints.

Furthermore, the A* contribution is more pronounced in the 25° incidence geometry. What is the underlying physical mechanism for this angular dependence? A more rigorous analysis of the A* feature under different polarization conditions—particularly at 25°—could provide stronger evidence for electronic structure modification, yet the authors have not pursued this line of investigation.

1、We thank the reviewer for this important question. According to previous studies [Physical Review Letters, 1992, 68(16): 2543-2546], the out-of-plane orbital contribution in overdoped cuprates increases progressively with hole doping. In particular, the relative weight of the O2p_z component with respect to the in-plane O2$p_{x,y}$ component is non-negligible and evolves systematically with doping, reaching its largest value in the most heavily Sr-doped samples. Within this framework, our results suggest that in the heavily overdoped regime, the splitting of the original ZRS-derived band gives rise to the additional XAS feature A*. Compared with Feature A, A* exhibits a relatively enhanced out-of-plane contribution, consistent with the trend reported in the previous studies [Physical Review Letters, 1992, 68(16): 2543-2546], as reflected by the increased A/A* ratio in the 25° XAS spectra. However, the absolute intensity of both A* and A in the 25° incidence geometry is reduced relative to that observed at 90°.

2、We agree with the reviewer that a more systematic polarization-dependent analysis strengthens the evidence for electronic-structure reconstruction. Accordingly, in the revised manuscript we have added the fitting results for 25° XAS spectra, expanded the discussion of the polarization dependence of Feature A*, and clarified its physical interpretation in terms of overdoping-induced modification of the ZRS-derived electronic structure.

At a fixed polarization angle, the energy positions of features Z and A exhibit a gradual redshift with increasing hole doping. In contrast, when the XAS spectra are compared across different polarization geometries, the energy positions of these features remain nearly unchanged, indicating that the angular dependence primarily affects spectral

intensity rather than the underlying transition energies. In addition, as the spectra contain a larger out-of-plane contribution (25°), the absolute intensities of both A* and A decrease, consistent with the fact that these two features are predominantly associated with in-plane orbital states. Nevertheless, the relative intensity of A* with respect to A increases at 25° XAS. This behavior suggests that, although both features remain largely in-plane in character, feature A* contains a comparatively larger out-of-plane orbital admixture than Feature A in the heavily overdoped regime, possibly due to the increasing occupation or hybridization of out-of-plane orbitals by doped holes.

Changes made to the Manuscript:

At the Reviewer's request, the following sentence has been updated:

In the main text:
Similar fitting results obtained from the out-of-plane O K-edge XAS spectra are presented in Fig. S12, showing that Feature A* also emerges for $x > 0.2$.

In the Supplementary Materials:

To identify feature A*, spectral fitting was performed for both the in-plane and out-of-plane O K-edge XAS spectra. The corresponding results are presented in Fig. 2(e) of the main text and Fig. S12, respectively. In addition, the energy positions of features A, Z, and A* in the out-of-plane O K-edge XAS spectra were extracted, as shown in Tables S2. The position of Comparison of the in-plane and out-of-plane O K-edge XAS spectra shows that, although the absolute intensities of both A* and A decrease as the out-of-plane contribution increases, the relative intensity of A* with respect to A becomes enhanced at 25°, suggesting that A* retains predominantly in-plane character but acquires a comparatively stronger out-of-plane orbital admixture in the heavily doped regime, possibly due to increased occupation of, or hybridization with, out-of-plane orbitals by doped holes [Physical Review Letters, 1992, 68(16): 2543-2546].

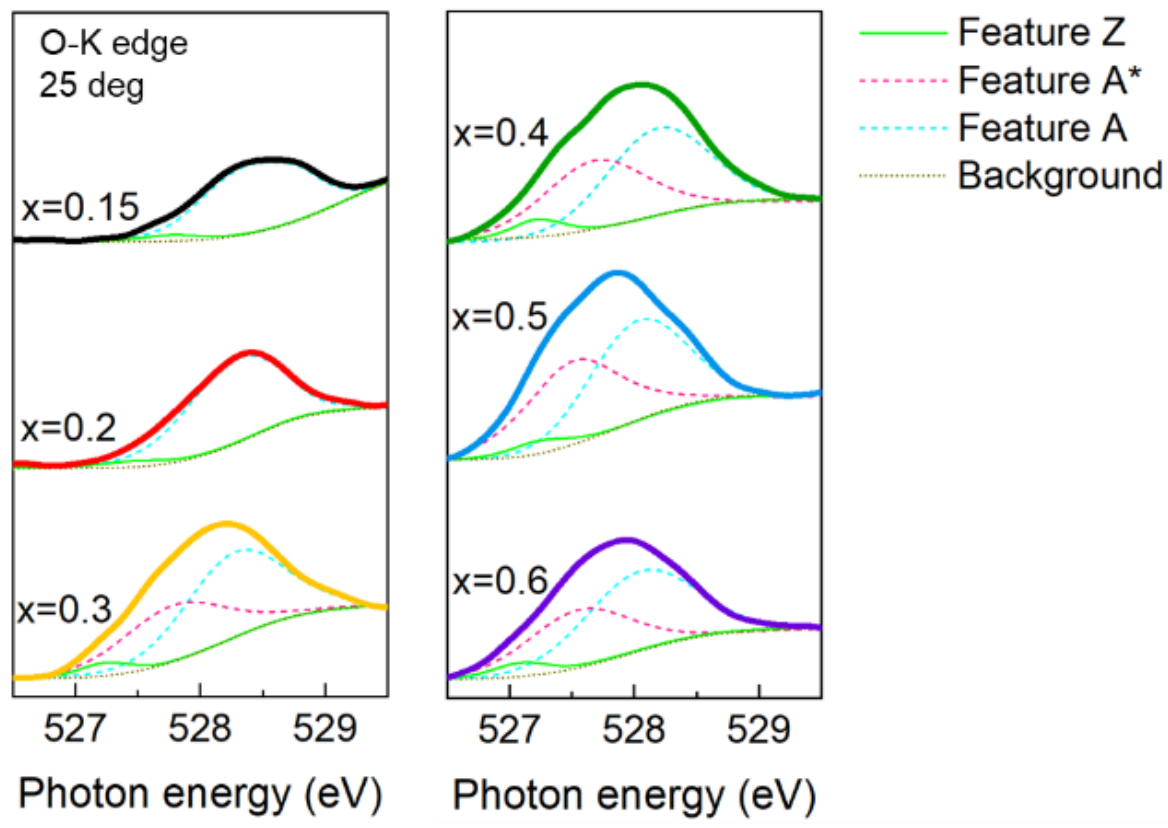

Fig. S12. The fitting results of Fig.2d (in the main text) for identify the feature Z, A^* and A.

Table S2. The energy positions of features A, A* and Z in the O K-edge 25 deg XAS spectra obtained from fitting analysis.

LSCO sample O-K edge 25 deg	Energy position of Z (eV)	Energy position of A* (eV)	Energy position of A (eV)
x=0.15	527.5	/	528.6
x=0.2	527.4	/	528.4
x=0.3	527.2	527.8	528.4
x=0.4	527.2	527.8	528.2
x=0.5	527.2	527.8	528.1
x=0.6	527.1	527.7	528.1

The authors claimed: A pronounced drop in spectral intensity is observed at x = 0.6, which may be attributed to a transfer of spectral weight to higher absorption energies" in the main text. However, this is not true, since the transfer of spectral weight is also shown for x=0.5 without the drop in spectral intensity.

We thank the Reviewer for this careful observation and we agree with the Reviewer's assessment. Since spectral-weight redistribution is already visible at x=0.5, the additional suppression of the O K-edge intensity at x=0.6 cannot be attributed to higher-energy spectral-weight transfer alone. We have therefore revised the manuscript to present a more balanced interpretation of this behavior.

From Fig. 4a, the low-temperature resistance curve of x=0.6 sample exhibits an insulating tendency, suggesting that a local metal-to-insulator transition may occur between x = 0.5 and x = 0.6. Accordingly, the pronounced reduction in spectral intensity observed at x = 0.6 may arise from both spectral-weight transfer to higher absorption energies and the onset of a local metal-to-insulator transition. Therefore, the substantial loss of spectral weight in the x = 0.6 sample is likely the result of the combined influence of these two effects.

Most importantly, this interpretation does not affect the central conclusions of the present study. The observation of electronic-structure reconstruction in heavily overdoped LSCO (x > 0.2) remains fully supported by the experimental results.

Changes made to the Manuscript:

At the Reviewer's request, the following sentence has been updated:

An abrupt drop in XAS intensity is observed at the doping level of x = 0.6. One possible reason is the transfer of spectral weight to higher absorption energies (Fig. S3). In addition, the low-temperature resistivity curve of the x = 0.6 sample exhibits an insulating tendency (Fig. 4a), suggesting that a local metal-to-insulator transition may also contribute to the pronounced suppression of XAS intensity at this doping level.

I wonder why the Cu L-edge XAS are not involved in the investigation, which certainly helps to understand the electronic structure of LSCO.

We thank the Reviewer for this valuable suggestion. As recommended, we have supplemented the revised manuscript with Cu L-edge XAS measurements, which provide additional information on the Cu-related unoccupied states and offer a complementary perspective on the electronic-structure evolution of LSCO.

The XAS spectra at the Cu L_3 edge display features C (~931.2 eV) and D (~932.6 eV), and E (~934.6 eV) across all doping levels of LSCO. Feature C is attributed to the transition from the $2p^63d^9$ to $2p^53d^{10}$ state, while feature D arises from the transition from $2p^63d^9L$ to $2p^5d^{10}L$[Phys Rev Lett 121, 067602 (2018); PNAS 116, 12156 (2019)]. In addition, the shoulder feature D represents a signature of the ZRS state whose spectral weight is proportional to the number of holes introduced to the CuO_2 planes [Physics Letters A 127, 285 (1988)]. The doping dependence of Feature D is shown in the revised manuscript and exhibits a systematic increase in intensity with increasing hole concentration. Notably, the intensity of feature D at x=0.6 exhibits an abrupt suppression, resembling the behaviour observed in the O K-edge spectra and potentially reflecting a related modification of the electronic state at this doping level.

We emphasize that the central conclusions of the present study are derived primarily from the analysis of the O K-edge XAS data. The Cu L_3-edge measurements serve as an independent and complementary probe that is broadly consistent with the electronic-structure evolution inferred from the O K-edge results. Accordingly, these data have been included in the Supplementary Information of the revised manuscript.

Changes made to the Manuscript:

At the Reviewer's request, the following sentence has been updated:

In the main text:
Meanwhile, the polarization-dependent XAS spectra at the Cu L_3-edge across all doping levels of LSCO, as shown in Fig. S13. However, the most significant doping-induced changes are expected to occur in the pre-edge region of the O K-edge spectra.
In the Supplementary Materials:
The polarization-dependent XAS spectra at the Cu L_3 edge display features C (~931.2 eV) and D (~932.6 eV) across all doping levels of LSCO, as shown in Fig. S13. Feature C is attributed to the transition from the $2p^63d^9$ to $2p^53d^{10}$ state, while feature D arises from the transition from $2p^63d^9L$ to $2p^5d^{10}L$ [Phys Rev Lett 121, 067602 (2018); PNAS 116, 12156 (2019)]. In addition, the shoulder feature D represents a signature of the ZRS state whose spectral weight is proportional to the number of holes introduced to the CuO_2 planes [Spectroscopy of Complex Oxide Interfaces (Springer International Publishing, Cham, 2018); Physics Letters A 127, 285 (1988). Fig.S13(d) plots the intensity evolution of feature D at the Cu L_3 edge, which exhibits a systematic increase in XAS intensity with increasing doping.

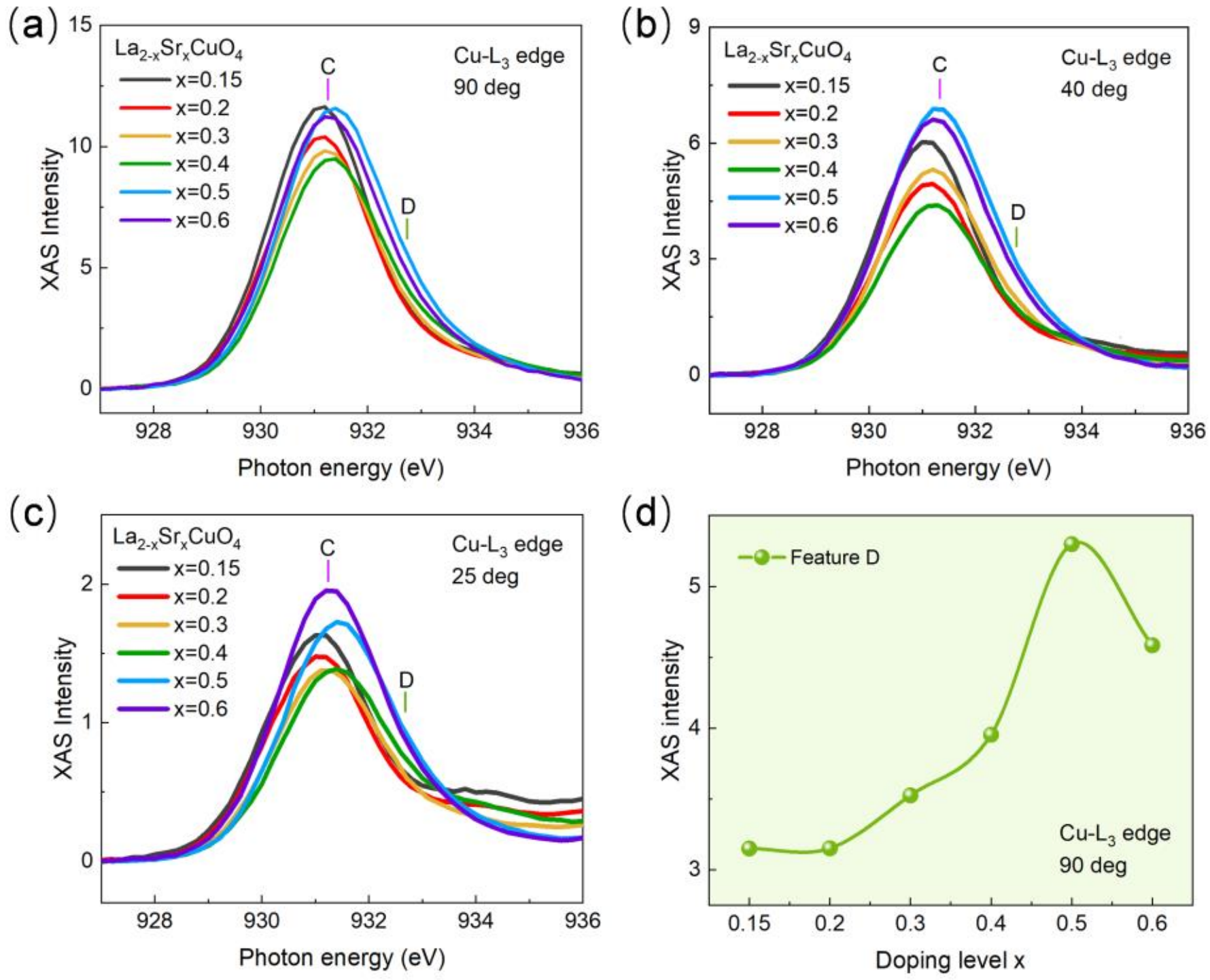

Comment 2: Optical spectroscopic ellipsometry results

The data analysis in Figure 3(d) exhibits similar problems to the XAS fitting procedure. Key parameters including precise peak positions, full width at half maximum (FWHM) values, and fitting constraints remain unspecified. The absence of clear criteria for the Drude-Lorentz fitting methodology undermines the reliability of the extracted spectral features.

1、We thank the Reviewer for reminding us for the inadequately description about peak fitting procedure in optical spectroscopic ellipsometry.

Peaks α and α* in the optical conductivity spectrum are closely spaced, distinct such that their individual contributions cannot be directly resolved from the raw spectra. Moreover, the spectral features in the 1.0–2.0 eV range progressively broaden and become strongly asymmetric with increasing doping. We therefore first employed differential optical conductivity, $\Delta\sigma_1$, as a contrast-enhancement method, which revealed the presence of these peaks and their approximate energy positions. We then carried out a detailed fitting analysis of the optical conductivity spectra to more precisely determine the energy positions of the individual characteristic peaks.

From the differential optical conductivity $\Delta\sigma_1$ spectra and the subsequent fitting analysis, we extracted the peak energies of features α and α*, located at ~1.5 eV and ~1.1 eV, respectively.

Changes made to the Manuscript:

At the Reviewer's request, the following sentence has been updated:

In the Supplementary Materials:

The differential optical conductivity spectra, $\Delta\sigma_1$, provide a more sensitive means of identifying subtle spectral features and estimating their approximate energy positions. Two distinct spectral signatures associated with features α and α* are observed in the $\Delta\sigma_1$ spectra (Fig. S18), providing independent evidence for the presence of these features in the optical conductivity, σ_1, spectrum.

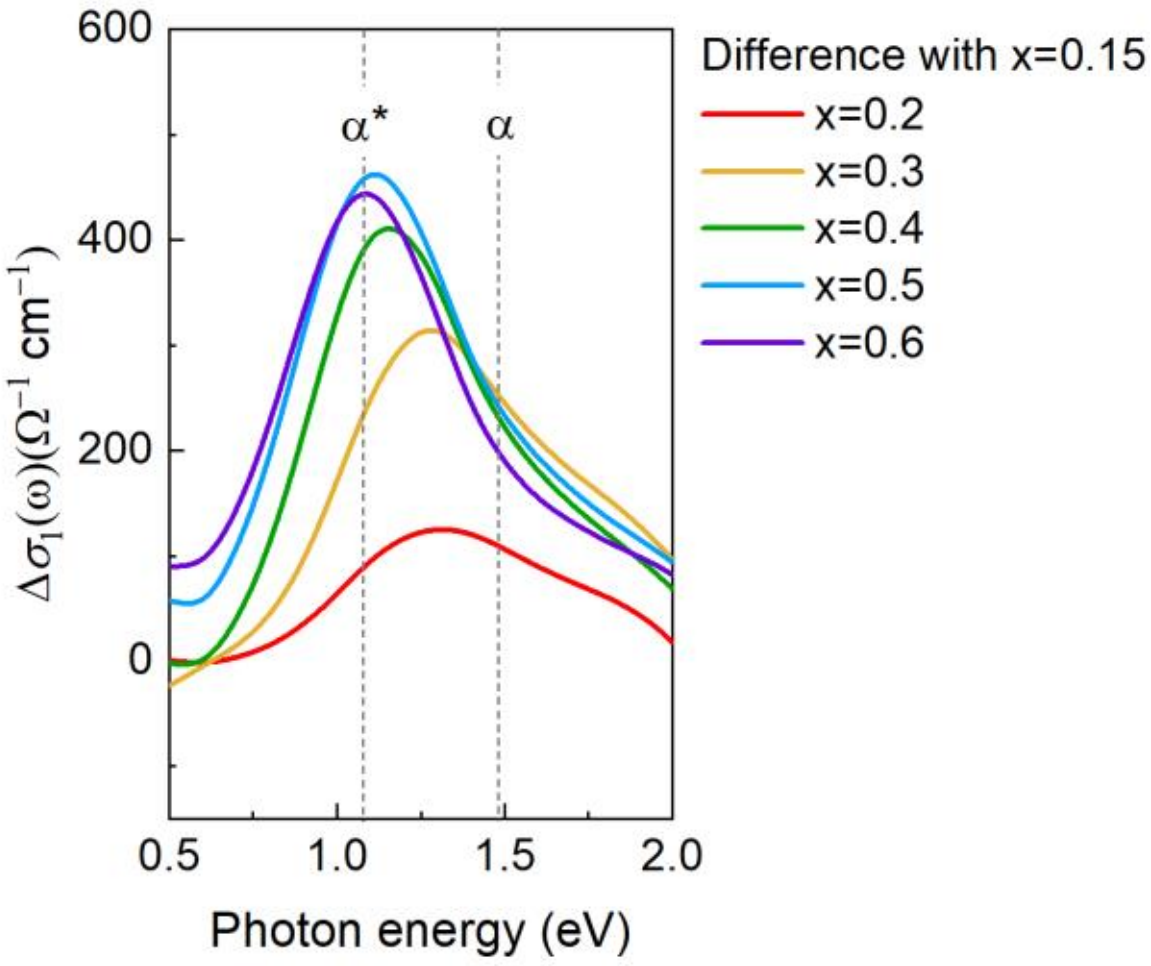

Fig. S18. The differential optical conductivity $\Delta\sigma_1$ spectra from optical spectroscopic ellipsometry measurements.

On this basis, a detailed fitting analysis of the optical conductivity spectra was subsequently performed in order to determine the feature energies more accurately. The fitting results are plotted in Fig. 3d in the main text.

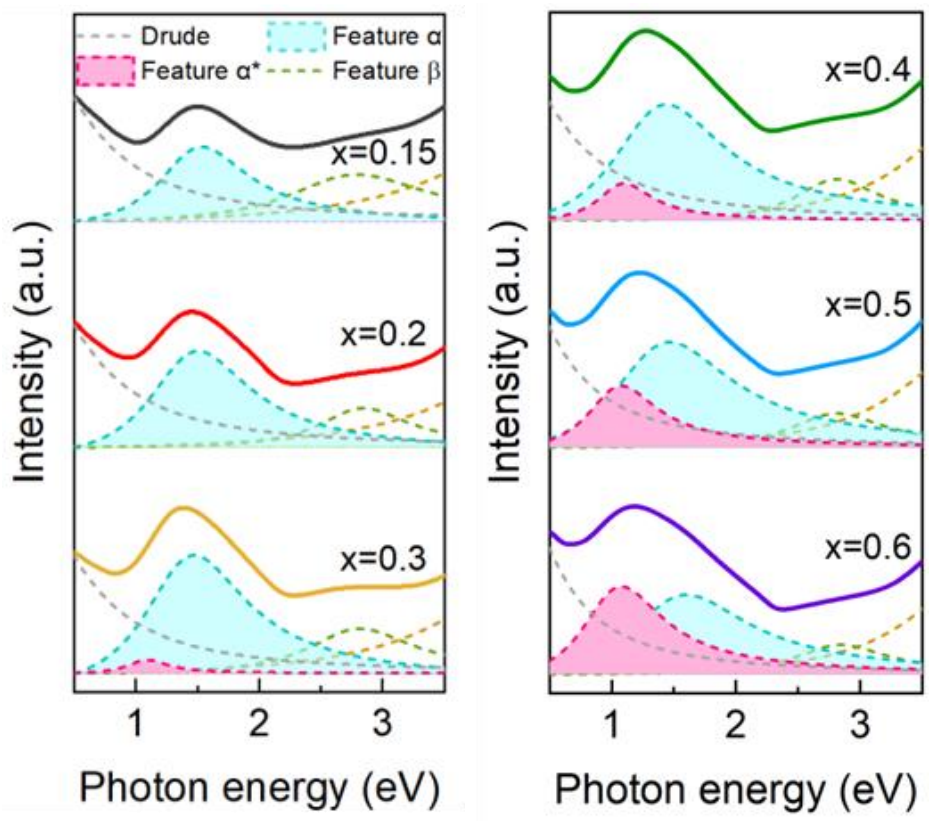

Through this combined analysis of $\Delta\sigma_1(\omega)$ and the fitted $\sigma_1(\omega)$ spectra, the energies of features α and α^* were extracted to be ~1.5 eV and ~1.1 eV, respectively. The appearance of the α^* feature provides further evidence for the emergence of a new low-energy excitation channel in the heavily overdoped regime and supports the conclusion of doping-induced electronic-structure reconstruction in LSCO. Using this systematic fitting procedure, the energy positions of features α and α^* were extracted for all doping concentrations and are summarized in Table S3.

Table S3. The energy positions of features α and α* in the $\sigma_1(\omega)$ spectra obtained from fitting analysis.

LSCO sample Optical conductivity	Energy position of α (eV)	Energy position of α* (eV)
x=0.15	1.5	/
x=0.2	1.5	/
x=0.3	1.5	1.1
x=0.4	1.5	1.1
x=0.5	1.5	1.1
x=0.6	1.5	1.1

2、We thank the Reviewer for this valuable comment regarding the choice and implementation of the Drude-Lorentz fitting methodology.

The dielectric functions of metals and semiconductors with high carrier concentrations exhibit strong free carrier absorption characteristics, which can be represented by the Drude model. The carrier concentration and mobility in metals/semiconductors can be estimated using the Drude model for analysis. However, the dielectric response generally contains contributions from both free carriers and bound electronic excitations. Therefore, the dielectric function of a metal/semiconductor is expressed as

$$\tilde{\varepsilon}(\omega) = \varepsilon_1(\omega) + i\varepsilon_2(\omega)$$

$$\tilde{\varepsilon}(\omega) = \tilde{\varepsilon}_{free}(\omega) + \tilde{\varepsilon}_{bound}(\omega)$$

The free electron component as represented by $\tilde{\varepsilon}_{free}$ is modelled by the Drude model while the bound electrons as represented by $\tilde{\varepsilon}_{bound}$ by the Lorentz model. The Lorentz component accounts for interband and bounds-state excitations and enables a physically meaningful description of the dielectric response over a broad spectral range.

Accordingly, the Drude-Lorentz formalism has been widely employed to model the optical response of semiconducting and correlated-electron systems that exhibit both free-carrier and bound-electron contributions. The model provides an accurate and physically motivated representation of the dielectric function across a wide energy range and has been successfully applied in numerous optical spectroscopy studies [Physical Review B 71, 085416 (2005); Applied Physics Reviews 12, 011403 (2025)].

Changes made to the Manuscript:

At the Reviewer's request, the following sentence has been updated:

In the Supplementary Materials:

Drude-Lorentz Model

The dielectric functions of metals and semiconductors with high carrier concentrations exhibit strong free carrier absorption characteristics, which can be represented by the Drude model. The carrier concentration and mobility in metals/semiconductors can be estimated using the Drude model for analysis. However, the measured dielectric response

contains contributions from both free carriers and bound electronic excitations. Therefore, the dielectric function of a metal/semiconductor is expressed as

$$\tilde{\varepsilon}(\omega) = \varepsilon_1(\omega) + \varepsilon_2(\omega) \quad \text{(S10)}$$

$$\tilde{\varepsilon}(\omega) = \tilde{\varepsilon}_{free}(\omega) + \tilde{\varepsilon}_{bound}(\omega) \quad \text{(S11)}$$

The free electron component as represented by $\tilde{\varepsilon}_{free}$ is modelled using the Drude model while the bound-electrons component represented by $\tilde{\varepsilon}_{bound}$ is modelled using Lorentz oscillators. Together, these terms provide a physically motivated description of the dielectric response and enable quantitative modeling of the optical spectra.

The Drude–Lorentz model has been extensively applied to semiconducting and correlated-electron systems that contain both free-carrier and bound-electron contribution. Its flexibility and physical basis make it particularly suitable for describing dielectric functions over a broad spectral range and for extracting characteristic optical excitations through curve fitting [*Physical Review B* 71, 085416 (2005); Applied Physics Reviews 12, 011403 (2025)].

3. We thank the Reviewer for this helpful observation. Similar to the XAS analysis, the optical-conductivity fitting in Fig. 3(d) was performed with explicit parameter constraints to avoid over-parameterization and nonphysical solutions. The fitting range was limited to 0.5–3.5 eV, and the Lorentz-oscillator linewidths were constrained so that the FWHM values remained below 1.5 eV, thereby preventing individual peaks from becoming artificially broad and absorbing unrelated spectral intensity. Furthermore, the fitting model was deliberately kept minimal: for x=0.15-0.2, the spectra were described by one Drude term and three Lorentz oscillators, whereas for x=0.3-0.6 the emergence of the additional low-energy feature required one Drude term and four Lorentz oscillators. These constraints were introduced to ensure numerical stability, physically meaningful linewidths, and a consistent fitting framework across the entire doping series.

Changes made to the Manuscript:

At the Reviewer's request, we have clarified these fitting conditions explicitly in the revised manuscript and the following has been updated:

In the Supplementary Materials:

For the optical conductivity analysis, peak fitting was performed using a consistent procedure across the entire doping series. Regarding the optical conductivity analysis and peak fitting, no additional baseline was introduced in the fitting procedure. This is because the optical conductivity spectra in the 0.5–3.5 eV energy range do not exhibit a significant background variation, allowing the spectral features to be analyzed directly without the need for an additional baseline correction. The same treatment has been applied to all spectra to ensure consistency.

During the fitting, the full width at half maximum (FWHM) of each component was constrained within physically reasonable limits to prevent unphysical broadening or

narrowing and to ensure stable convergence of the fitting results. To avoid over-parameterization, the fitting model was kept to the minimum required to reproduce the experimental spectra. Specifically, the spectra for x=0.15-0.2 were adequately described by one Drude term and three Lorentz oscillators, whereas for x=0.3-0.6 an additional Lorentz component was required to account for the emergence of the extra low-energy feature. This fitting strategy provides a numerically stable and physically consistent description of the optical conductivity spectra while enabling direct comparison of spectral evolution across the full doping series.

A significant discrepancy emerges when comparing the doping-dependent intensity variations between optical and XAS measurements: In the optical conductivity spectra, the α* feature surpasses the α feature in intensity with increasing doping (x). Conversely, in the XAS spectra, the A* feature shows relatively weaker enhancement compared to feature A. This contradictory behavior raises critical questions about the interpretation: Does this inconsistency reflect fundamentally different physical origins of the α* and A* features? Could methodological artifacts in the fitting procedures account for the divergent trends? What is the theoretical basis for expecting parallel evolution between these spectroscopically distinct features? Besides, is there any relationship between the energy difference between that of A and A*, and α* and α? The authors must address whether this discrepancy indicates limitations in the proposed checkerboard-type ZR configuration model or reveals more complex electronic reconstruction mechanisms.

We thank the Reviewer for this insightful comment. We clarify that the optical feature α* and the XAS feature A* are not expected to exhibit identical intensity evolution because the two techniques probe different physical quantities. O K-edge XAS probes unoccupied electronic states above the Fermi level, whereas optical conductivity measures transition energies between occupied and unoccupied states.

Therefore, α* and A* are complementary but not equivalent observables. The difference in their doping evolution does not indicate inconsistency, but rather reflects the different physical processes involved. In the revised manuscript, we have clarified this distinction and avoided implying a one-to-one correspondence between these features.

XAS and optical conductivity probe different excitation processes, although the A/A* and α/α* features are related to the same underlying electronic structure evolution. According to the energy band structure, the feature A in XAS spectra presents the transition from core-level to ZR2 state, while feature A* in XAS spectra presents the transition from core-level to unoccupied ZR1 state. Meanwhile, the feature α in optical conductivity spectra contains contributions from transitions between the lower Hubbard band (LHB) and the ZR2 state, as well as transitions from occupied ZR1 states to the upper Hubbard band (UHB), whereas feature *α** is primarily associated with transitions from the LHB into the unoccupied ZR1 state. Within this interpretation, the energy separation between A and A* is expected to reflect the same underlying ZR1–ZR2 energy splitting that gives rise to the

separation between α and α*. The experimentally observed energy difference of approximately 0.4 eV is consistent with this picture

We further note that the XAS and OSE measurements were conducted under different incident geometries: the XAS data were collected at 25°, 40°, and 90°, while the OSE measurements were performed at 30° relative to the sample plane. Since the incident angle determines the relative sensitivity to in-plane and out-of-plane orbital components, the spectral features A, A*, α, and α* may contain different proportions of these orbital contributions depending on the experimental geometry. For this reason, a strict quantitative one-to-one correspondence between the absolute spectral weights extracted from XAS and optical conductivity is not necessarily expected. Nevertheless, both techniques consistently capture the same doping-dependent evolution of the electronic structure, providing mutually supportive evidence for the electronic-structure reconstruction identified in heavily overdoped LSCO and reinforcing the central conclusions of the present work.

XAS→

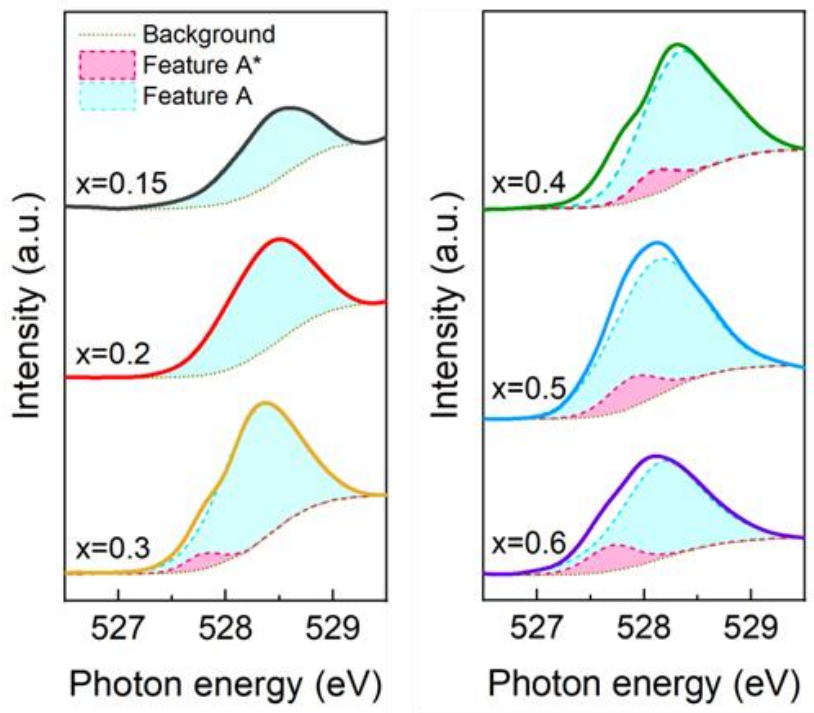

OSE→

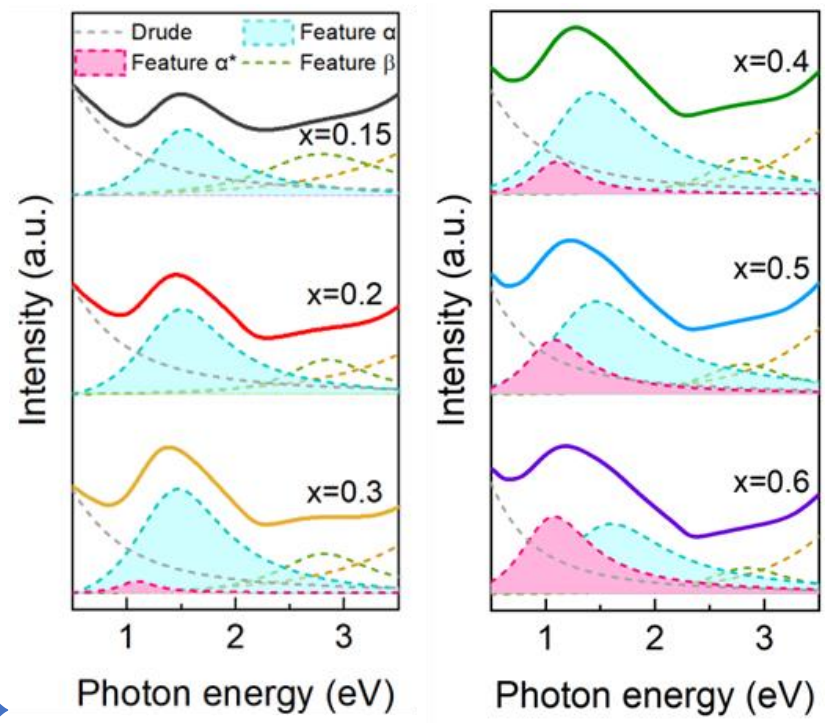

Changes made to the Manuscript:

In the main text:

A point that deserves clarification is that the O K-edge XAS and optical conductivity measurements do not probe the same physical quantity, and therefore their spectral weights should not be expected to exhibit a strict one-to-one correspondence.

In the Supplementary Materials:

(4) The difference between O K-edge XAS and optical conductivity results

A point that deserves clarification is that the O K-edge XAS and optical conductivity measurements do not probe the same physical quantity, and therefore their spectral weights (intensity) should not be expected to exhibit a strict one-to-one correspondence. O K-edge XAS measures transitions from the O 1s core level into unoccupied O 2p-derived states above the Fermi level and is thus primarily sensitive to the distribution of unoccupied electronic states and their orbital character (Figs. 3e-f). By contrast, optical conductivity probes interband excitations between occupied and unoccupied states and therefore reflects the transition matrix elements and joint evolution of both sides of the electronic structure (Figs. 3e-f). Consequently, although the XAS features A and A* and the optical features α and α* originate from the same underlying doping-induced reconstruction of the electronic structure, their absolute intensities and doping dependences are not required to evolve identically. In addition, XAS and optical measurements were carried out under different incident-light angles, which introduce different sensitivities to in-plane and out-of-plane orbital components. Accordingly, the most physically meaningful comparison

between the two techniques lies not in an exact correspondence of spectral weight, but rather in their consistent identification of the same doping-driven electronic-structure evolution and the emergence of additional electronic states in the heavily overdoped regime.

Comment 3: Critical issue regarding oxygen defects in samples

The authors have insufficiently addressed the issue of oxygen defects in the main text, relegating crucial descriptions to the supplementary materials. This is particularly problematic for O K-edge studies, where oxygen vacancies exert significant and unavoidable influences on spectral line shapes. While the supplementary materials mention oxygen defect formation for $x > 0.4$, the authors provide no experimental evidence to conclusively demonstrate the absence of such defects at lower doping levels ($x < 0.4$). The authors' categorical claim that the 526-530 eV absorption region remains unaffected by oxygen vacancies is scientifically untenable. Existing literature consistently demonstrates that oxygen defects can substantially modify the relative peak intensities between spectral features, energy positions of characteristic absorption edges and the overall spectral line shape and background contribution. Without rigorous exclusion of oxygen vacancy effects, the proposed electronic structure reorganization and checkerboard-type ZR configuration hypothesis remains speculative. The attribution of spectral changes solely to doping-induced electronic reconstruction requires substantially stronger experimental validation. This fundamental methodological issue must be conclusively addressed to establish the scientific robustness of the proposed model.

We thank the Reviewer for raising this important point. We agree that oxygen nonstoichiometry can influence O K-edge line shapes, including relative peak intensities, edge positions, spectral broadening, and background contributions. We also agree that our previous statement that oxygen defects have no discernible influence in the 526-531 eV range was too categorical. We have therefore revised the manuscript and Supplementary Information to avoid this overstatement. In the revised version, we do not claim that oxygen vacancies can be completely excluded. Instead, we clarify that oxygen vacancies may contribute to spectral broadening and background variations, especially in the highest-doped samples, but they are unlikely to be the primary origin of the newly observed A* and α* features.

Our conclusion is based on several mutually consistent observations. First, all LSCO films were grown and post-annealed under oxygen-containing conditions, and the XRD, RSM, and STEM characterizations show high crystalline quality, coherent epitaxy, and no detectable secondary phases across the doping series. Although these structural data cannot by themselves prove the complete absence of oxygen vacancies, they indicate that the observed spectroscopic evolution is not associated with gross structural degradation or phase separation.

Second, the doping onset of the new spectroscopic features is clearly different from the onset of the signatures that we associate with oxygen deficiency. The A* feature in the O

K-edge XAS and the α* feature in the optical conductivity both emerge already for $x > 0.2$, particularly in the $x = 0.3$ sample. By contrast, the transport and lattice signatures most consistent with oxygen deficiency in our samples, namely the resistivity upturn and the reversal or sharp decrease of the out-of-plane lattice parameter, become evident only at higher Sr concentrations, mainly for $x > 0.4$. This mismatch in onset strongly argues against oxygen vacancies being the primary cause of the emergence of A* and α*. Oxygen vacancies may affect the line shape and intensity redistribution in the highest-doped samples, but they do not naturally explain why the new features already appear at lower overdoping levels where the oxygen-deficiency-related structural and transport signatures are not dominant.

Third, the new feature is observed by two independent spectroscopic probes. The A* feature is detected in O K-edge XAS, whereas the α* feature is obtained from optical spectroscopic ellipsometry through the optical conductivity. These probes are sensitive to different aspects of the electronic structure: XAS probes unoccupied O-derived states, while optical conductivity reflects interband transitions and the electrodynamic response of the sample. Consequently, an oxygen-vacancy-induced modification of the O K-edge line shape alone would not naturally account for the correlated emergence of α^* in the optical conductivity, the evolution of the dielectric response, and the accompanying changes in transport behavior observed within the same doping regime.

Fourth, the saturation and anomalous evolution of low-energy O K-edge spectral weight around hole doping $x \sim 0.21$ have been reported previously in overdoped cuprates, independent of our samples. This literature evidence supports the view that the electronic structure of overdoped cuprates undergoes an intrinsic doping-driven change near this doping regime. Our observation of A* and α* is therefore consistent with a broader body of experimental evidence for electronic reconstruction in overdoped cuprates, rather than being uniquely attributable to oxygen defects in the present films.

Finally, our DQMC calculations based on the three-orbital Emery model do not include oxygen vacancies or extrinsic defect states. Nevertheless, the calculations reproduce the essential trend toward ZRS instability and the emergence of additional O-derived spectral weight at high hole density. This theoretical result does not prove the absence of oxygen vacancies in the experimental samples; however, it demonstrates that oxygen vacancies are not required to generate the key phenomenology discussed in this work.

We have revised the main text and Supplementary Information accordingly. We now explicitly state that oxygen nonstoichiometry is a possible secondary contribution, especially in the highest-doped samples, but that the onset, cross-probe consistency, literature agreement, and vacancy-free theoretical reproduction of the spectral reconstruction indicate that the A*/α* features primarily reflect a doping-induced electronic-structure reconstruction rather than an oxygen-vacancy-dominated effect.

Changes made to the Manuscript:

At the Reviewer's request, the following sentence has been updated:

In the Supplementary Materials:

Because oxygen nonstoichiometry can influence O K-edge spectral line shapes in complex oxides, the possibility that the newly observed A* and α* features are primarily defect-related was considered. Oxygen-vacancy-related effects may contribute to spectral broadening and background variations, particularly in the most highly doped LSCO films. However, A* in the O K-edge XAS and α* in the optical conductivity emerge already for $x > 0.2$, whereas the transport and lattice signatures most consistent with oxygen deficiency become evident mainly at higher doping levels. This mismatch in onset, together with the concurrent appearance of A* and α* in two independent spectroscopic probes and the vacancy-free DQMC results, indicates that oxygen vacancies are unlikely to be the primary origin of the new spectral features. Accordingly, the A* and α* features are interpreted primarily as manifestations of a doping-induced reconstruction of Zhang–Rice-related electronic states, while oxygen nonstoichiometry may provide a secondary contribution, particularly in the most heavily doped films.

Reviewer #3 (Remarks to the Author):

In this manuscript, the authors systematically investigate the electronic structure evolution of heavily overdoped LSCO thin films in the regime of x = 0.15–0.60. This is achieved through a combination of high-resolution X-ray absorption spectroscopy (XAS) , optical spectroscopic ellipsometry (OSE), and determinant quantum Monte Carlo (DQMC) simulations based on the three-orbital Emery model. The authors successfully observe spectroscopic evidence of Zhang-Rice singlet (ZRS) breakdown, characterized by the emergence of A* and α* peaks in the overdoped region. Furthermore, they creatively propose a "checkerboard-type" physical picture for the electronic spatial configuration. The experimental data is robust, and the theoretical physical picture is clear. This work is of significant importance for advancing our understanding of electron correlation effects and the superconducting mechanisms in heavily overdoped cuprates. Therefore, I recommend the manuscript for acceptance after minor revisions to address the following specific points.

We sincerely thank the Reviewer for the positive and encouraging assessment of our work. We greatly appreciate the Reviewer’s recognition of the quality of the experimental data, the clarity of the theoretical interpretation, and the potential significance of this study for understanding electron-correlation effects and superconductivity in heavily overdoped cuprates. We are also grateful for the Reviewer’s recommendation for acceptance after minor revisions. In response to the comments provided, we have carefully revised the manuscript and addressed all points raised by the Reviewer to further improve the clarity and rigor of the presentation.

1.Many studies indicate a close correlation between the physical properties of the LSCO system and film thickness. In Section 2.1, the authors state that the film thickness used in this work is 90 nm, but they do not explain the rationale behind choosing 90 nm instead of

80 nm, 100 nm, or other thicknesses. Furthermore, when fitting the optical properties of LSCO using spectroscopic ellipsometry, the film thickness is inevitably required as an initial parameter. Therefore, it is strongly recommended that the authors appropriately zoom out the scale of the STEM image in Figure 1(b) to explicitly demonstrate that the actual film thickness is indeed 90 nm. This direct visual evidence would be much more convincing than merely estimating the thickness based on the number of PLD deposition pulses.

We thank the Reviewer for this helpful suggestion. We agree that film thickness can influence the physical properties of LSCO thin films through strain relaxation, defect formation, and substrate-related effects. In the present work, however, our objective was not to investigate thickness dependence, but rather to establish a consistent doping series with identical growth conditions so that the evolution of the electronic structure could be compared without introducing thickness as an additional variable. For this reason, all samples were prepared with the same thickness of ~90 nm under the same deposition conditions. This thickness was selected to ensure a consistent experimental platform across the entire doping series while providing sufficient signal quality for both spectroscopic and transport measurements. At the same time, it is sufficiently thick to reduce excessive substrate contributions while remaining compatible with stable epitaxial growth under our PLD conditions.

Importantly, the XRD, reciprocal-space mapping, and STEM data confirm that the 90 nm films remain highly crystalline and coherently aligned with the substrate across the full doping series, indicating that this thickness provides a suitable and reproducible platform for the present study. We have clarified this point in the revised manuscript.

In addition, following the Reviewer’s suggestion, we have also revised Figure 1(b) by expanding the field of view of the STEM image to more directly show the full film thickness.

Changes made to the Manuscript:

At the Reviewer’s request, the following sentence has been updated:

A uniform film thickness of ~90 nm was used for all LSCO samples in order to establish a consistent doping series and avoid introducing thickness as an additional variable. This thickness was chosen to ensure reliable spectroscopic and transport measurements while minimizing excessive substrate contributions. At the same time, it remains compatible with stable PLD growth and high epitaxial quality. Structural characterizations, including XRD, RSM, and STEM, confirm that films of this thickness are highly crystalline and coherently aligned with the substrate across the full doping range.

Cross-sectional scanning transmission electron microscopy (STEM) high-angle annular dark-field (HAADF) images, shown in Fig. 1(b), reveal the high crystalline quality of the films.

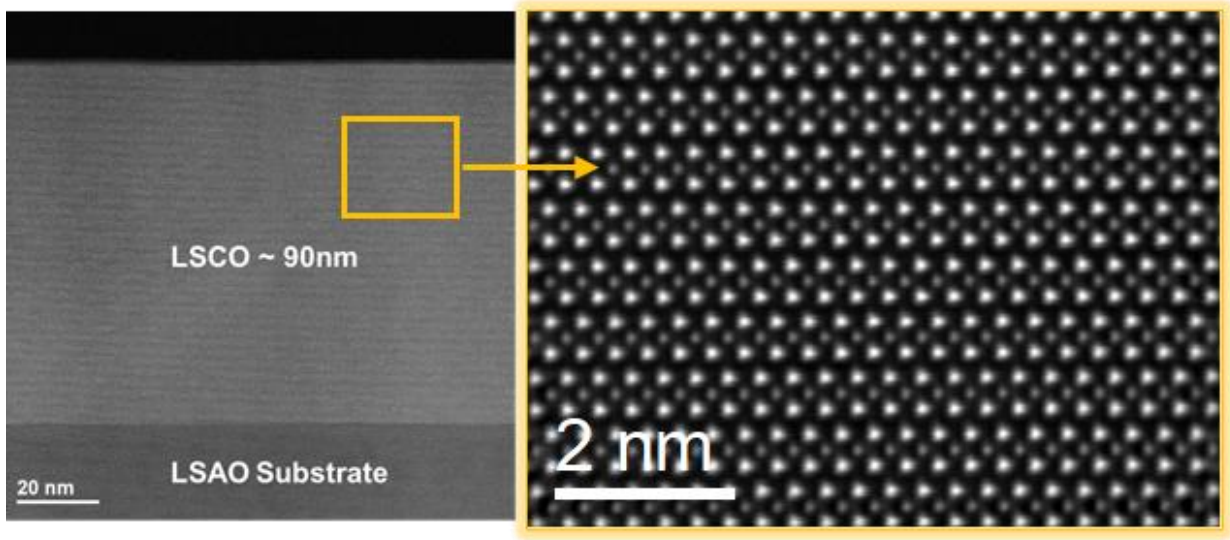

Fig 1(b). Atomic resolution HAADF-STEM images of the representative LSCO (x=0.15)/LSAO interface along the (001) zone axis.

2.The authors present XAS data collected at 300 K, while OSE data spans 5 K to 300 K, and DQMC simulations are performed at an effectively much higher temperature (β=10). The manuscript currently lacks a clear justification for this temperature mismatch. While it is physically reasonable that the high-energy scale electronic structure (eV range, such as ZRS breakdown) is dominated by doping rather than thermal fluctuations (meV range)—a point supported by the temperature-insensitivity of the OSE spectra —the authors must explicitly state this rationale in the main text. I recommend adding a brief discussion explaining why the 300 K XAS results and high-temperature DQMC simulations represent the intrinsic, normal-state electronic structure that directly correlates with the low-temperature OSE and transport findings.

We thank the Reviewer for this important suggestion. We agree that the temperature difference among the XAS, OSE, and DQMC results should be clarified explicitly in the manuscript. In the revised manuscript, we have clarified the rationale for the different temperature conditions used in the various measurements. The main discussion of the electronic structure is based on the 300 K XAS and OSE data, which were measured at the same temperature and therefore allow for direct comparison. The electronic-structure evolution discussed in this work occurs on an energy scale of approximately 1-2 eV, it is expected to be governed primarily by doping and electronic correlations rather than by thermal fluctuations at these temperatures. Therefore, the low-temperature OSE data are included only as supporting results in the Supplementary Materials.

In contrast, the transport measurements were performed over a broad temperature range in order to reflect the phase diagram of the material, since the physical properties differ substantially above and below the superconducting transition temperature. Importantly, the transport behavior observed in the normal-state region above Tc is consistent with the electronic-structure evolution inferred from the 300 K XAS and OSE results.

In response, we have added additional calculations of the local DOS at different temperatures for the same doping levels. The results show that the onset of low-energy spectral peak splitting is only weakly affected by temperature. This indicates that, within the accessible temperature range of our study, the key spectral evolution is largely robust against thermal effects.

Therefore, the available experimental and theoretical results consistently indicate that the

observed spectral features are governed predominantly by doping-induced electronic reconstruction rather than by temperature-dependent effects. We have added this discussion in the revised manuscript to clarify the validity of comparing results obtained at different temperatures within a unified normal-state framework.

Changes made to the Manuscript:

At the Reviewer's request, the following sentence has been updated:

Figs. S16-S17 are various-temperature OSE data of a series of LSCO ($0.15 \le x \le 0.6$) samples. Our OSE experimental results show similar ε_1, ε_2, and σ_1 among the different temperature points acrossing the superconducting transition temperature.

The main spectroscopic comparison is based on the 300 K XAS and OSE data, which show consistent doping-dependent evolution. The weak temperature dependence of the OSE spectra, together with additional temperature-dependent DQMC results, indicates that the key 1–2 eV spectral features are governed primarily by doping rather than thermal fluctuations. This supports the interpretation that the observed electronic-structure reconstruction is an intrinsic property of the normal state.

3.In the fifth line of the abstract, the subscripts in the chemical formula "La_2₋xSr_xCuO_4" are not formatted according to standard conventions. Such typographical errors should not appear in a formal manuscript. The authors should conduct a thorough and careful check throughout the entire text to correct this and any similar formatting issues.

We sincerely thank the Reviewer for the meticulous reading of the manuscript and for identifying this typographical issue. As suggested by the reviewer, we have corrected the "La_2₋xSr_xCuO_4" into "$La_{2-x}Sr_xCuO_4$" in the Abstract. This typographical error has been corrected in the revised manuscript. In addition, we have carefully proofread the entire manuscript to correct any remaining typographical, grammatical, and language issues.

Changes made to the Manuscript:

At the Reviewer's request, the following sentence has been updated:

In the Main Text:

Here, we track the evolution of the electronic structure from optimal to heavy overdoping in $La_{2-x}Sr_xCuO_4$ (LSCO) using broadband optical spectroscopy across x = 0.15–0.60. The measured spectral changes—including the redistribution of Zhang–Rice–related spectral weight—are in qualitative agreement with determinant quantum Monte Carlo (DQMC) simulations of the three-orbital Emery model, which together indicate a pronounced reconstruction of the electronic structure beyond hole concentrations $x > 0.2$. Guided by these observations, we propose a spontaneous checkerboard-type Zhang–Rice electronic configuration that accounts for the coexistence of itinerant and localized carriers characteristic of the heavily overdoped state.

Spectroscopic measurements reveal a saturation behavior which points to a fundamental

change in the electronic structure with further hole doping [Phys Rev Lett 103, 087402 (2009); Phys Rev B 88, 134525 (2013)]. Moreover, the validity of the ZRS approximation remains under debate, as a high-energy optical conductivity study reports a strong mixture of singlet and triplet configurations in the lightly hole-doped Zn-LSCO single crystal [Phys Rev B 95, 165108 (2017)].

Furthermore, our results predict a spontaneous formation of checkerboard-type Zhang–Rice (ZR) electronic configuration in the overdoped regime, offering new insight into the evolution of electronic correlations and the suppression of superconductivity.

To evaluate superconductivity across doping, the T_c for all samples was determined from electrical transport(resistivity) measurement (Figs.1(e-f)). The composition x = 0.15 corresponds the optimally hole-doped LSCO with the highest T_c of ~ 35 K.

Responses to the Reviewers

We are very grateful for the feedback and suggestions that Reviewers #1, #2 and #3 have provided. They are essential to make improvements to our manuscript. In the paragraphs below, we provide our responses to the respective remarks.

Note：All Reviewers' comments/questions are shown in black, while our corresponding responses are annotated in blue and the changes in red.

Reviewer #1 (Remarks to the Author):

The authors have revised their manuscript based on three referees' comments. The limit of numerical simulations and its weak point that connected with the experimental data has been discussed.

I think the rebuttal is reasonable. The proposed checkerboard picture offers a fresh microscopic viewpoint on the long-standing puzzle of the overdoped cuprate regime. After this major revision, incorporating the substantial new supplementary data and refined discussions, the manuscript may meet the standards for publication in Nature Communications.

We sincerely thank the Reviewer for the positive assessment and for recognizing the improvements made in the revised manuscript. We greatly appreciate the constructive comments, which have helped strengthen the quality of our work.

Reviewer #2 (Remarks to the Author):

I thank the authors for their detailed responses to my previous round of comments, however, a critical unresolved issue remains concerning the spectral fitting procedures that form the foundation of the manuscript's core conclusions, which I outline below.

The current presentation of fitting results for Figures 2e and 3d does not permit robust assessment of their reliability. Specifically, the figures did not display the total fitted curve overlaid on experimental data, with only individual component peaks, no quantification of agreement between the total fit and raw spectra, and no inclusion of residual plots to evaluate goodness-of-fit. This lack of transparency makes it impossible to verify whether decomposed spectral features (e.g., peaks Z, A*, A) are physically meaningful or artifacts of an inappropriate fitting routine.

We sincerely thank the Reviewer for the careful evaluation of our revised manuscript and for pointing out the remaining concerns regarding the transparency and reliability of the spectral fitting procedures. We agree that the fitting results presented in the previous version did not provide sufficient information for a direct assessment of the agreement between the experimental spectra and the total fitted curves. We have therefore comprehensively revised the spectral fitting analysis and substantially expanded the corresponding discussion in both the main text and the Supplementary Materials.

We would first like to clarify the role of spectral fitting in the present work. The central inference of doping-induced electronic-structure reconstruction is supported by several mutually consistent experimental observations and by the DQMC calculations, whereas the fitting is used primarily to parameterize the observed spectral evolution in terms of characteristic energies, linewidths, and spectral weights, and to enable a systematic comparison across the doping series.

In the O K-edge XAS data, Feature A* is manifested directly in the raw spectra as a low-energy shoulder that develops for $x > 0.2$. Its presence is also supported by the second-derivative and differential XAS analyses presented in the Supplementary Materials. Similarly, the optical conductivity spectra exhibit a pronounced redistribution of low-energy spectral weight in the over-doped regime. Together with the evolution of the dielectric response and transport properties, these observations are complemented by the DQMC calculations and collectively support the proposed electronic-structure reconstruction.

Therefore, the fitting is not used as the sole criterion for establishing the existence of these spectral features; rather, it provides a quantitative characterization of their evolution. Nevertheless, we fully agree with the Reviewer that such quantitative analysis should be demonstrated in a transparent and reproducible manner.

Accordingly, we have re-optimized the XAS and optical conductivity fitting procedures, and now show the experimental data, individual components, total fitted curves, and residual spectra explicitly. We have also added complete parameter tables and quantitative goodness-of-fit measures, including R^2 values for both the XAS and the optical conductivity analysis and RMSE values for the XAS analysis. These additions allow the quality and robustness of the spectral decomposition to be assessed directly.

We respectfully address the Reviewer's specific concerns below.

My specific concerns about fitting quality are as follows:

For $x = 0.15$ spectrum (Figure 2e): The raw spectral line shape does not conform to the symmetric profile of a standard Gaussian-Lorentzian (Voigt) function.

For $x = 0.5$ and $x = 0.6$ spectra (Figure 2e): The sum spectra (which is missing) of fitted components, obviously deviates from experimental data.

We thank the Reviewer for pointing out this important issue. After carefully re-examining our previous fitting procedure, we found that this asymmetry mainly originates from the overlap of Feature A with the broad high-energy spectral contribution that was not adequately described by the previously employed Shirley background, rather than requiring an intrinsically asymmetric line shape for Feature A itself.

In the previous analysis, a Shirley background was used for the O K-edge pre-edge region. However, this approach did not adequately capture the broad high-energy spectral contribution extending into the fitting window. As a result, the Shirley procedure redistributed the spectral weight unevenly across the fitting range, leading to an

overestimation of the intensity in some energy regions and an underestimation in others. Consequently, part of the high-energy contribution was not properly removed, which made Feature A to appear asymmetric and to be poorly described by a single symmetric Gaussian–Lorentzian function, especially for x = 0.15.

To address this problem, we have therefore revised the fitting strategy and now fit the XAS spectra directly, without prior Shirley-background subtraction. Relatively narrow Gaussian components are used to parameterize the experimentally resolved Features Z, A, and A*, while several broad Gaussian components describe the slowly varying high-energy spectral envelope that extends into the fitting window. Within the restricted fitting range, these broad components are treated collectively as an effective background contribution and are not assigned to additional discrete electronic excitations.

This treatment separates the broad high-energy contribution from Feature A within the fitting procedure and substantially improves the agreement with the measured line shape. Importantly, the revised fitting reproduces the same doping-dependent evolution of Features A and A* as the original analysis. The change in fitting protocol therefore improves the quantitative description of the spectra without changing the physical interpretation of their doping dependence.

Changes made to the Manuscript:

At the Reviewer's request, the following sentence has been updated:

In the main text:

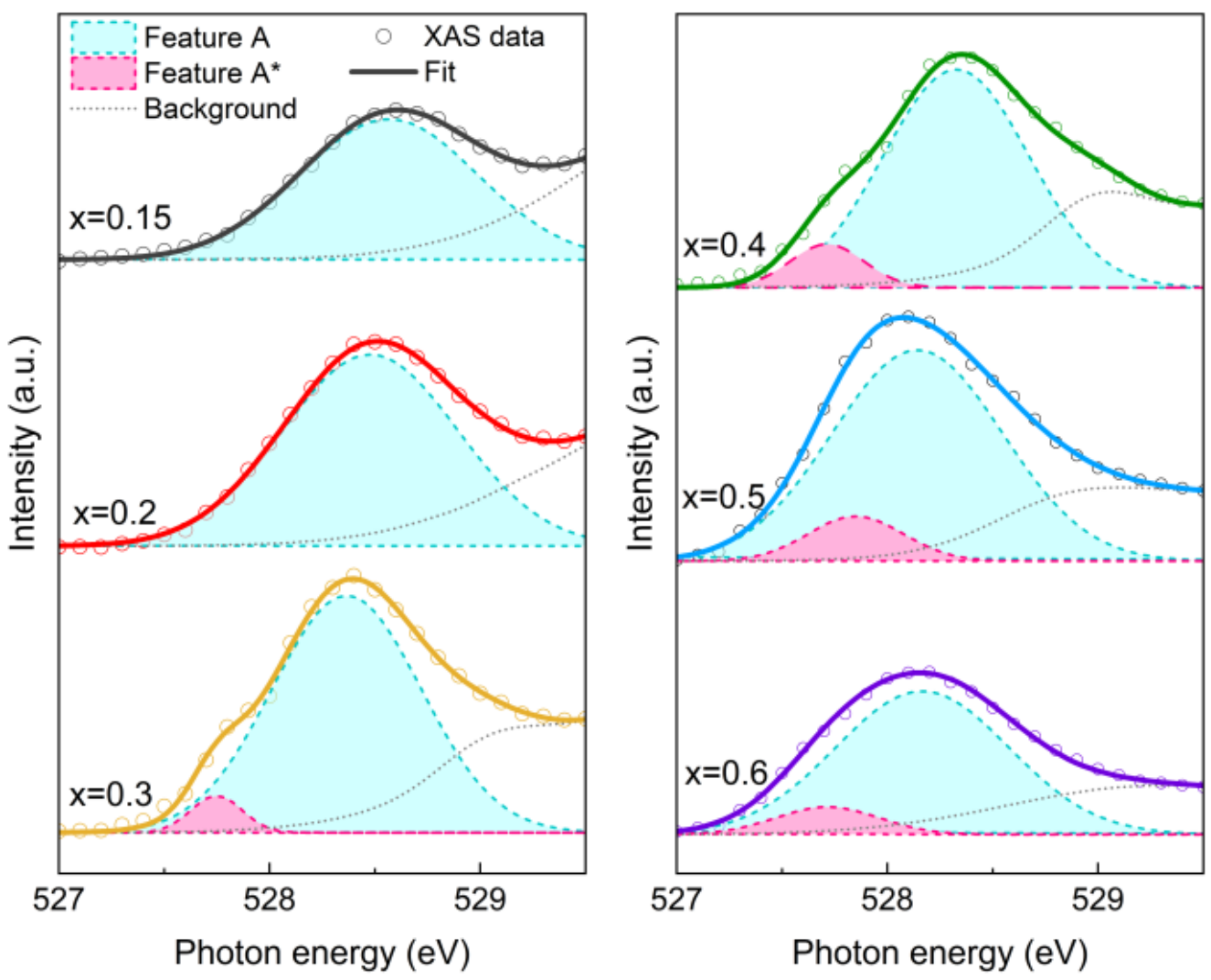

Fig. 2(e). Gaussian decomposition of the O K-edge XAS spectra in Fig. 2(c). Features *A** and *A* are represented by the pink- and light-blue-filled components, respectively, while the solid curve represents the total fitted spectrum.

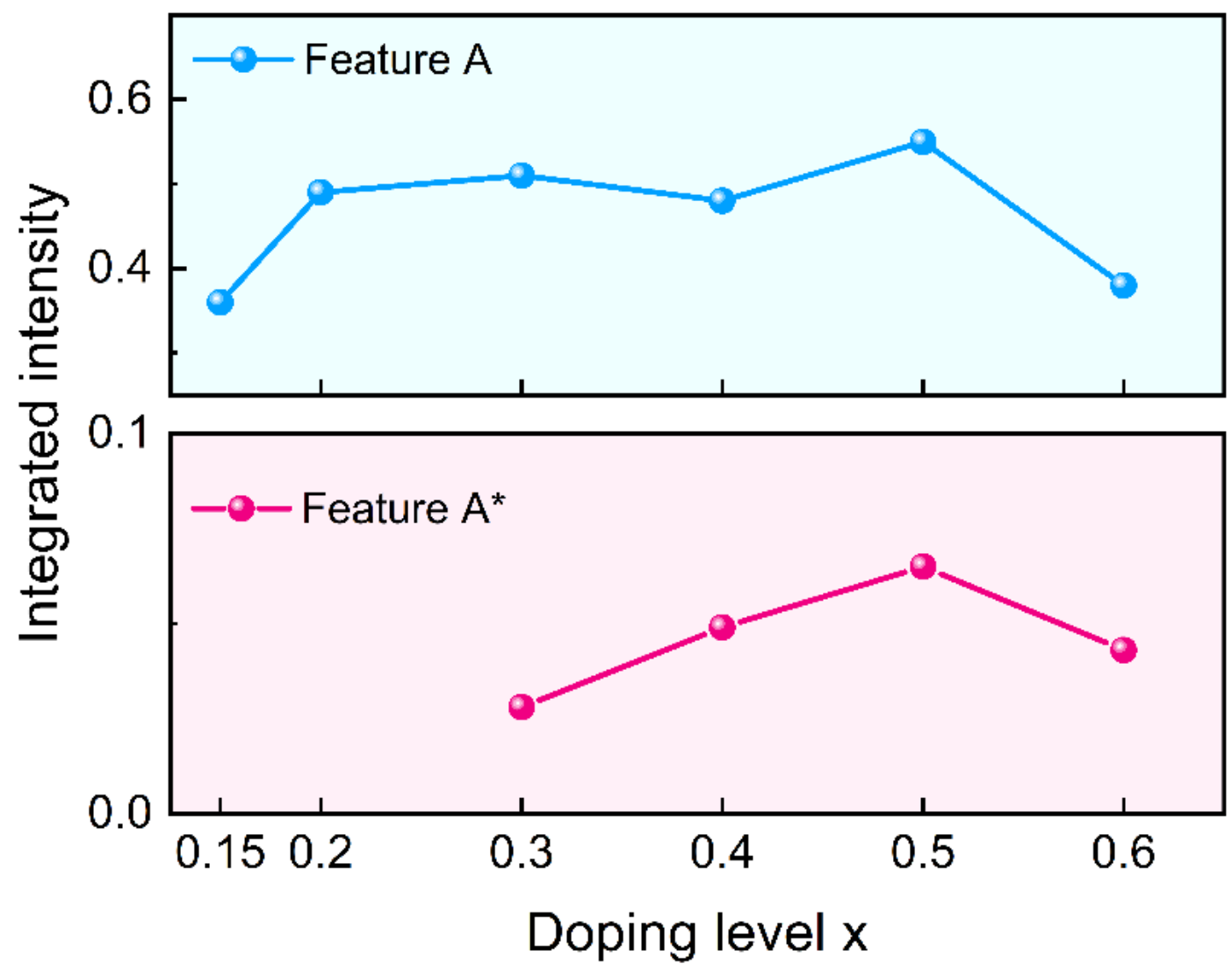

Fig. 2(f) Integrated intensities of Features *A** and *A* extracted from the 90° O K-edge XAS spectra as a function of doping concentration.

The corresponding discussion in Section 3.1 was also revised as follows:

"This systematic evolution is further substantiated by the second-derivative and differential XAS analyses presented in Supplementary Figs. S9–S11."

"To quantitatively characterize the doping evolution of the spectral features, the spectra were subsequently analyzed using the Gaussian decomposition shown in Fig. 2(e)."

In the Supplementary Materials:

To quantitatively characterize the doping evolution of Feature A*, the O K-edge pre-edge spectra were analyzed using a consistent fitting procedure across the entire doping series. The fitting results for the 90° geometry are presented in Fig. 2(e) of the main text, while the corresponding analysis for the 25° geometry is shown in Fig. S12(a).

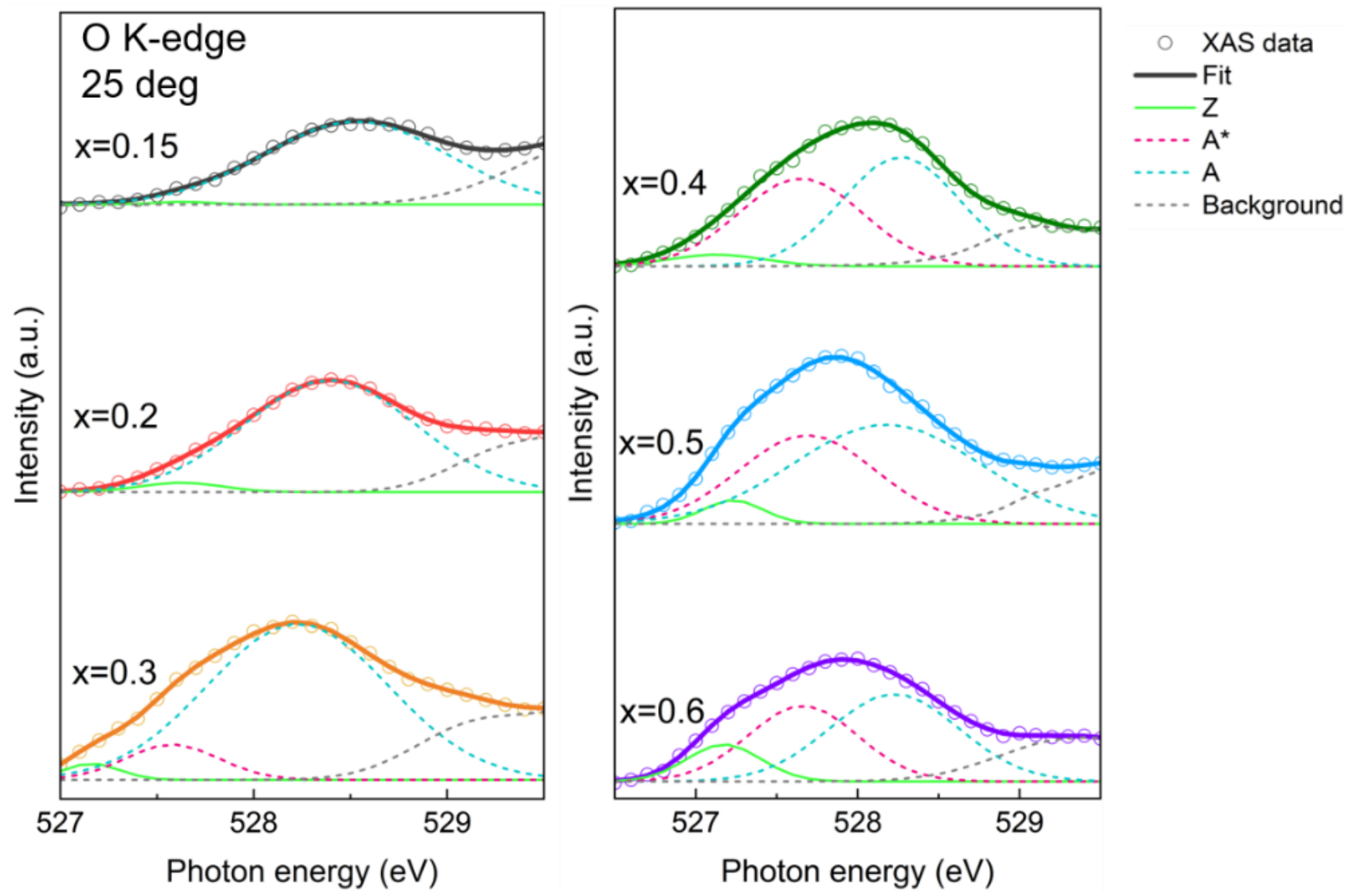

Fig. S12. (a) The fitting results of Fig.2d (in the main text) for identify the feature Z, A^* and *A*.

The XAS spectra were fitted using the Gaussian model. The purpose of this Gaussian-based parameterization is not to identify a unique microscopic broadening mechanism for each spectral feature, but rather to provide a stable and internally consistent quantitative description of the doping-dependent pre-edge spectral evolution. Relatively narrow Gaussian components were used to parameterize the experimentally resolved Features Z, A*, and A. In addition, several broad Gaussian components were introduced to describe the slowly varying high-energy spectral contribution extending into the selected fitting window. These broad components are treated collectively as an effective spectral background and are not assigned to additional discrete electronic excitations. The same fitting protocol was applied consistently across the entire doping series. The resulting center energies, FWHMs, integrated spectral weights, and goodness-of-fit metrics are summarized in Tables S1 and S2.

Figure 3d: Similar issues apply. There is no presentation of individual fit components, residuals, or quantitative agreement metrics, making it impossible to verify the fitting results that underpin the discussion of spectral evolution with doping.

We thank the Reviewer for this important comment. We agree that the previous presentation of the optical conductivity fitting did not provide sufficient information for a quantitative assessment of fit quality. We have therefore carefully re-examined and re-optimized the Drude–Lorentz analysis over the entire doping series.

In the previous analysis, the low-energy electrodynamic response was described primarily by a single Drude term. Although this term captures the dominant free-carrier response, it does not fully reproduce the detailed spectral curvature near the low-energy limit of the measured range, which in turn contributes to the residual of the total fit.

In the revised analysis, we therefore introduced an additional low-energy Lorentz oscillator together with the Drude component to account phenomenologically for the residual low-energy spectral contribution not captured by the ideal Drude response. The resonance energy of this additional oscillator lies substantially below the energy range of Features α and α^*, and its contribution is concentrated at the low-energy end of the spectrum. Therefore, this oscillator is introduced only to improve the description of the low-energy electrodynamic response and is not assigned to the interband transitions associated with α or α^*.

Including this additional low-energy component substantially improves the agreement between the calculated and measured optical conductivity, particularly at low energy, and reduces the systematic residuals. Because this oscillator is energetically well separated from Features α and α^*, the characteristic energies and systematic doping evolution of these higher-energy components remain essentially the same as in the previous analysis. Thus, the revised model improves the quantitative fit without altering the spectral assignments relevant to the proposed doping-induced reconstruction.

The revised manuscript now presents the measured optical conductivity $\sigma_1(\omega)$, the individual Drude and Lorentz contributions, the total calculated spectrum, and the corresponding residuals. In addition, R^2 values are provided for all doping concentrations in the Supplementary Materials.

We emphasize that the Drude–Lorentz analysis is used to quantify the doping-dependent redistribution of optical spectral weight. The measured $\sigma_1(\omega)$ spectra themselves show a pronounced evolution of the low-energy line shape and spectral weight for $x > 0.2$. The re-optimized model provides a more accurate parameterization of this evolution, while the emergence and systematic doping dependence of Features α^* and α remain consistent with the original analysis.

Changes made to the Manuscript:

At the Reviewer's request, the following sentence has been updated:

In the main text:

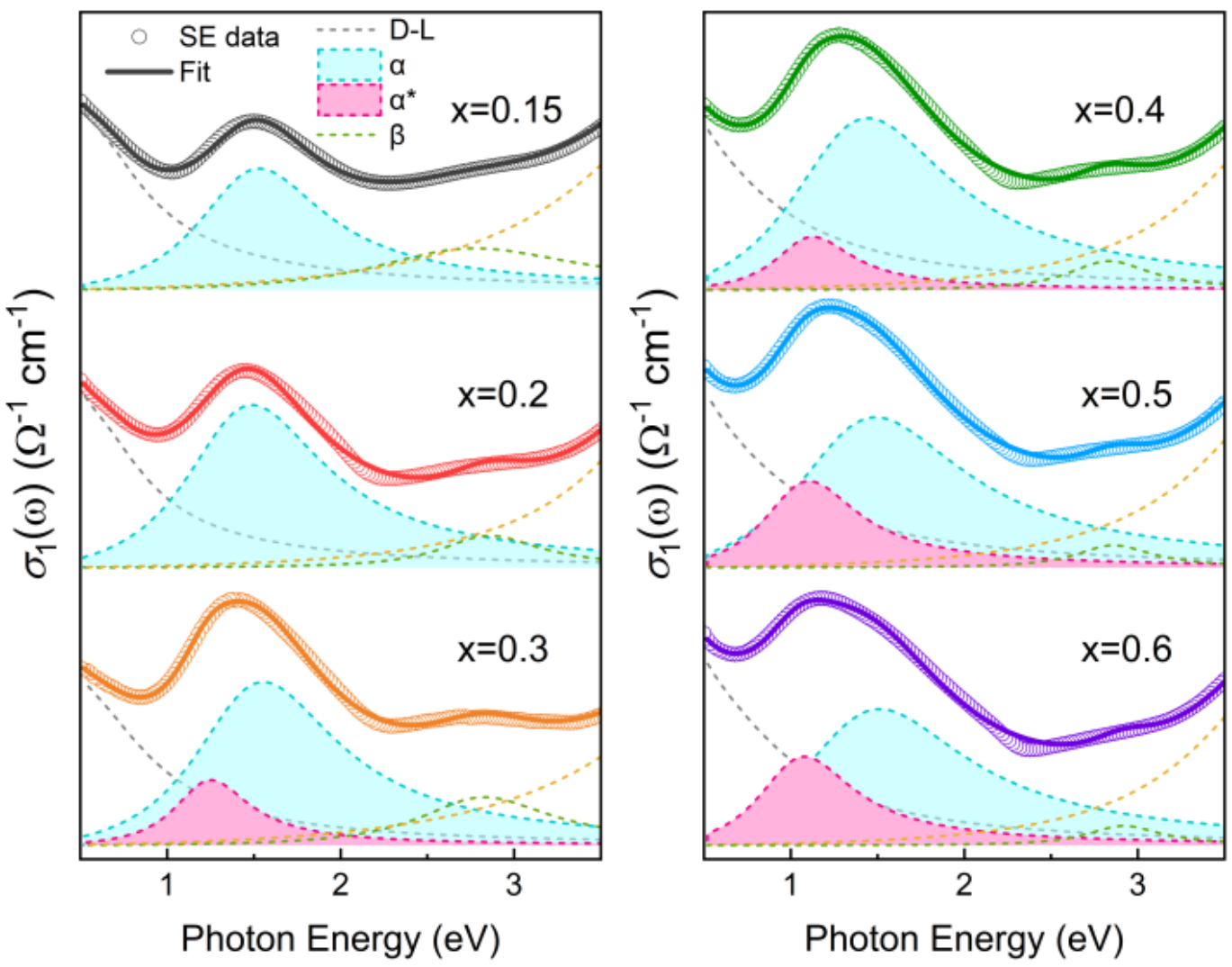

Fig. 3(d) represents the Drude-Lorentz fit, which is decomposed into a Drude component and several Lorentz components, α* and α, denoted by the pink filled and light bule filled regions, respectively.

In addition, the following description of the revised fitting model was added to Section 3.2:

"The Drude–Lorentz model was employed to fit the σ_1 spectra of all LSCO samples, enabling quantitative determination of the characteristic oscillator energies and spectral weights across the doping series [31]. In addition to the dominant Drude response, a low-energy Lorentz oscillator was included to account phenomenologically for the residual finite-frequency contribution near the lower-energy limit of the measured spectral range, which is not fully reproduced by an ideal Drude term. The detailed fitting procedure and complete model parameters are provided in Supplementary Section S4.3 and Table S3. The corresponding fitting curves are presented in Fig. 3(d). For $x \leq 0.2$, the σ_1 spectrum comprises a low-energy Drude-Lorentz term plus three Lorentz features."

In the Supplementary Materials:

(3) Fitting procedure

The optical conductivity spectra were analyzed using a consistent Drude–Lorentz model across the entire doping series. No independent empirical baseline was subtracted prior to fitting; instead, the measured electrodynamic response was described directly by the Drude and Lorentz contributions.

The dominant free-carrier response was represented by a Drude term. We found, however, that a single Drude contribution does not fully reproduce the spectral curvature near the low-energy limit of the measured range. Therefore, an additional low-energy Lorentz oscillator was included for all doping concentrations to account phenomenologically for the residual finite-frequency response not captured by the ideal Drude term. The resonance energy of this low-energy oscillator lies at approximately

0.5–0.7 eV, well below the characteristic energies of Features α^* and α. Its contribution is therefore treated as a correction to the low-energy electrodynamic response and is not assigned to either α^*or α.

Accordingly, the spectra for x = 0.15 and 0.2 were described using one Drude term, the additional low-energy Lorentz oscillator, the Lorentz components associated with Features α ,β.and high energy contribution. For $x \geq 0.3$, an additional Lorentz oscillator associated with Feature α^* was required to reproduce the experimentally observed redistribution of spectral weight near 1.1 eV. This fitting strategy provides a numerically stable and physically consistent description of the optical conductivity spectra while enabling direct comparison of spectral evolution across the full doping series.

The measured spectra, individual oscillator contributions, total fitted curves, and residuals are shown in Fig. 3(d) and Fig. S18(b), and the corresponding fitting parameters and R^2 values are summarized in Table S3.

This is not a minor technical point: the entire manuscript's narrative regarding electronic structure reconstruction in the heavily over-doped regime, the origin of the A* (and corresponding α* optical) feature, and the comparison to DQMC simulations rests entirely on the reliability of fitting results presented in Figures 2e and 3d. Without transparent documentation of the fitting workflow and convincing evidence that decomposed features match experimental data, these core conclusions lack a solid empirical foundation.

We appreciate the Reviewer for emphasizing the importance of establishing the robustness of the spectral analysis. However, we would respectfully clarify that the conclusion regarding the electronic-structure reconstruction does not rest entirely on the fitting results.

For the XAS measurements, the low-energy shoulder associated with Feature A* can already be observed directly in the experimental spectra for x > 0.2, and its characteristic energy is further supported by the second-derivative and differential XAS analyses. The fitting procedure is subsequently employed to quantify its spectral weight and doping dependence.

Likewise, the optical conductivity spectra show a systematic redistribution of low-energy spectral weight in the same doping regime. In addition, the anomalous doping evolution of the zero-crossing energy of ε_1, the evolution of the transport properties, and the DQMC-derived reconstruction of the low-energy electronic states provide mutually consistent evidence that is independent of the detailed peak decomposition.

Therefore, while we fully agree that the fitting procedure must be reliable and transparently documented, the physical conclusion is not contingent on a unique fitting line shape or on small variations in the extracted fitting parameters. This point is further demonstrated by the present re-analysis: after replacing the previous XAS fitting procedure with the revised Gaussian-based analysis and re-optimizing the optical conductivity fitting, the experimentally observed doping evolution and the central conclusions remain unchanged.

To address these concerns, I request the following revisions:

• Supplement Figures 2e and 3d with panels showing: (i) individual fitted component peaks, (ii) a direct point-by-point overlay of the total fitted curve and raw experimental data, and (iii) a residual spectrum (raw data minus total fit) to visualize systematic deviations.

• Provide a complete table of all fitting parameters in the Supplementary Materials, including for each decomposed peak: center energy, full width at half maximum (FWHM), integrated intensity, Gaussian-Lorentzian mixing ratio, and goodness-of-fit metrics (e.g., R^2, reduced χ^2).

We thank the Reviewer for this valuable suggestion. We have now added the complete fitting parameters for both the XAS and OSE analyses to the Supplementary Materials.

For the revised XAS analysis, the tabulated parameters include the center energy, FWHM, integrated intensity, and R^2 value for each spectrum.

For the optical conductivity analysis, the spectra are modeled using the Drude–Lorentz dielectric formalism rather than empirical Gaussian–Lorentzian peak profiles. We therefore provide the complete Drude and Lorentz parameters, including the resonance energies, oscillator amplitudes, broadening parameters, and R^2 value.

Because the revised XAS components are represented by pure Gaussian functions, a Gaussian–Lorentzian mixing ratio is no longer a fitting parameter and is therefore not tabulated. The complete parameters are now summarized in Tables S1–S3 to improve the transparency and reproducibility of the quantitative analysis.

Changes made to the Manuscript:

At the Reviewer's request, the following sentence has been updated:

In the Supplementary Materials:

Table S1 The fitting parameters of features A and A* in the O K-edge 90 deg XAS spectra obtained from fitting analysis.

LSCO sample O-K edge 90 deg	Feature A*			Feature A			R^2	RMSE
	Center energy (eV)	FWHM (eV)	Integrated intensity (eV)	Center energy (eV)	FWHM (eV)	Integrated intensity (eV)		
X=0.15	/	/	/	528.57	0.99	0.36	0.9993	0.004347
X=0.2	/	/	/	528.48	0.98	0.49	0.9990	0.004555

X=0.3	527.75	0.29	0.028	528.37	0.83	0.51	0.9961	0.009238
X=0.4	527.71	0.41	0.049	528.33	0.81	0.48	0.9956	0.01231
X=0.5	527.85	0.53	0.065	528.14	0.95	0.55	0.9980	0.007994
X=0.6	527.72	0.58	0.043	528.17	0.97	0.38	0.9975	0.006434

Table S2 The fitting parameters of features A, A* and Z in the O K-edge 25 deg XAS spectra obtained from fitting analysis.

LSCO sample O-K edge 25 deg	Feature Z			Feature A*			Feature A			R^2	RMSE
	Center energy (eV)	FWHM (eV)	Integrated intensity (eV)	Center energy (eV)	FWHM (eV)	Integrated intensity (eV)	Center energy (eV)	FWHM (eV)	Integrated intensity (eV)		
X=0.15	527.63	0.33	0.00095	/	/	/	528.53	1.11	0.10	0.9992	0.001735
X=0.2	527.62	0.50	0.0052	/	/	/	528.39	1.04	0.13	0.9995	0.000960
X=0.3	527.16	0.34	0.0061	527.58	0.57	0.022	528.23	1.10	0.19	0.9993	0.001347
X=0.4	527.13	0.69	0.0099	527.65	0.90	0.098	528.27	0.86	0.12	0.9967	0.002365
X=0.5	527.23	0.43	0.013	527.69	0.99	0.108	528.18	1.36	0.17	0.9985	0.001869
X=0.6	527.17	0.54	0.025	527.66	0.80	0.075	528.23	0.94	0.10	0.9991	0.001364

Table S3. The fitting parameters of Drude-Lorentz in the optical conductivity spectra obtained from fitting analysis.

		Amplitude (eV)	Broadening (eV)	Energy (eV)	R^2
X=0.15	Drude	A_D = 4.299 eV^2	B_D = 0.452 eV		0.9970
	Lorentz	1.440	0.523	0.672	
	Feature α	2.121	0.899	1.531	

	Feature β	0.401	1.473	2.774	
X=0.2	Drude	A_D = 4.532 eV^2	B_D = 0.301 eV		0.9950
	Lorentz	1.759	0.569	0.671	
	Feature α	2.921	0.981	1.491	
	Feature β	0.298	0.708	2.860	
X=0.3	Drude	A_D = 4.185 eV^2	B_D = 0.218 eV		0.9962
	Lorentz	2.725	0.729	0.650	
	Feature α*	1.394	0.534	1.253	
	Feature α	2.811	1.008	1.554	
	Feature β	0.455	0.940	2.828	
X=0.4	Drude	A_D = 3.990 eV^2	B_D = 0.148 eV		0.9968
	Lorentz	4.523	0.950	0.508	
	Feature α*	1.262	0.551	1.128	
	Feature α	3.181	1.129	1.445	
	Feature β	0.272	0.587	2.834	
X=0.5	Drude	A_D = 4.481 eV^2	B_D = 0.229 eV		0.9984
	Lorentz	2.407	1.027	0.642	
	Feature α*	2.098	0.670	1.105	
	Feature α	2.702	1.192	1.490	
	Feature β	0.208	0.541	2.860	
X=0.6	Drude	A_D = 4.487 eV^2	B_D = 0.343 eV		0.9967
	Lorentz	1.62	0.899	0.719	
	Feature α*	2.201	0.674	1.079	
	Feature α	2.411	1.178	1.512	
	Feature β	0.178	0.521	2.919	

For the Drude term, $A_D=(\hbar\omega_{p,D})^2$, $B_D=\hbar\gamma_D$

To quantitatively characterize the doping evolution of Feature A*, the O K-edge pre-edge spectra were analyzed using a consistent fitting procedure across the entire doping series. The fitting results for the 90° geometry are presented in Fig. 2(e) of the main text, while the corresponding analysis for the 25° geometry is shown in Fig. S12(b).

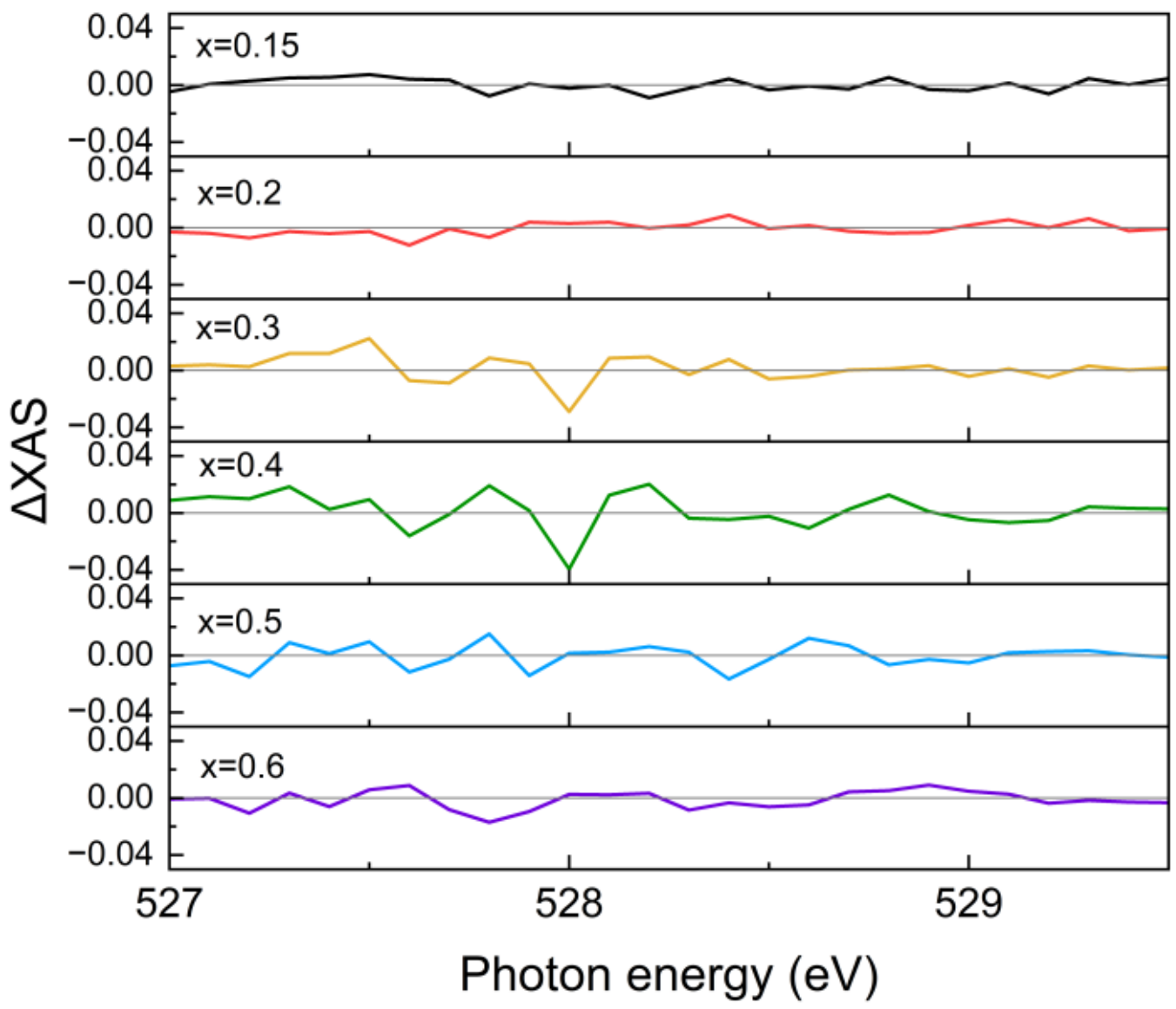

Fig. S12. (b) Residual spectrum, $\Delta XAS = I_{\mathrm{exp}} - I_{\mathrm{fit}}$, for the $90°$ O K-edge XAS spectra.

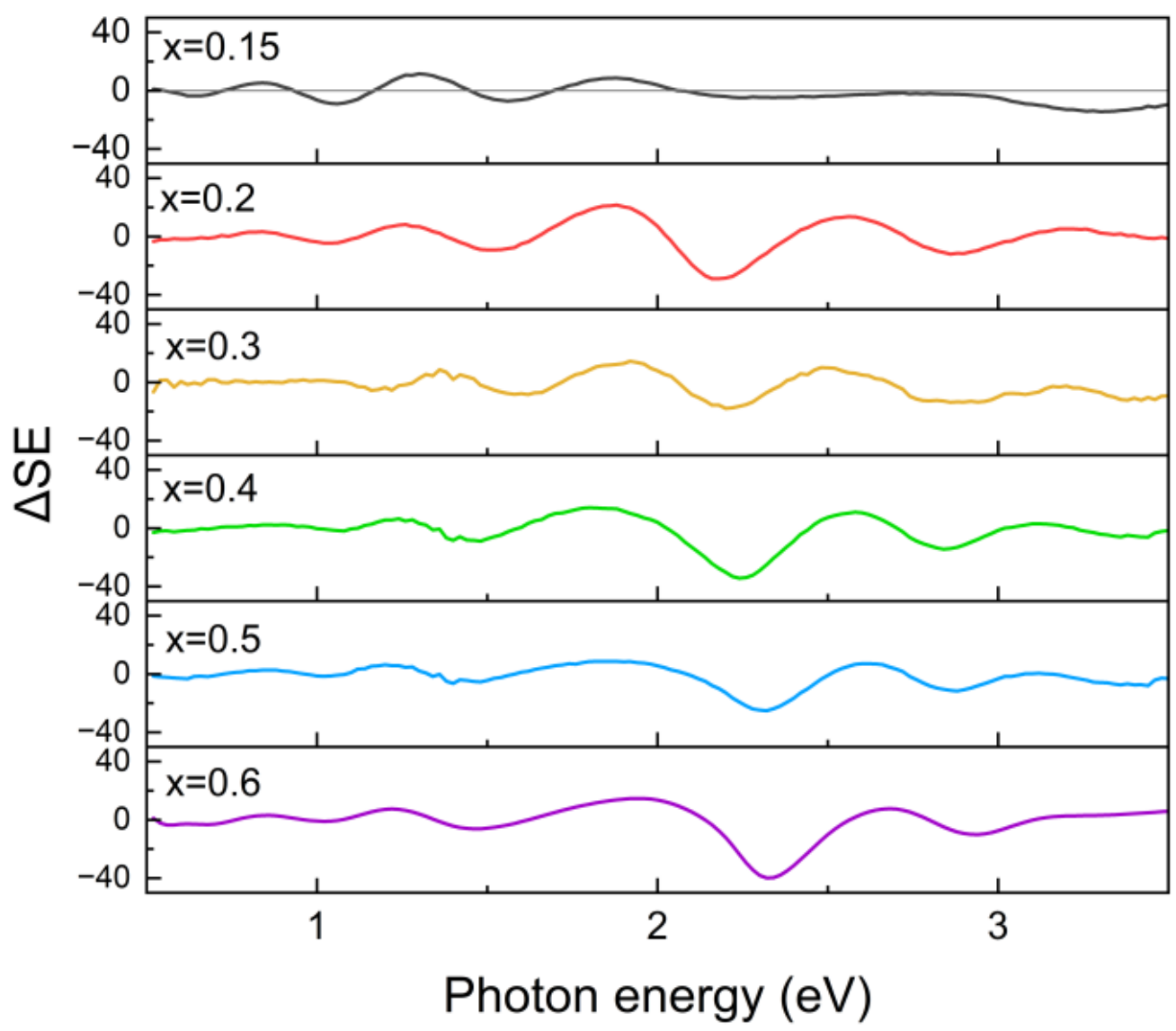

Fig. S18(b). Residual spectrum, $\Delta SE = \sigma_{1,\mathrm{exp}} - \sigma_{1,\mathrm{fit}}$, for x = 0.15-0.6.

• Include a brief methodological justification for the choice of Gaussian-Lorentzian line shapes, with a specific discussion of the x = 0.15 spectrum's asymmetry and tests of alternative line shape models to confirm robustness of peak assignments.

We thank the Reviewer for this suggestion. Rather than retaining the previous Gaussian–Lorentzian line shape, we have reconsidered the fitting procedure and adopted a simpler Gaussian-based parameterization for the revised XAS analysis.

The purpose of the XAS fitting in the present work is not to establish a unique microscopic broadening mechanism for each spectral feature, but to obtain a stable and consistent quantitative description of the doping-dependent pre-edge spectral evolution. Gaussian functions provide an adequate phenomenological description of the individual overlapping components within the experimental resolution, while broad high-energy Gaussian components account for the slowly varying spectral envelope.

In the revised treatment, the asymmetry of the (x=0.15) spectrum is accounted for by the superposition of Feature A and the broad higher-energy contribution, instead of assigning an intrinsically asymmetric line shape to Feature A

Importantly, the key peak assignment is robust against this modification of the fitting model. Feature A* is not required for x = 0.15 and x = 0.2, whereas a low-energy shoulder develops systematically in the raw spectra for x > 0.2. The corresponding second-derivative and differential XAS analyses independently identify the same spectral evolution. Therefore, the assignment of A* does not depend on the specific choice between Gaussian and Gaussian–Lorentzian line shapes.

The agreement among these independent analyses, together with the persistence of the same doping trend under the revised fitting model, demonstrates that the peak assignment is sufficiently robust to support the conclusions drawn here.

• Quantify the deviation between the total fitted curve and raw data for the x = 0.15, 0.5, and 0.6 spectra (e.g., root-mean-square error across the 526–529 eV window) to demonstrate that fit quality is acceptable for the claims being made.

We thank the Reviewer for this valuable suggestion. In response, we have now quantitatively evaluated the deviation between the experimental XAS spectra and the optimized total fitted curves and using the root-mean-square error (RMSE) over the 527–529.5 eV energy range.

The RMSE values for x = 0.15, 0.2, 0.3, 0.4, 0.5, and 0.6 are 0.004347, 0.004555, 0.009238, 0.01231, 0.007994, and 0.006434, respectively. Across the complete doping series, the RMSE remains within a relatively narrow range, showing that the deviations between the experimental spectra and the optimized fits remain on the order of 10^{-3}–10^{-2}. Importantly, the fitting error does not systematically increase in the heavily over-doped regime. In fact, the RMSE values for x = 0.5 and x = 0.6 are lower than those for x = 0.3 and x = 0.4. This quantitative comparison confirms that the revised fitting provides a consistently good description of the experimental spectra across the entire doping series,

including the x = 0.5 and x = 0.6 spectra specifically questioned by the Reviewer.

Once these revisions are completed and the reliability of fitting results is clearly demonstrated, I will be able to reassess the manuscript’s suitability for publication. I appreciate the authors’ efforts to address prior feedback, and look forward to evaluating the updated version.

Reviewer #3 (Remarks to the Author):

After being revised, the author of the paper has corrected the relevant errors and answered my questions. At the same time, I also carefully read the author's responses to the questions raised by other reviewers. I believe that this paper has strong theoretical and innovative qualities, and its current overall level has basically met the publication requirements of the journal.

We sincerely thank the Reviewer for the positive assessment and for recognizing the improvements made in the revised manuscript. We greatly appreciate the constructive comments, which have helped strengthen the quality of our work.
